# Cuproptosis‐associated lncRNA impact prognosis in patients with non‐small cell lung cancer co‐infected with COVID‐19

**DOI:** 10.1111/jcmm.70059

**Published:** 2024-09-03

**Authors:** Jing Li, Nan Wang, Guocai Mao, Jiantang Wang, Mengqi Xiang, Huachuan Zhang, Daxiong Zeng, Haitao Ma, Junhong Jiang

**Affiliations:** ^1^ Department of Respiratory and Critical Care Medicine The Fourth Affiliated Hospital of Soochow University, Suzhou Dushu Lake Hospital, Dushu Lake Hospital Affiliated to Soochow University, Medical Centre of Soochow University Suzhou Jiangsu China; ^2^ Department of Thoracic Surgery The Fourth Affiliated Hospital of Soochow University, Suzhou Dushu Lake Hospital, Dushu Lake Hospital Affiliated to Soochow University, Medical Centre of Soochow University Suzhou Jiangsu China; ^3^ Department of Thoracic Surgery The First Affiliated Hospital of Soochow University, Soochow University Suzhou Jiangsu China; ^4^ Department of Medical Oncology Sichuan Cancer Hospital, Medical School of University of Electronic Science and Technology of China Chengdu Sichuan China; ^5^ Department of Thoracic Surgery Sichuan Cancer Hospital, Medical School of University of Electronic Science and Technology of China Chengdu Sichuan China; ^6^ Department of Respiratory and Critical Care Medicine The First Affiliated Hospital of Soochow University, Soochow University Suzhou Jiangsu China

**Keywords:** COVID‐19, cuproptosis, lncRNA, NSCLC

## Abstract

Non‐small cell lung cancer (NSCLC) patients infected with COVID‐19 experience much worse prognosis. However, the specific mechanisms behind this phenomenon remain unclear. We conducted a multicentre study, collecting surgical tissue samples from a total of 36 NSCLC patients across three centres to analyse. Among the 36 lung cancer patients, 9 were infected with COVID‐19. COVID‐19 infection (HR = 21.62 [1.58, 296.06], *p* = 0.021) was an independent risk factor of progression‐free survival (PFS). Analysis of RNA‐seq data of these cancer tissues demonstrated significantly higher expression levels of cuproptosis‐associated genes in COVID‐19‐infected lung cancer patients. Using Lasso regression and Cox regression analysis, we identified 12 long noncoding RNAs (lncRNA) regulating cuproptosis. A score based on these lncRNA were used to divide patients into high‐risk and low‐risk groups. The results showed that the high‐risk group had lower overall survival and PFS compared to the low‐risk group. Furthermore, Tumor Immune Dysfunction and Exclusion (TIDE) database revealed that the high‐risk group benefited more from immunotherapy. Drug sensitivity analysis identified cetuximab and gefitinib as potentially effective treatments for the high‐risk group. Cuproptosis plays a significant role NSCLC patients infected with COVID‐19. Promisingly, cetuximab and gefitinib have shown potential effectiveness for managing these patients.

## 1 INTRODUCTION

Lung cancer, characterized by the highest incidence among all tumour types, is the primary cause of cancer‐related mortality across the globe. In 2018 alone, there were approximately 2.1 million newly diagnosed cases and 1.8 million deaths attributed to lung cancer.^1^ Non‐small cell lung cancer (NSCLC) constitutes nearly 85% of all lung cancer subtypes. Despite advancements in median survival time, with an increase from 9 to 11 months, as well as improvements in 5‐year survival rates, the long‐term survival rates for NSCLC remain notably low.^2^

Patients with lung cancer face a higher risk of mortality from SARS‐CoV‐2 infection compared to other cancer types.^3^ Although profound immunosuppressive therapy is not commonly employed in lung cancer treatment, these patients exhibit increased susceptibility to severe lung injury, pulmonary complications and elevated mortality rates due to COVID‐19. Several factors contribute to this vulnerability, including the pathophysiology, clinical characteristics and treatment approaches associated with lung cancer.^4^ The combination of factors commonly observed in lung cancer patients, such as smoking‐induced lung damage, significant cardiovascular and respiratory comorbidities and advanced age, contributes to the severity of SARS‐CoV‐2 infection.^4^, ^5^, ^6^ Additionally, lung cancer patients may have underlying fragility caused by defective pulmonary and alveolar architecture resulting from prior thoracic surgery or radiotherapy, as well as malignant airway obstruction, which further enhances their susceptibility to severe infections. The alterations in the alveolar epithelium and pulmonary vessels not only affect the tumour microenvironment but also lead to increased infiltration of immune cells and tissue‐resident macrophages, thereby influencing innate immunity.^7^, ^8^

Cuproptosis is a unique form of cellular demise that distinguishes itself from well‐known mechanisms like apoptosis, autophagy and ferroptosis. It is specifically triggered by copper ions and operates through direct interaction with lipoacylated components of the tricarboxylic acid cycle within mitochondrial respiration. Consequently, this process leads to the accumulation of lipoacylated proteins and a decline in Fe‐S cluster protein levels, initiating a stress response due to protein toxicity, ultimately culminating in cell death.^9^ Notably, a study conducted by Zhou et al. demonstrated significantly higher levels of cuproptosis in patients with COVID‐19 compared to those without the infection.^10^

Long noncoding RNAs (lncRNAs) have garnered considerable interest in the field of cancer research. These RNA molecules, exceeding 200 nucleotides in length, do not possess the ability to code for proteins.^11^ They serve essential functions in modulating chromosomal alterations, regulating transcription and participating in various cellular processes.^12^ However, there is a paucity of studies examining cuproptosis‐related lncRNAs and their prognostic implications in NSCLC patients with COVID‐19 infection.

In this study, we conducted a multicentre study, collecting surgical tissue samples from a total of 36 NSCLC patients across three centres. We successfully extracted RNA and performed RNA‐seq analysis on 30 cases to investigate copper death‐associated factors. Furthermore, we validated our findings using lung cancer data from the TCGA database and analysed immunotherapy response and drug sensitivity data. We aim to identify additional drug targets for patients combined with NSCLC and COVID‐19 through our research.

## 2 MATERIALS AND METHODS

### 2.1 Patients

In this retrospective real‐world study, we conducted an analysis using data from 36 patients diagnosed with NSCLC who underwent surgical procedures at three different hospitals: Dushu Lake Hospital Affiliated to Soochow University, the First Affiliated Hospital of Soochow University and Sichuan Cancer Hospital. The study duration spanned from 1 August 2021 to 31 July 2022. NSCLC diagnoses were confirmed through comprehensive pathological assessments, ensuring accurate diagnosis and classification of the disease.

Collected data encompassed a wide range of patient parameters, including age, gender, body mass index (BMI), smoking status, pathological type, clinical staging, surgical procedure details, COVID‐19 infection status and survival outcomes. The median value was used as the cutoff point for classification of continuous variables such as age and BMI, providing a standardized method for categorizing patients.

Patients were categorized as COVID‐19 positive if they tested positive for the virus based on both imaging (such as chest CT scans) and virological assessments (RT‐PCR tests). This dual approach ensured accurate identification of COVID‐19 infection among the NSCLC patients.

Prior to conducting the study, informed consent was obtained from all patients or their immediate family members to ensure adherence to ethical standards. The research protocol strictly followed the guidelines set forth by the Ethics Committee of Soochow University and the principles stated in the Declaration of Helsinki, ensuring the ethical conduct of the study. Patients' confidentiality and privacy were maintained throughout the study, and data were anonymized to protect patient identities.

This thorough and ethically sound approach provided a robust dataset for analysing the impact of COVID‐19 on NSCLC patients, with particular attention to clinical outcomes and survival rates. The integration of diverse patient parameters allowed for a comprehensive analysis, contributing to a better understanding of the interplay between NSCLC and COVID‐19.

This expanded version provides more detailed information about the patient cohort, data collection methods and ethical considerations, giving a clearer picture of the study's design and execution.

### 2.2 Sample collection and RNA‐seq analysis

Samples were collected from lung cancer patients during the surgical procedure and preserved by immersing them in a solution specifically designed for RNA storage. The obtained cellular samples, which yielded approximately 20–30 ng of mRNA, were utilized to generate RNA‐seq libraries using the KAPA Stranded mRNA‐Seq Kit suitable for the Illumina platform. Paired‐end sequencing of the libraries was conducted on the Illumina HiSeq‐PE150 instrument.

### 2.3 Transcriptomic data, mutation data and clinical information of TCGA

The transcriptome profiling dataset for RNA‐seq analysis consisted of 481 samples obtained from patients diagnosed with lung adenocarcinoma (LUAD). These samples were sourced from the Cancer Genome Atlas (TCGA) database (https://portal.gdc.cancer.gov/) on 20 April 2022. The dataset includes comprehensive clinical and molecular information, allowing for an in‐depth analysis of the association between lncRNA expression and patient prognosis. The dataset includes patients across various clinical stages of LUAD, ranging from stage I to stage IV, enabling the analysis of lncRNA expression patterns across different stages of disease progression. The patients' ages range from 30 to 85 years, with a median age of 65, and the cohort includes both male and female patients, with a nearly equal gender distribution, ensuring that the analysis is not biased towards a particular gender. Information on patients' ethnicity and smoking status is also included, providing additional variables for stratification and analysis, which is crucial for understanding the influence of genetic and environmental factors on lncRNA expression. Additionally, the dataset encompasses different histological subtypes of LUAD, enabling the exploration of subtype‐specific lncRNA signatures. Comprehensive survival data, including overall survival (OS) and progression‐free survival (PFS), is available, facilitating the assessment of lncRNA prognostic value. The RNA‐seq data were processed using standard bioinformatics pipelines; raw sequencing reads were aligned to the human reference genome (GRCh38) using the STAR aligner. The expression levels of lncRNAs were quantified using HTSeq‐count and normalized to account for sequencing depth and gene length. Differential expression analysis was performed using DESeq2, identifying lncRNAs significantly associated with clinical outcomes. The annotations for lncRNAs were acquired from the GENCODE website (https://www.gencodegenes.org/). Additionally, a set of genes associated with cuproptosis was identified based on a previous study conducted by Tsvetkov et al. in 2022.^9^

### 2.4 Generation and evaluation of long noncoding RNAs associated with cuproptosis

To identify cuproptosis‐associated lncRNAs, we performed coexpression analysis using the ‘limma’ package in R. This analysis involved assessing the correlation between lncRNAs and cuproptosis‐related genes by applying criteria of |Cor|>0.4 and *p* < 0.001. Based on the results obtained from the coexpression analysis, we utilized various R packages including ‘ggplot2’, ‘ggalluvial’ and ‘dplyr’ to generate a Sankey plot. This plot visually illustrates the relationship between cuproptosis‐related genes and lncRNAs.

### 2.5 Cox model construction

The training and validation groups for the samples were generated by random allocation using the ‘caret’ package in R. Survival data analysis was performed for each cuproptosis‐related lncRNA using univariate Cox proportional hazards regression, employing the R package ‘survival’. To avoid overfitting, we applied Lasso‐penalized Cox regression with the ‘glmnet’ package in R software. Optimal penalty (*λ*) criteria were selected based on 10‐fold cross‐validation.

Furthermore, multiple stepwise Cox regression analyses were conducted to identify the Cuproptosis‐Related Long Noncoding RNA Prognostic Model (CRLPM). The risk scoring model was constructed using the following formula: Lasso Risk Score = ∑*i* = 1n Coefi × *xi*, where Coefi represents the coefficients and *xi* represents the normalized count of each cuproptosis‐related lncRNA. Based on this Lasso prognostic model, patients can obtain a risk score.

### 2.6 Validation of the data

Both the training and validation groups were stratified into high‐ and low‐risk categories based on the median risk score derived from the corresponding coefficient of the training group. The prognostic performance of the CRLPM was evaluated using the Kaplan–Meier method in both groups. To assess the accuracy and diagnostic value of the model, receiver operating characteristic (ROC) curves were generated, and the area under the curve (AUC) values were calculated using the survival ROC and time ROC packages in R. Principal component analysis (PCA) was conducted to further validate the risk models, and the results were visualized using the ‘scatterplot3D’ package in R software. PFS analysis was performed using the ‘survival’ and ‘survminer’ packages in R. The accuracy of the risk models was predicted using the C‐index with the help of the R packages ‘rms’, ‘dplyr’, ‘survival’ and ‘pec’. The validation process was carried out on both the validation and entire cohorts to validate the effectiveness of the model.

### 2.7 Nomogram establishment

To evaluate the prognostic significance of the risk model, we performed both univariate and multivariate Cox regression analyses. The independent prognostic value of the risk model was determined based on the results obtained from these analyses.^13^ Subsequently, using the R packages ‘rms’, ‘regplot’ and ‘survival’, we constructed a nomogram incorporating the findings from the univariate and multivariate Cox regression analyses. This nomogram serves as a visual tool for predicting patient outcomes.

### 2.8 Association between prognostic risk score and clinical stage

To evaluate the generalizability of the model across different clinical stages, we explored the relationship between the risk score and clinical stage in patients with NSCLC. Univariate and multivariate Cox regression analyses were conducted to investigate the potential contributions of both the risk score and clinical stage.

### 2.9 Pathway and functional analysis

Differentially expressed genes (DEGs) between the high‐ and low‐risk groups were identified using the ‘limma’ package in R. The criteria for selecting DEGs were set as log2|fold change|>1 and false discovery rate <0.05. To gain insights into the biological functions and signalling pathways associated with these DEGs, we utilized R packages such as ‘clusterProfiler’, ‘org.Hs.eg.db’ and ‘enrichplot’ to explore Gene Ontology (GO) terms and Kyoto Encyclopedia of Genes and Genomes (KEGG) pathways.

### 2.10 Estimation of tumour‐infiltrating immune cells and immunotherapy

To investigate the association between the CRLPM risk score and immune cell infiltration, we employed the single‐sample gene set enrichment analysis (ssGSEA) algorithm in R. This analysis allowed us to assess the infiltration and functional status of tumour‐infiltrating immune cells. The results were visualized using a heat map. Additionally, we utilized the Tumor Immune Dysfunction and Exclusion (TIDE) algorithm, accessible at http://tide.dfci.harvard.edu,^14^ to predict the response to immunotherapy by simulating tumour immune escape mechanisms. The TIDE algorithm was applied to evaluate the impact of immunotherapy in both the high‐ and low‐risk groups.

### 2.11 Drug sensitivity assessment

To evaluate the clinical potential of the CRLPM in LUAD treatment, we determined the semi‐inhibitory concentration (IC_50_) of various chemotherapeutic drugs. This calculation was performed using the ‘pRRophetic’ R package along with its associated dependencies, including ‘car’, ‘ridge’, ‘preprocessCore’, ‘genefilter’ and ‘sva’. A total of 138 drugs, such as midostaurin, temsirolimus, tipifarnib and imatinib, were included in the analysis. To compare the differences in IC_50_ values between common antineoplastic agents in the high‐ and low‐risk groups, we conducted the Wilcoxon signed‐rank test. The results were visualized using a boxplot generated with the R package ‘ggplot2’.

### 2.12 Calculation of Tumour Mutational Burden (TMB) score

TMB quantifies the number of mutations present in a cancer genome. In this study, we utilized the R package ‘maftools’ to analyse mutation data from NSCLC samples obtained from TCGA.^15^ The association between risk scores and TMB in LUAD patients was depicted using a waterfall diagram for visual representation.

### 2.13 Statistical analysis

**FIGURE 1:**
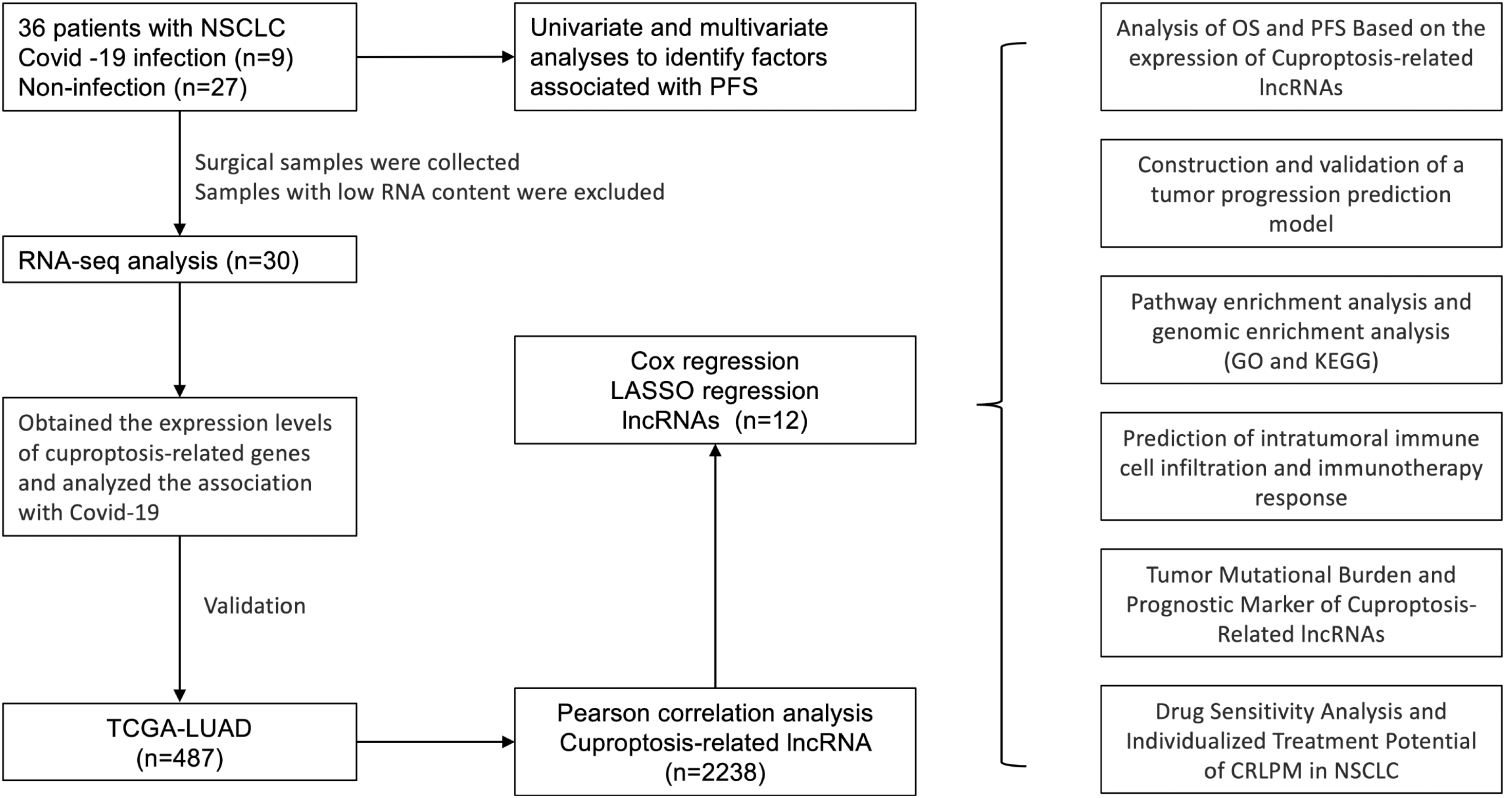
Flow chart of the study design.

Missing values (≤5.0%) were imputed using the random forest method implemented in the ‘mice’ package within RStudio (R version 4.3.2). Categorical variables were presented as proportions and assessed using the *κ* ^2^ test for comparison. Continuous variables were described as either median with mean ± standard deviation or quartile range, depending on their distribution. Group comparisons were conducted using one‐way anova (for normally distributed variables) or Kruskal–Wallis tests (for skewed variables), followed by appropriate post hoc tests for pairwise comparisons among the four stages. Kaplan–Meier curves were utilized to visualize cumulative mortality, while log‐rank tests were used to assess survival differences. Univariate and multivariate Cox regression models were employed to adjust for survival responses and estimate OS. The significance of prognostic covariates was presented using forest plots. Constrained cubic spline alignments were performed using the ‘rms’ package in R to analyse functional relationships. Functional enrichment analysis of GO terms and KEGG pathways was carried out using the ‘clusterProfiler’ package. Heat maps for cluster analysis were generated using the ‘Pheatmap’ package. Differences between two groups of quantitative data were analysed using the Wilcoxon rank‐sum test. To establish a prognostic risk model, Cox multifactor regression models were applied to identify risk factors, with variants having *p*‐values below 0.05 included in the model. The weight of each variant was quantified, and nomograms were created. Internal validation was performed using 1001 bootstrap resamples, and calibration tests were conducted to evaluate model concordance. Decision curve analysis was utilized to assess the clinical benefit of the model compared to traditional prognostic scoring based on clinical factors. Associations between factors and survival outcomes were analysed using log‐rank tests and Kaplan–Meier curves. All statistical analyses were carried out using RStudio (R version 4.3.2) and various R packages including ‘rms’, ‘ggplot2’, ‘risk regression’, ‘PredictABLE’ and ‘survminer’. The flowchart in Figure 1 illustrates the logical sequence of the statistical analysis conducted in the article.

## 3 RESULTS

### 3.1 Clinical analysis

A total of 36 patients diagnosed with NSCLC were included in this study, spanning from 1 August 2021, to 31 July 2022. None of the patients have experienced mortality at the time of analysis, but disease progression was observed in 4 patients (11.0%). Among the enrolled patients, 22 (61.0%) were male, with a median age of 69 years (IQR 64–75 years). It is noteworthy that 22 individuals (61.0%) had a history of smoking. Furthermore, 19 (53.0%) patients had an ECOG performance status score of 1. The histological subtypes were distributed as follows: 28 (78.0%) cases of adenocarcinoma and 8 (22.0%) cases of squamous cell carcinoma. All patients underwent surgical intervention, with 30 (84.0%) classified as stage I‐II and 6 (16.0%) classified as stage III‐IV according to Table 1. Univariate and multivariate analyses demonstrated that COVID‐19 infection (HR = 21.62 [1.58, 296.06], *p* = 0.021) and disease stage (HR = 23.01 [1.76, 300.30], *p* = 0.017) independently influenced disease progression in lung cancer patients, as shown in Table 2.

**TABLE 1:** Study participant characteristics at enrolment.

Variables	Total (*n* = 36)	Cohort, median (IQR)		*p*‐value
		Infection (*n* = 7)	Non‐infection (*n* = 29)	
Age, median (Q1, Q3)	69 (64, 75)	67 (64, 74)	71 (65, 75)	0.749
Gender, *n* (%)				1
Female	14 (39)	3 (43)	11 (38)	
Male	22 (61)	4 (57)	18 (62)	
BMI, mean ± SD	23.96 ± 3.31	24.02 ± 3.88	23.95 ± 3.23	0.96
ECOG/PS, *n* (%)				0.684
0	17 (47)	4 (57)	13 (45)	
1	19 (53)	3 (43)	16 (55)	
Smoking, *n* (%)				**0.011**
Current	10 (28)	1 (14)	9 (31)	
Former	12 (33)	0 (0)	12 (41)	
Never	14 (39)	6 (86)	8 (28)	
Histology, *n* (%)				1
LUAD	28 (78)	6 (86)	22 (76)	
LUSC	8 (22)	1 (14)	7 (24)	
Stage, *n* (%)				0.384
I	25 (69)	5 (71)	20 (69)	
II	5 (14)	0 (0)	5 (17)	
III	3 (8)	1 (14)	2 (7)	
IV	3 (8)	1 (14)	2 (7)	
Type of surgery, *n* (%)				1
Lobectomy	28 (78)	6 (86)	22 (76)	
Pneumonectomy	8 (22)	1 (14)	7 (24)	
PFS status, *n* (%)				**0.018**
Remmission	32 (89)	4 (57)	28 (97)	
Progression	4 (11)	3 (43)	1 (3)	

Abbreviations: BMI, body mass index; ECOG, Eastern Cooperative Oncology Group; IQR, interquartile range; LUAD, lung adenocarcinoma; LUSC, lung squamous cell carcinoma; PFS, progression‐free survival; PS, performance status. Bold values indiacates *P* < 0.05.

**TABLE 2:** Cox regression model for PFS.

Variables	Univariate analysis		Multivariate analysis	
	HR	*p*‐value	HR	*p*‐value
Age, >65 versus ≤65 (years)	1.10 (0.11, 10.57)	0.935	‐	‐
Gender, female versus male	0.64 (0.09, 4.56)	0.659	‐	‐
BMI, <24 versus ≥24	0.30 (0.03, 2.85)	0.292	‐	‐
EOCG/PS, 1 versus 0	0.00 (0.00, Inf)	0.999	‐	‐
Smoking, no versus yes	0.19 (0.02, 1.81)	0.148	‐	‐
Histology, LUAD versus LUSC	1.15 (0.12, 11.07)	0.903	‐	‐
Type of surgery, lobectomy versus pneumonectomy	1.15 (0.12, 11.07)	0.903	‐	‐
Covid‐19, infection versus non‐infection	16.34 (1.68, 158.53)	**0.016**	21.62 (1.58, 296.06)	**0.021**
Stage, III–IV versus I–II	17.96 (1.86, 173.28)	**0.013**	23.01 (1.76, 300.30)	**0.017**

Abbreviations: BMI, body mass index; HR, hazard ratio; LUAD, lung adenocarcinoma; LUSC, lung squamous cell carcinoma; PFS, progression‐free survival. Bold values indiacates *P* < 0.05.

**FIGURE 2:**
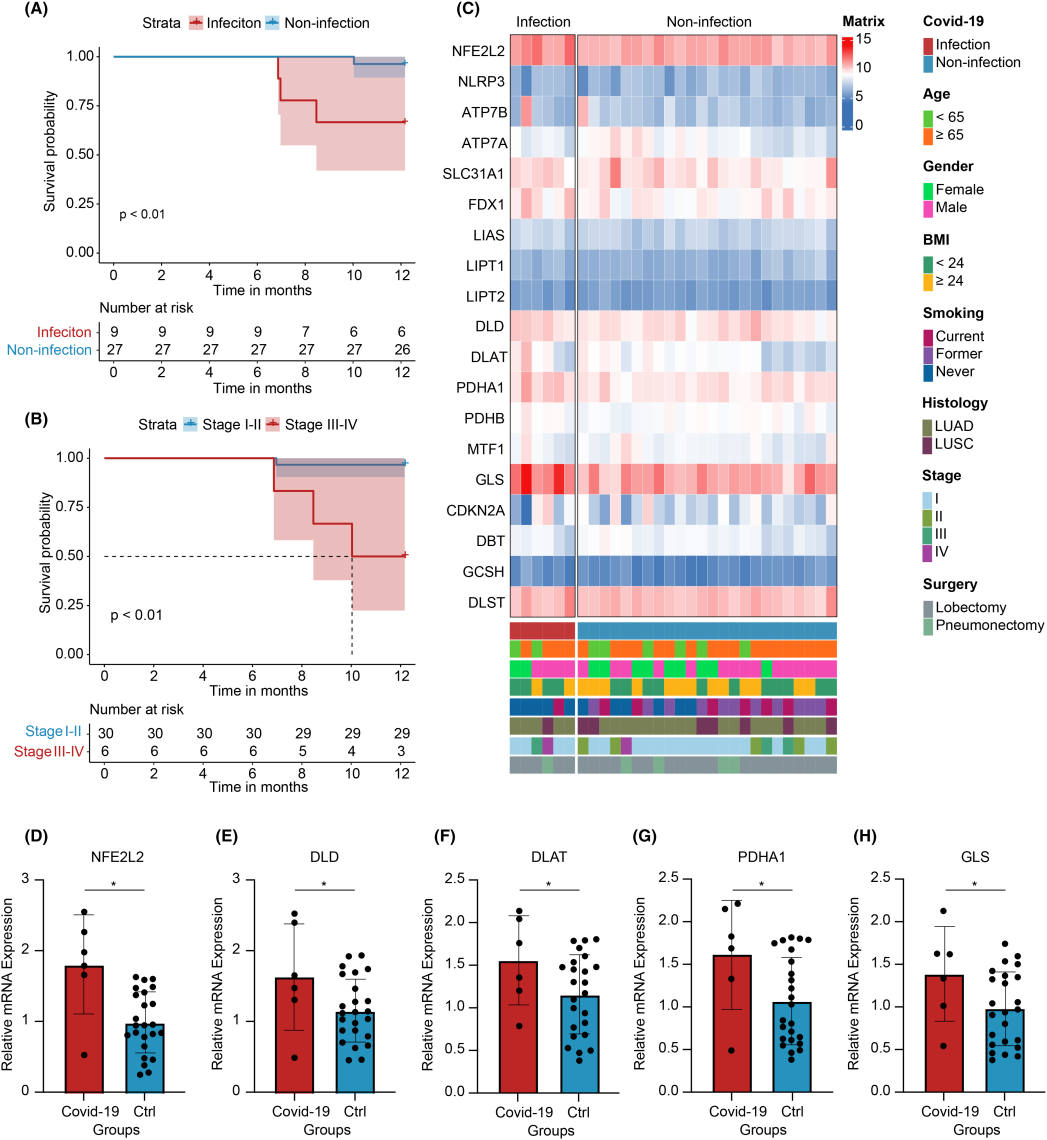
Progression curves and expression heatmap of multi‐centre lung cancer samples. (A) Survival curves for NSCLC patients with COVID‐19 infection and control group without progression. (B) Survival curves for NSCLC patients with stage III–IV and stage I–II group without progression. (C) Integrated heatmap of cancer tissue and clinical information from 30 NSCLC patients. (D–H) Bar charts of mRNA expression levels of NFE2L2, DLD, DLAT, PDHA1 and GLS genes. *P < 0.05.

In Figure 2A, Kaplan–Meier curves demonstrated a significant increase in cumulative progression incidence among NSCLC patients with COVID‐19 infection compared to those without infection (log‐rank *p* < 0.01). Similarly, Figure 2B showed a notable elevation in cumulative progression incidence in patients at stage III‐IV (log‐rank *p* < 0.01). Additionally, RNAseq data of the patients were analysed, and a heatmap (Figure 2C) was generated to illustrate the expression levels of Cuproptosis‐related genes. The results revealed an overall upregulation of Cuproptosis‐related gene expression in lung cancer patients with COVID‐19 infection. We conducted qPCR to measure the expression levels of cuproptosis‐related genes, including NFE2L2, DLD, DLAT, PDHA1 and GLS. Our results revealed that the expression levels of these genes were consistently higher in the COVID‐19 group compared to the control group (Figure 2D–H).

**FIGURE 3:**
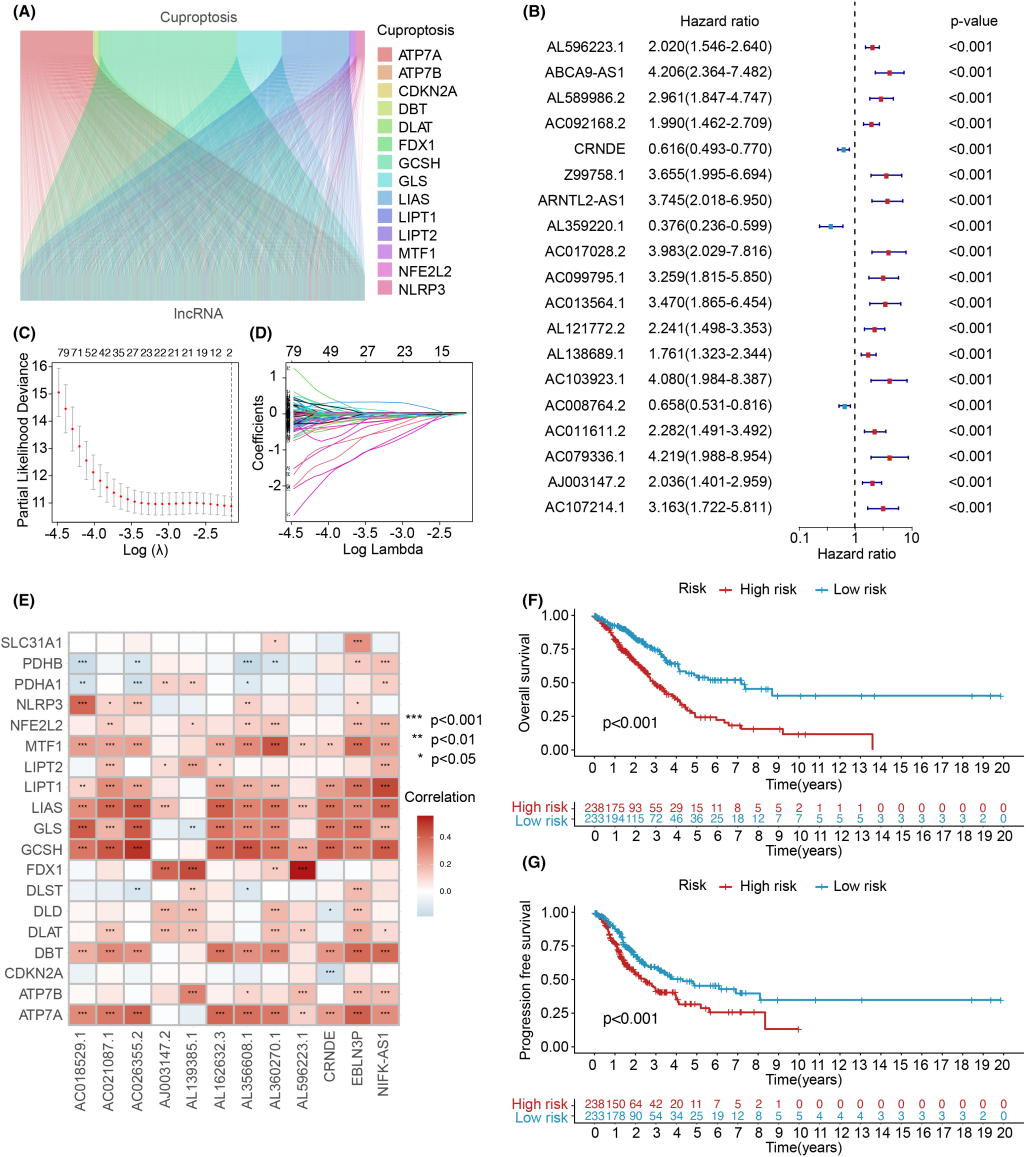
Screening and prognostic analysis of cuproptosis‐associated lncRNAs. (A) Sankey diagram illustrating the coexpression between 19 cuproptosis‐related genes and 2339 cuproptosis‐related lncRNAs. (B) Univariate Cox regression analysis to identify the prognostic cuproptosis‐related lncRNAs. (C and D). Lasso–Cox regression analysis performed to construct prognostic prediction models. (E) Correlation analysis of the 19 cuproptosis‐related genes with the 8 prognostic cuproptosis‐related lncRNAs. (F) Kaplan–Meier curves depicting survival analysis in the high‐ and low‐risk groups. (G) Kaplan–Meier curves depicting progression analysis in the high‐ and low‐risk groups. Statistical significance denoted as *p < 0.05, **p < 0.01 and ***p < 0.001.

To investigate lncRNAs related to cuproptosis, we filtered out protein‐coding genes in the TCGA_LUAD dataset obtained from the ‘TCGA’ database. A total of 19 cuproptosis‐related genes were collected, and through Pearson correlation analysis, we identified 2238 cuproptosis‐related lncRNAs. The relationship between cuproptosis‐related genes and cuproptosis‐related lncRNAs was visualized using a Sankey plot (Figure 3A). Subsequently, univariate Cox regression analysis was performed to assess the prognostic significance of cuproptosis‐related lncRNAs (*p* < 0.05).

For further analysis, the cohort of 501 patients was divided into a training group (*n* = 244) and a validation group (*n* = 243). The clinical characteristics of LUAD patients are presented in Table 3. Notably, there were no significant differences observed in clinical traits between the training and validation groups.

**TABLE 3:** The clinical basic information of TCGA‐LUAD.

Covariates	Type	Total	Test	Train	*p* value
Age	≤65	225 (47.77%)	113 (48.09%)	112 (47.46%)	0.8915
	>65	236 (50.11%)	116 (49.36%)	120 (50.85%)	
	Unknown	10 (2.12%)	6 (2.55%)	4 (1.69%)	
Gender	Female	256 (54.35%)	132 (56.17%)	124 (52.54%)	0.4853
	Male	215 (45.65%)	103 (43.83%)	112 (47.46%)	
Stage	Stage I	255 (54.14%)	119 (50.64%)	136 (57.63%)	0.3172
	Stage II	108 (22.93%)	57 (24.26%)	51 (21.61%)	
	Stage III	75 (15.92%)	42 (17.87%)	33 (13.98%)	
	Stage IV	25 (5.31%)	15 (6.38%)	10 (4.24%)	
	Unknown	8 (1.7%)	2 (0.85%)	6 (2.54%)	
T	T1	160 (33.97%)	71 (30.21%)	89 (37.71%)	0.0822
	T2	250 (53.08%)	136 (57.87%)	114 (48.31%)	
	T3	39 (8.28%)	16 (6.81%)	23 (9.75%)	
	T4	19 (4.03%)	9 (3.83%)	10 (4.24%)	
	TX	3 (0.64%)	3 (1.28%)	0 (0%)	
M	M0	318 (67.52%)	158 (67.23%)	160 (67.8%)	0.7161
	M1	24 (5.1%)	14 (5.96%)	10 (4.24%)	
	MX	125 (26.54%)	63 (26.81%)	62 (26.27%)	
	Unknown	4 (0.85%)	0 (0%)	4 (1.69%)	
N	N0	304 (64.54%)	140 (59.57%)	164 (69.49%)	0.0612
	N1	87 (18.47%)	51 (21.7%)	36 (15.25%)	
	N2	66 (14.01%)	34 (14.47%)	32 (13.56%)	
	N3	2 (0.42%)	1 (0.43%)	1 (0.42%)	
	NX	11 (2.34%)	9 (3.83%)	2 (0.85%)	
	Unknown	1 (0.21%)	0 (0%)	1 (0.42%)	

### 3.2 Construction and validation of prognostic markers for cuproptosis‐related long noncoding RNAs

In Figure 3B, the results of univariate Cox analysis for 21 cuproptosis‐related lncRNAs were presented. Subsequently, Lasso–Cox regression was performed to further screen 12 lncRNAs and identify trajectory changes in regression coefficients and cross‐validation results for model construction (Figure 3C,D). Multiple stepwise Cox regression analysis was then conducted, resulting in the selection of eight cuproptosis‐related lncRNAs with significant survival associations, which were used to establish risk score models.

**FIGURE 4:**
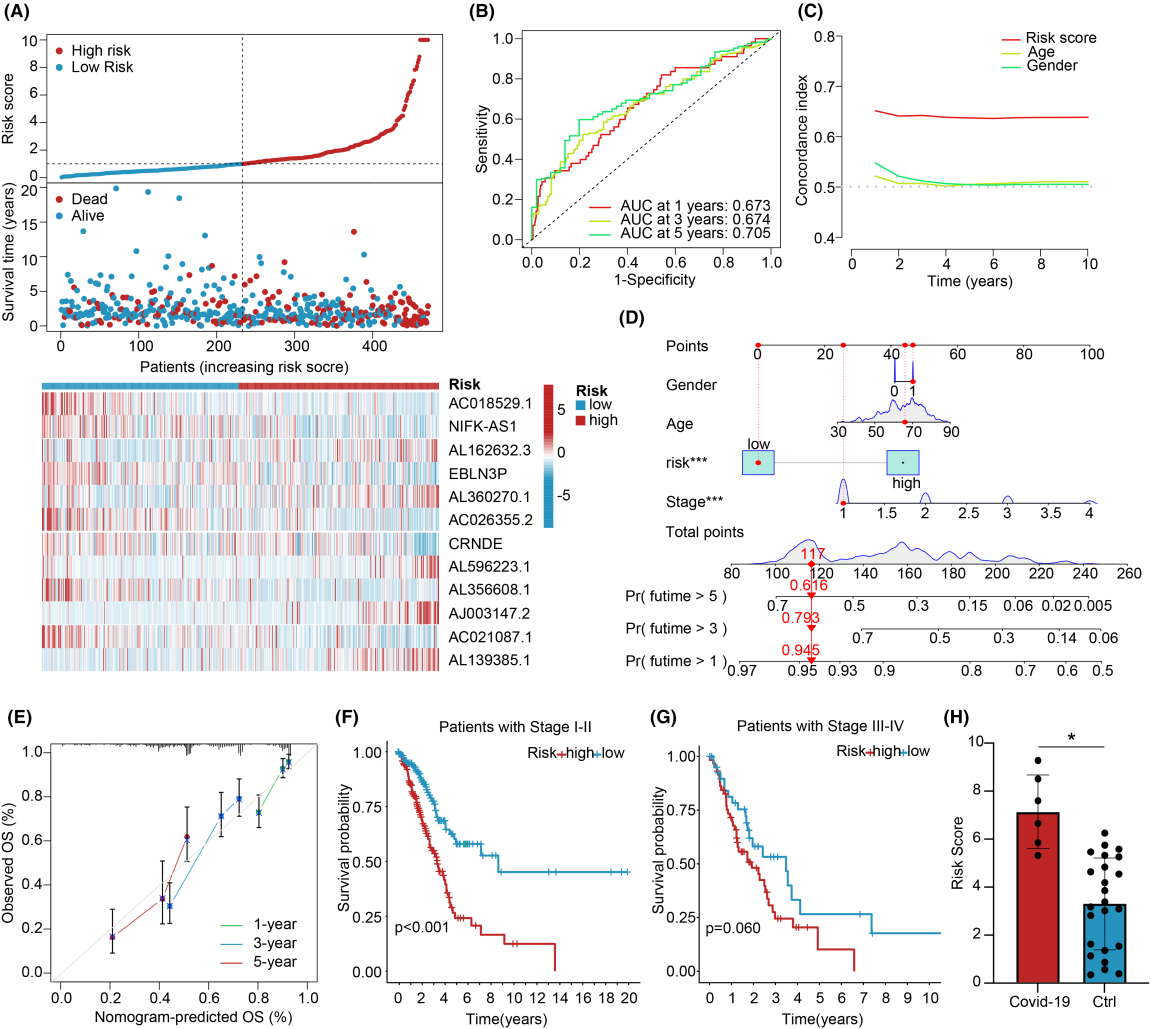
Construction and validation of a prognostic model for cuproptosis‐associated lncRNAs. (A) Distribution of risk scores and survival status in NSCLC patients and heatmap displaying the prognostic markers and overall survival (OS). (B) TimeROC curve predicted the 1, 3 and 5‐year OS for NSCLC patients. (C) C‐index showed that the risk model had superior predictive accuracy compared to other clinical parameters. (D) A nomogram was constructed based on the CRLPM to predict prognosis. (E) Calibration curves were used to predict 1‐, 3‐ and 5‐year OS. (F) Kaplan–Meier curves depicted the OS of patients with stage I–II. G. Kaplan–Meier curves illustrated the OS of patients with stage III–IV. H. Risk scores for patients in the COVID‐19 and non‐COVID‐19 groups. *P < 0.05.

To explore the relationship between cuproptosis‐related genes and the selected CRLPMs, a heatmap (Figure 3E) was generated. Using the median risk score, patients from the training group were divided into high‐ and low‐risk groups for survival analysis. Kaplan–Meier analysis demonstrated that patients in the high‐risk group had significantly poorer OS compared to those in the low‐risk group (*p* < 0.05). Furthermore, significant differences in OS and PFS between the high‐ and low‐risk groups were observed in the validation and entire cohorts (*p* < 0.05; Figures 3F,G and S1A,B). The distribution of risk scores and survival status of patients were visualized, while a heatmap depicted the expression levels of the 12 cuproptosis‐related lncRNAs in the high‐ and low‐risk groups (Figure 4A).

### 3.3 Independence of cuproptosis‐related lncRNAs as prognostic markers in predicting overall survival

**FIGURE 5:**
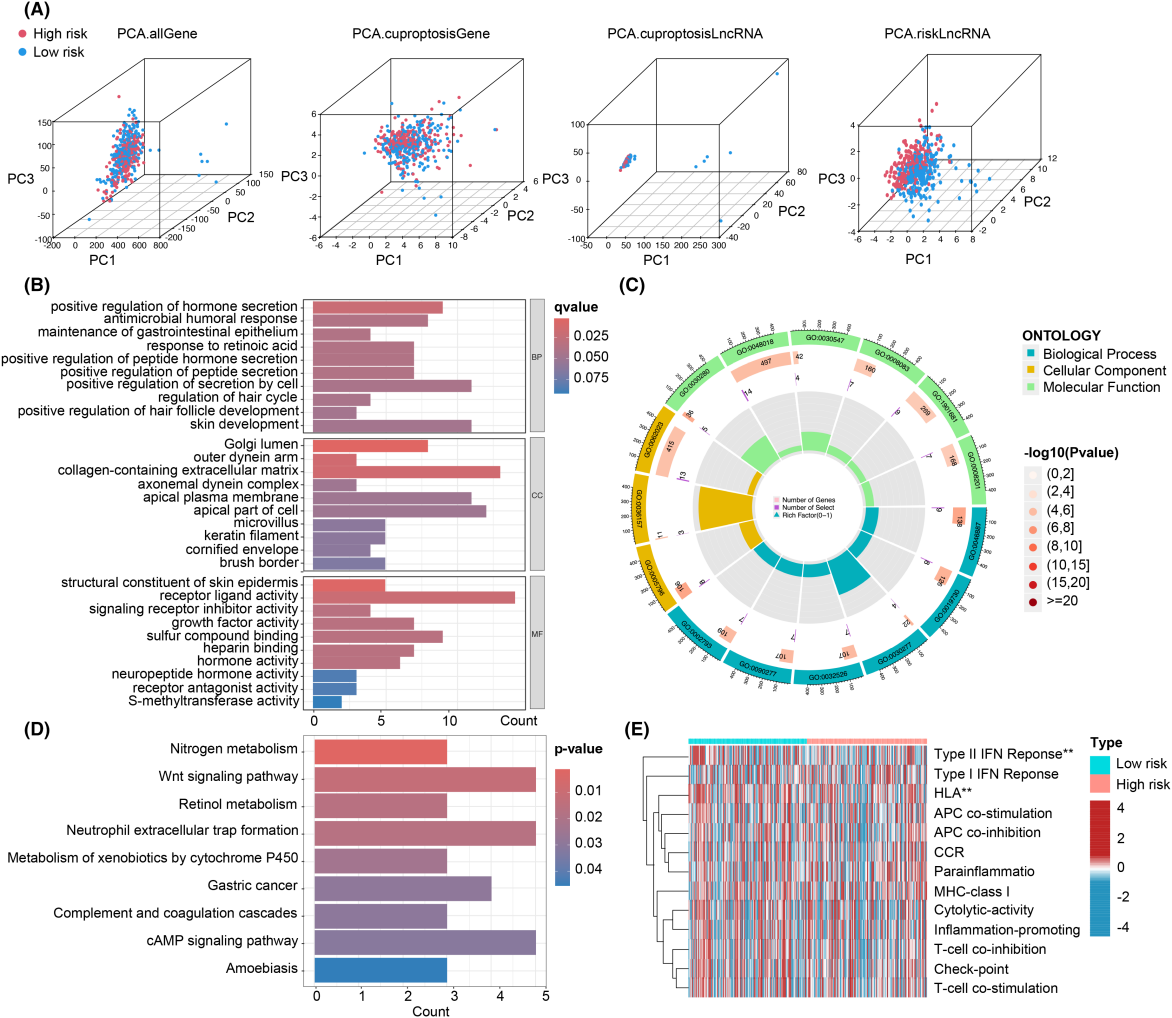
Gene Ontology (GO) and Kyoto Encyclopedia of Genes and Genomes (KEGG) pathway enrichment analysis. (A) Principal component analysis (PCA) comparing the high‐ and low‐risk groups based on all genes, cuproptosis‐related genes, cuproptosis‐related long noncoding RNAs (lncRNAs) and cuproptosis‐related lncRNA prognostic markers. (B) Barplot displaying the top 10 enriched GO terms. (C) Circle diagram presenting the results of GO enrichment analysis. (D) Barplot illustrating the top 30 enriched KEGG terms. (E) Heatmap illustrating the distribution of tumour‐infiltrating lymphocytes based on single‐sample gene set enrichment analysis (ssGSEA) algorithms in the high‐ and low‐risk groups of NSCLC. Statistical significance indicated as *p < 0.05, **p < 0.01 and ***p < 0.001.

Univariate and multivariate Cox regression analyses were performed to assess the predictive value of the prognostic model. In the univariate Cox analysis, age, gender, stage and risk score exhibited statistically significant differences (Figure S2A). These variables retained their prognostic value for PFS in the multivariate Cox regression analysis (Figure S2B). ROC curves were generated to evaluate the accuracy and diagnostic value of cuproptosis‐related lncRNAs in predicting OS, yielding AUC values of 0.673 at 1 year, 0.674 at 3 years and 0.705 at 5 years (Figure 4B). Both the C‐index and ROC curve indicated that the prognostic model demonstrated superior predictive accuracy compared to other clinical factors, including age, gender, grade and stage (Figure 4C). To provide quantitative predictions of clinical outcomes in patients with LUAD, a prognostic nomogram was constructed based on the risk score and other clinical characteristics (Figure 4D). Calibration plots depicted good agreement between the predicted and observed outcomes of the nomogram (Figure 4E). The clinical relevance of the Cuproptosis‐Related CRLPM was assessed by examining its association with clinical features. The results revealed significant differences in risk score distribution across different clinical stages, particularly in stages I‐II (*p* < 0.001) and III‐IV (*p* = 0.06) (Figure 4F,G). We calculated the risk scores for patients in the COVID‐19 and non‐COVID‐19 groups and found that the COVID‐19 group had significantly higher risk scores (*p* < 0.05) (Figure 4H). PCA displayed distinct separation between the high‐ and low‐risk groups, effectively stratifying LUAD patients based on the risk model of cuproptosis‐related lncRNAs (Figure 5A).

### 3.4 Pathway enrichment analysis and genomic enrichment analysis

To explore the biological functions and pathways related to the DEGs between the high‐ and low‐risk groups, we performed GO and KEGG enrichment analyses. A total of 346 DEGs were identified. In terms of biological processes, the DEGs were mainly enriched in humoral immune response, positive regulation of hormone secretion and positive regulation of secretion by cell based on somatic recombination of immune receptors. In the cellular component category, enrichment was observed in the apical plasma membrane, cornified envelope and collagen‐containing extracellular matrix. Regarding molecular function, the DEGs showed enrichment in hormone activity, growth factor activity and receptor ligand activity (Figure 5B,C). KEGG analysis revealed associations of cuproptosis‐related lncRNAs with the Wnt signalling pathway, neutrophil extracellular trap formation and cAMP signalling pathway (Figure 5D). We conducted a comparative analysis of the pathways listed above between high‐risk and low‐risk groups, revealing significant differences in the Type II IFN Response pathway (Figure 5E).

### 3.5 Prediction of intratumoral immune cell infiltration and immunotherapy response

**FIGURE 6:**
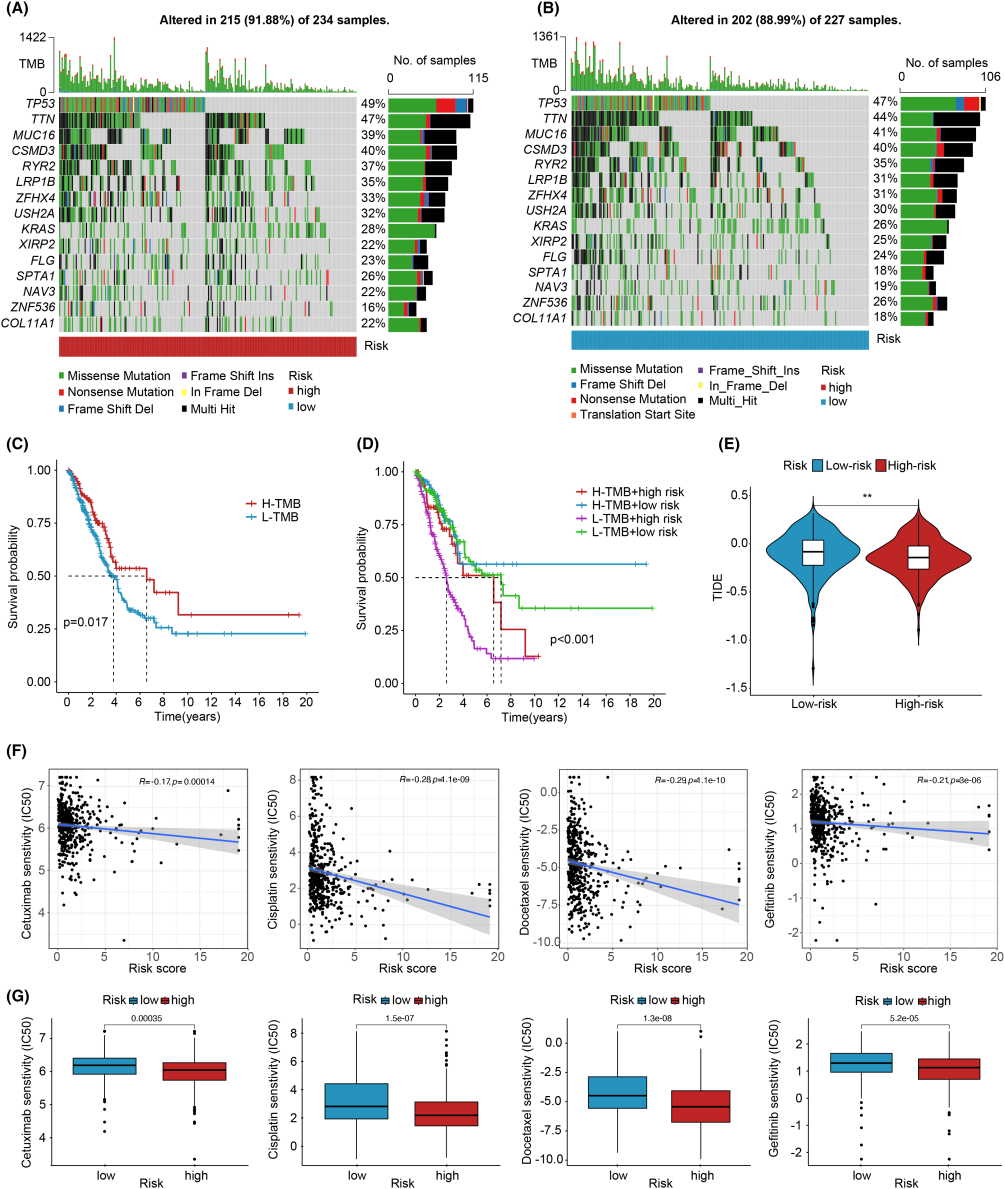
Mutation burden of cuproptosis‐associated lncRNAs and analysis of immune and drug sensitivity. (A) Waterfall plot displaying the top 15 mutant genes in the high‐risk group of NSCLC. (B) Waterfall plot depicting the top 15 mutant genes in the low‐risk group of NSCLC. (C) Survival analysis curves comparing the high‐ and low‐TMB groups. (D) Combined survival curve considering both TMB and risk score in NSCLC. (E) Comparison of TIDE prediction scores between the high‐ and low‐risk groups. (F) Correlation analysis of drug IC_50_ and risk scores. (G) Drug sensitivity (IC_50_) of high‐ and low‐risk patients in NSCLC. **p < 0.01.

In Figure 6A, a heatmap was presented to illustrate the immune response based on the ssGSEA algorithm. By applying ssGSEA to TCGA‐LUAD data and correlating immune cell populations with related functions, significant differences were observed in T cell functions, including regulation of inflammation, HLA, checkpoint inhibition and costimulation and coinhibition, between the high‐ and low‐risk groups. These findings suggest an association between the CRLPM and immune cell infiltration in NSCLC. Additionally, using the TIDE algorithm, we predicted the potential efficacy of immunotherapy for patients. Figure 6E demonstrates significant differences in TIDE scores between the high‐ and low‐risk groups, with lower TIDE scores observed in the high‐risk group. This supports the notion that patients in the high‐risk group have a lower likelihood of immune escape and may potentially benefit more from immunotherapy.

### 3.6 TMB and prognostic marker of cuproptosis‐related lncRNAs

To investigate the potential role of TMB in NSCLC, we collected somatic mutation data from NSCLC samples and calculated corresponding TMB scores. As shown in Figure S2C, there was no significant increase in TMB scores observed in the high‐risk group compared to the low‐risk group. We further categorized patients into ‘High‐TMB’ and ‘Low‐TMB’ groups using median cutoff points and conducted survival analyses. The results demonstrated that the high‐risk group had a lower survival rate than the low‐risk group in NSCLC (Figure 6C). Additionally, a combined survival analysis incorporating tumour mutation load and risk scores showed significant effects on OS in NSCLC patients (Figure 6D). Furthermore, we compared the mutation landscapes between the high‐ and low‐risk groups of the CRLPM. Waterfall plots were generated to visualize the 15 genes with the highest mutation frequency in each group. The findings indicated a higher frequency of mutation events in the high‐risk group (Figure 6A,B), with TP53 being the gene most frequently mutated.

### 3.7 Drug sensitivity analysis and individualized treatment potential of CRLPM in NSCLC

To assess the potential of the CRLPM for personalized treatment in NSCLC, we investigated the relationship between risk scores and the half‐maximal inhibitory concentration (IC_50_) of drugs used in NSCLC treatment. We compared the sensitivity of 257 common anticancer drugs between the high‐ and low‐risk groups. As shown in Figure 6F,G, the sensitivity of 4 out of the 257 anticancer drugs differed significantly between the high‐ and low‐risk groups (*p* < 0.05). Notably, cetuximab, cisplatin, docetaxel and gefitinib exhibited lower IC_50_ values in the high‐risk group, indicating a higher sensitivity to these drugs. This suggests that these drugs may have potential therapeutic roles in NSCLC. Furthermore, the results revealed an inverse association between risk scores and IC_50_ values for most drugs in NSCLC patients. The IC_50_ integration and correlation plot of other potential effective drugs have been stored in Data S1 and have been uploaded.

## 4 DISCUSSION

This multicentre study included the enrollment of 36 patients diagnosed with NSCLC from three different centres. Surgical tissue samples were collected, and successful RNA extraction was performed. Subsequently, RNA‐seq analysis was conducted on a subset of 30 patients to investigate factors associated with cuproptosis. Clinical analysis revealed that NSCLC patients with COVID‐19 infection had a poorer prognosis, accompanied by elevated expression levels of cuproptosis‐related genes. To validate our findings, data from the TCGA database were utilized, and additional analyses regarding immunotherapy response and drug sensitivity were performed. The results demonstrated that the high‐risk group, characterized by elevated cuproptosis‐related lncRNA scores, exhibited lower OS and PFS compared to the control group. Utilizing the TIDE database, it was found that immune‐related treatments showed greater efficacy in the high‐risk group. Furthermore, drug sensitivity testing revealed that cetuximab, cisplatin, docetaxel and gefitinib exhibited promising treatment effects in the high‐risk group.

In our study, we observed significant enrichment of GO terms and KEGG pathways associated with cuproptosis and related biological processes.

Specifically, the GO analysis revealed enrichment in categories related to oxidative stress, cell death and immune response, suggesting the involvement of DEGs in modulating these crucial pathways. These findings imply a potential role of DEGs in regulating cuproptosis, a form of regulated cell death mediated by copper ions. Similarly, the KEGG pathway analysis identified enrichment in pathways associated with inflammation, metabolism and cell signalling. This suggests that DEGs may influence cuproptosis and related lncRNAs through intricate regulatory networks involved in these pathways.Overall, the enrichment analysis highlights the multifaceted impact of DEGs on cuproptosis and related lncRNAs, providing valuable insights into the molecular mechanisms underlying their dysregulation in NSCLC patients, particularly those affected by COVID‐19 infection. These findings contribute to our understanding of the pathogenesis of NSCLC and may have implications for the development of novel therapeutic strategies targeting cuproptosis and its associated pathways.

Alterations in immune cell phenotypes have been observed in both lung cancer patients and individuals with COVID‐19, suggesting potential modifications to the immune system. Tian et al.^16^ reported reductions in helper T cells (Th) and regulatory T cells (Treg) in COVID‐19 patients, which were more pronounced in severe cases. Furthermore, the expression of inhibitory receptors such as CTLA‐4, PD‐1, TIGIT, TIM‐3 and NKG2A on CD8+ T cells was found to increase during early infection. Notably, genes like PD‐1 and TIM‐3 have also been implicated in lung cancer treatment.^17^ The severity of COVID‐19 disease has been associated with the magnitude of the antibody response against SARS‐CoV‐2. Convalescent COVID‐19 patients were shown to maintain specific T‐cell memory against SARS‐CoV‐2 for up to 10 months.^18^, ^19^ However, the underlying mechanisms of immune cell activation in COVID‐19 are still not completely understood, and these factors can impact the biology of lung cancer cells.

Copper is an essential trace element in the human body and plays a critical role in various biological systems. It is involved in copper homeostasis, which regulates copper metabolism to maintain a dynamic balance. Disruptions in copper homeostasis have been associated with the development of diseases such as Wilson's disease, Menkes disease, Alzheimer's disease, Parkinson's disease, obesity, hypertension and respiratory system tumours.^20^, ^21^, ^22^, ^23^ Copper also plays a role in immune cell functioning, including natural killer (NK) cells and macrophages, and exhibits antiviral properties against certain viruses, including bronchiolitis viruses, single‐stranded or double‐stranded DNA and RNA viruses.^24^ Interestingly, copper has also been implicated in COVID‐19. Studies have reported higher levels of copper in the whole blood of severe COVID‐19 patients compared to non‐severe cases.^25^ Additionally, serum copper ion levels increase during the early stages of the disease due to inflammatory responses.^26^ Serum copper and selenium levels have shown promise in predicting patient outcomes in both lung cancer and COVID‐19. Furthermore, supplementation with copper ions may improve prognosis in individuals diagnosed with copper deficiency.^27^ While copper intoxication, specifically cuproptosis induced by copper ions, has been identified as a potential therapeutic target for Wilson's disease and cancer, its role in lung cancer combined with COVID‐19 remains unclear.^28^

Our study provides evidence that lung cancer patients with concomitant COVID‐19 infection experience a worse prognosis, which is associated with cuproptosis activity. Immune scoring analysis revealed significant upregulation of the Type II IFN Response and HLA‐related pathways in the high‐risk group characterized by cuproptosis‐related lncRNAs. These biological processes exhibited correlations with macrophages, regulatory T cells (Treg) and CD8+ T cells.

Macrophages can be divided into two main subtypes: M1 macrophages with pro‐inflammatory effects and M2 macrophages with anti‐inflammatory and immune regulatory functions. M2 macrophages contribute to tissue repair, angiogenesis and maintaining homeostasis by producing IL‐10 and TGF‐β, which help reduce inflammatory responses.^29^ Neutrophils are the most abundant immune cells in the body, and previous studies have suggested a potential association between elevated neutrophil levels and unfavourable prognosis in tumours.^30^, ^31^, ^32^ This correlation may be attributed to various factors, including the immunosuppressive properties of neutrophils, their ability to promote tumour growth and their facilitation of tumour cell migration and invasion through the release of hepatocyte growth factor (HGF) and other molecules.^32^ In certain cancers such as renal cell carcinoma and bronchioloalveolar carcinoma, intratumoral neutrophils contribute to immune suppression and enhanced tumour growth through mechanisms involving myeloid‐derived suppressor cells, arginase, reactive oxygen species, B7‐Hx, PD‐1 and HGF.^30^, ^31^ Similarly, in bronchioloalveolar carcinoma, tumour‐infiltrating neutrophils produce HGF, which interacts with c‐met receptors on tumour cells to enhance tumour cell migration. Elevated levels of HGF in bronchoalveolar lavage fluid are associated with poorer clinical outcomes in bronchioloalveolar carcinoma patients.^30^ T follicular helper cells play a crucial role in supporting B cells to differentiate into antibody‐producing plasma cells, thereby aiding in viral and bacterial clearance. Numerous studies have demonstrated the importance of T follicular helper cells in controlling infections caused by hepatitis C virus, human immunodeficiency virus and Group A Streptococcus.^33^ CD8+ T cells function as cytotoxic cells and the presence of virus‐specific CD8+ lymphocytes is associated with better outcomes in SARS‐CoV‐2 infection.^34^ NK cells are early effector lymphocytes, and lower NK cell counts have been linked to reduced survival rates in COVID‐19.^35^

Based on our drug sensitivity testing, we observed positive treatment outcomes in the high‐risk group of cuproptosis‐related lncRNAs when treated with cetuximab. Cetuximab is an IgG1 monoclonal antibody that specifically targets the extracellular domain of the epidermal growth factor receptor, thereby blocking the interaction between the receptor and its ligands and inhibiting downstream signalling pathways.^36^ Besides its direct inhibitory effects, cetuximab also exhibits immune‐mediated cytotoxicity through mechanisms such as antibody‐dependent cell‐mediated cytotoxicity.^37^ This unique mode of action distinguishes cetuximab from tyrosine kinase inhibitors. In advanced NSCLC, combination therapy involving cetuximab and various platinum‐based regimens has been extensively evaluated and has shown favourable outcomes in diverse patient populations.^38^ Our study provides novel evidence supporting the potential use of cetuximab in the treatment of lung cancer.

Our study investigated the prognostic value and therapeutic implications of cuproptosis‐associated lncRNAs in NSCLC, specifically focusing on patients co‐infected with COVID‐19. Similar to the findings by Hou et al.,^39^ who developed a signature based on cuproptosis‐related lncRNAs for lung squamous cell carcinoma (LUSC), our research identified significant lncRNAs linked to cuproptosis that impact patient prognosis. Hou et al. highlighted three lncRNAs (AC002467.1, LINC01740 and LINC02345) with strong predictive capabilities for LUSC prognosis and potential benefits for immunotherapy. In parallel, our study discovered that MAMDC2‐AS1 is a crucial cuproptosis‐associated lncRNA in NSCLC, particularly in the context of COVID‐19 infection, and demonstrated its significant prognostic value. Both studies underscore the importance of cuproptosis‐related lncRNAs in cancer prognosis and suggest that these lncRNAs could be leveraged to improve therapeutic strategies, including immunotherapy. While Hou et al. focused on LUSC, our findings extend the relevance of cuproptosis‐associated lncRNAs to NSCLC, offering new insights and potential avenues for treatment in a broader spectrum of lung cancers. Further research with larger cohorts and balanced control groups is warranted to validate these findings and fully harness the therapeutic potential of cuproptosis‐associated lncRNAs in lung cancer.

We acknowledge the limitation of having a small control group size (*N* = 7) in our study. This discrepancy in sample size between the control group and the COVID‐19 positive NSCLC patients introduces potential biases and may affect the robustness of our findings. Specifically, the small control group size can lead to skewed results and reduced statistical power, making it challenging to draw definitive conclusions. To mitigate these biases, we have employed rigorous statistical methods and sensitivity analyses. However, we recommend that future studies include larger and more balanced sample sizes to validate our findings and provide more comprehensive insights into the impact of COVID‐19 on NSCLC patients.

## 5 CONCLUSION

In conclusion, our multicentre study investigating NSCLC patients revealed that the presence of COVID‐19 infection was associated with a poorer prognosis and increased expression of cuproptosis‐related genes. Through RNA‐seq analysis and utilizing data from the TCGA database, we identified a high‐risk group characterized by elevated cuproptosis‐related lncRNA scores, which exhibited lower OS and PFS rates. Immune‐related treatments demonstrated greater efficacy in this high‐risk group, as supported by analyses using the TIDE database. Furthermore, drug sensitivity testing highlighted the potential therapeutic benefits of cetuximab, cisplatin, docetaxel and gefitinib. These findings provide valuable insights into the role of cuproptosis in NSCLC and offer opportunities for further exploration and potential therapeutic interventions.

## AUTHOR CONTRIBUTIONS

**Jing Li:** Conceptualization (equal); data curation (equal); methodology (equal); software (equal); writing – original draft (equal). **Nan Wang:** Data curation (equal); formal analysis (equal); software (equal); validation (equal). **Guocai Mao:** Formal analysis (equal); funding acquisition (equal); methodology (equal); validation (equal). **Jiantang Wang:** Formal analysis (equal); investigation (equal); software (equal). **Mengqi Xiang:** Formal analysis (equal); validation (equal); visualization (equal). **Huachuan Zhang:** Formal analysis (equal); validation (equal); visualization (equal). **Daxiong Zeng:** Supervision (equal). **Haitao Ma:** Supervision (equal); visualization (equal). **Junhong Jiang:** Funding acquisition (equal); investigation (equal); software (equal); supervision (equal); visualization (equal).

## FUNDING INFORMATION

This study was funded by the Jiangsu Province Special Program of Medical Science (no. BE2016672) and the Suzhou Science and Technology Planning Project (nos. SLT201917 and SKY2021026).

## CONFLICT OF INTEREST STATEMENT

There are no conflicts of interest to disclose.

## CONSENT FOR PUBLICATION

We obtained consent from all authors for publication.

## Supporting information


Figure S1.

Data S1.

## Data Availability

The datasets used and/or analyzed during the current study are available from the corresponding author on reasonable request.

## References

[1] Allemani C , Matsuda T , Di Carlo V , et al. Global surveillance of trends in cancer survival 2000–14 (CONCORD‐3): analysis of individual records for 37 513 025 patients diagnosed with one of 18 cancers from 322 population‐based registries in 71 countries. Lancet. 2018;391(10125):1023‐1075. doi:10.1016/S0140-6736(17)33326-3 29395269 PMC5879496

[2] Wang S , Sun T , Sun H , et al. Survival improvement in patients with non‐small cell lung cancer between 1983 and 2012: analysis of the Surveillance, Epidemiology, and End Results database. Tumour Biol. 2017;39(5):1010428317691677. doi:10.1177/1010428317691677 28459218

[3] Horn L , Garassino M . COVID‐19 in patients with cancer: managing a pandemic within a pandemic. Nat Rev Clin Oncol. 2021;18(1):1‐2. doi:10.1038/s41571-020-00441-5 33060841 PMC7557307

[4] Passaro A , Peters S , Mok TSK , Attili I , Mitsudomi T , de Marinis F . Testing for COVID‐19 in lung cancer patients. Ann Oncol. 2020;31(7):832‐834. doi:10.1016/j.annonc.2020.04.002 32278879 PMC7144604

[5] Liang W , Guan W , Chen R , et al. Cancer patients in SARS‐CoV‐2 infection: a nationwide analysis in China. Lancet Oncol. 2020;21(3):335‐337. doi:10.1016/S1470-2045(20)30096-6 32066541 PMC7159000

[6] Yu J , Ouyang W , Chua MLK , Xie C . SARS‐CoV‐2 transmission in patients with cancer at a Tertiary Care Hospital in Wuhan, China. JAMA Oncologia. 2020;6(7):1108‐1110. doi:10.1001/jamaoncol.2020.0980 PMC7097836 32211820

[7] Bakouny Z , Hawley JE , Choueiri TK , et al. COVID‐19 and cancer: current challenges and perspectives. Cancer Cell. 2020;38(5):629‐646. doi:10.1016/j.ccell.2020.09.018 33049215 PMC7528740

[8] Milette S , Fiset PO , Walsh LA , Spicer JD , Quail DF . The innate immune architecture of lung tumors and its implication in disease progression. J Pathol. 2019;247(5):589‐605. doi:10.1002/path.5241 30680732

[9] Tsvetkov P , Coy S , Petrova B , et al. Copper induces cell death by targeting lipoylated TCA cycle proteins. Science. 2022;375(6586):1254‐1261. doi:10.1126/science.abf0529 35298263 PMC9273333

[10] Luo H , Yan J , Zhang D , Zhou X . Identification of cuproptosis‐related molecular subtypes and a novel predictive model of COVID‐19 based on machine learning. Front Immunol. 2023;14:1152223. doi:10.3389/fimmu.2023.1152223 37533853 PMC10393044

[11] Gao N , Li Y , Li J , et al. Long non‐coding RNAs: the regulatory mechanisms, research strategies, and future directions in cancers. Front Oncol. 2020;10:598817. doi:10.3389/fonc.2020.598817 33392092 PMC7775490

[12] Statello L , Guo CJ , Chen LL , Huarte M . Gene regulation by long non‐coding RNAs and its biological functions. Nat Rev Mol Cell Biol. 2021;22(2):96‐118. doi:10.1038/s41580-020-00315-9 33353982 PMC7754182

[13] Iasonos A , Schrag D , Raj GV , Panageas KS . How to build and interpret a nomogram for cancer prognosis. J Clin Oncol. 2008;26(8):1364‐1370. doi:10.1200/JCO.2007.12.9791 18323559

[14] Jiang P , Gu S , Pan D , et al. Signatures of T cell dysfunction and exclusion predict cancer immunotherapy response. Nat Med. 2018;24(10):1550‐1558. doi:10.1038/s41591-018-0136-1 30127393 PMC6487502

[15] Mayakonda A , Lin DC , Assenov Y , Plass C , Koeffler HP . Maftools: efficient and comprehensive analysis of somatic variants in cancer. Genome Res. 2018;28(11):1747‐1756. doi:10.1101/gr.239244.118 30341162 PMC6211645

[16] Qin C , Zhou L , Hu Z , et al. Dysregulation of immune response in patients with coronavirus 2019 (COVID‐19) in Wuhan, China. Clin Infect Dis. 2020;71(15):762‐768. doi:10.1093/cid/ciaa248 32161940 PMC7108125

[17] DiPiazza AT , Graham BS , Ruckwardt TJ . T cell immunity to SARS‐CoV‐2 following natural infection and vaccination. Biochem Biophys Res Commun. 2021;538:211‐217. doi:10.1016/j.bbrc.2020.10.060 33190827 PMC7584424

[18] Jung JH , Rha MS , Sa M , et al. SARS‐CoV‐2‐specific T cell memory is sustained in COVID‐19 convalescent patients for 10 months with successful development of stem cell‐like memory T cells. Nat Commun. 2021;12(1):4043. doi:10.1038/s41467-021-24377-1 34193870 PMC8245549

[19] Roltgen K , Boyd SD . Antibody and B cell responses to SARS‐CoV‐2 infection and vaccination. Cell Host Microbe. 2021;29(7):1063‐1075. doi:10.1016/j.chom.2021.06.009 34174992 PMC8233571

[20] Tumer Z , Moller LB . Menkes disease. Eur J Hum Genet. 2010;18(5):511‐518. doi:10.1038/ejhg.2009.187 19888294 PMC2987322

[21] Bandmann O , Weiss KH , Kaler SG . Wilson's disease and other neurological copper disorders. Lancet Neurol. 2015;14(1):103‐113. doi:10.1016/S1474-4422(14)70190-5 25496901 PMC4336199

[22] Sensi SL , Granzotto A , Siotto M , Squitti R . Copper and zinc dysregulation in Alzheimer's disease. Trends Pharmacol Sci. 2018;39(12):1049‐1063. doi:10.1016/j.tips.2018.10.001 30352697

[23] Ge EJ , Bush AI , Casini A , et al. Connecting copper and cancer: from transition metal signalling to metalloplasia. Nat Rev Cancer. 2022;22(2):102‐113. doi:10.1038/s41568-021-00417-2 34764459 PMC8810673

[24] Raha S , Mallick R , Basak S , Duttaroy AK . Is copper beneficial for COVID‐19 patients? Med Hypotheses. 2020;142:109814. doi:10.1016/j.mehy.2020.109814 32388476 PMC7199671

[25] Zeng HL , Yang Q , Yuan P , Wang X , Cheng L . Associations of essential and toxic metals/metalloids in whole blood with both disease severity and mortality in patients with COVID‐19. FASEB J. 2021;35(3):e21392. doi:10.1096/fj.202002346RR 33577131 PMC7995111

[26] Abdallah B , Seguin C , Aubert E , et al. Past mastering of metal transformation enabled physicians to increase their therapeutic potential. J Trace Elem Med Biol. 2022;71:126926. doi:10.1016/j.jtemb.2022.126926 35033860

[27] Hackler J , Heller RA , Sun Q , et al. Relation of serum copper status to survival in COVID‐19. Nutrients. 2021;13(6):1898. doi:10.3390/nu13061898 PMC8229409 34072977

[28] Chen L , Min J , Wang F . Copper homeostasis and cuproptosis in health and disease. Signal Transduct Target Ther. 2022;7(1):378. doi:10.1038/s41392-022-01229-y 36414625 PMC9681860

[29] Shapouri‐Moghaddam A , Mohammadian S , Vazini H , et al. Macrophage plasticity, polarization, and function in health and disease. J Cell Physiol. 2018;233(9):6425‐6440. doi:10.1002/jcp.26429 PMC13482560 29319160

[30] Wislez M , Rabbe N , Marchal J , et al. Hepatocyte growth factor production by neutrophils infiltrating bronchioloalveolar subtype pulmonary adenocarcinoma: role in tumor progression and death. Cancer Res. 2003;63(6):1405‐1412. 12649206

[31] Jensen HK , Donskov F , Marcussen N , Nordsmark M , Lundbeck F , von der Maase H . Presence of intratumoral neutrophils is an independent prognostic factor in localized renal cell carcinoma. J Clin Oncol. 2009;27(28):4709‐4717. doi:10.1200/JCO.2008.18.9498 19720929

[32] Cupp MA , Cariolou M , Tzoulaki I , Aune D , Evangelou E , Berlanga‐Taylor AJ . Neutrophil to lymphocyte ratio and cancer prognosis: an umbrella review of systematic reviews and meta‐analyses of observational studies. BMC Med. 2020;18(1):360. doi:10.1186/s12916-020-01817-1 33213430 PMC7678319

[33] Dong L , He Y , Cao Y , et al. Functional differentiation and regulation of follicular T helper cells in inflammation and autoimmunity. Immunology. 2021;163(1):19‐32. doi:10.1111/imm.13282 33128768 PMC8044332

[34] Sette A , Crotty S . Adaptive immunity to SARS‐CoV‐2 and COVID‐19. Cell. 2021;184(4):861‐880. doi:10.1016/j.cell.2021.01.007 33497610 PMC7803150

[35] Zheng R , Zhou J , Song B , et al. COVID‐19‐associated coagulopathy: thromboembolism prophylaxis and poor prognosis in ICU. Exp Hematol Oncol. 2021;10(1):6. doi:10.1186/s40164-021-00202-9 33522958 PMC7848868

[36] Goldstein NI , Prewett M , Zuklys K , Rockwell P , Mendelsohn J . Biological efficacy of a chimeric antibody to the epidermal growth factor receptor in a human tumor xenograft model. Clin Cancer Res. 1995;1(11):1311‐1318. 9815926

[37] Kurai J , Chikumi H , Hashimoto K , et al. Antibody‐dependent cellular cytotoxicity mediated by cetuximab against lung cancer cell lines. Clin Cancer Res. 2007;13(5):1552‐1561. doi:10.1158/1078-0432.CCR-06-1726 17332301

[38] Ettinger DS . Emerging profile of cetuximab in non‐small cell lung cancer. Lung Cancer. 2010;68(3):332‐337. doi:10.1016/j.lungcan.2009.07.012 19783064

[39] Hou C , Wu X , Li C , Wang C , Liu J , Luo Q . A cuproptosis‐associated long non‐coding RNA signature for the prognosis and immunotherapy of lung squamous cell carcinoma. Biomol Biomed. 2023;23(4):624‐633. doi:10.17305/bb.2022.8481 36724022 PMC10351099

