# Supplementary figures and images for: Cuproptosis‐associated lncRNA impact prognosis in patients with non‐small cell lung cancer co‐infected with COVID‐19
Source: J Cell Mol Med. 2024 Sep 3;28(17):e70059. doi: 10.1111/jcmm.70059 (PMC11371660; doi:10.1111/jcmm.70059)

Figure S1

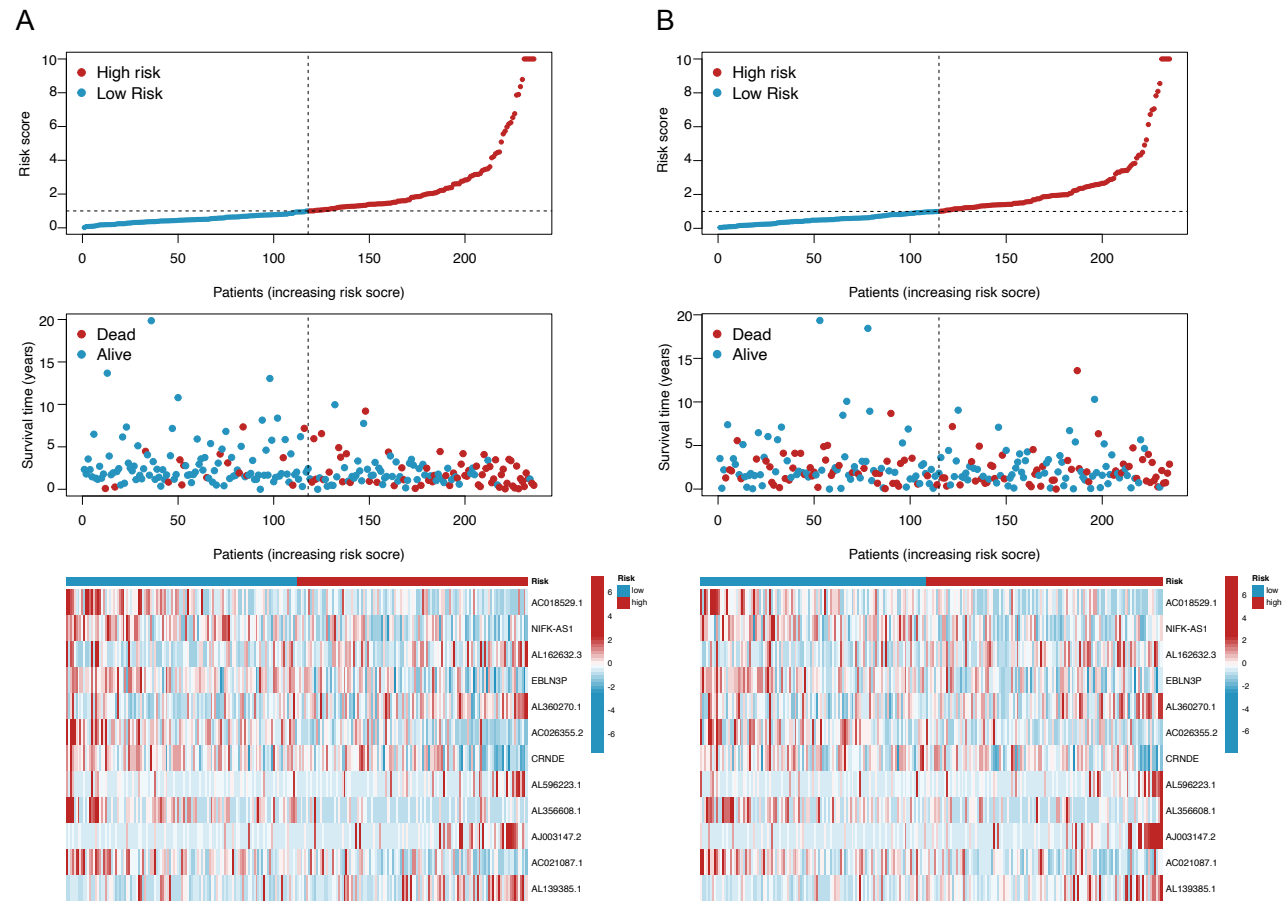

Figure S2

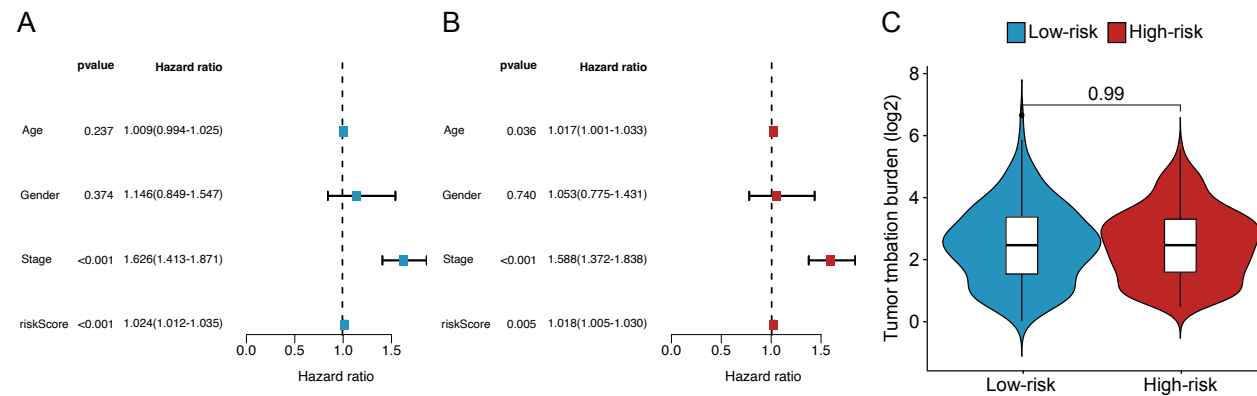

Supplement: Supplementary file 1 — Figure S1. [file JCMM-28-e70059-s001.pdf]

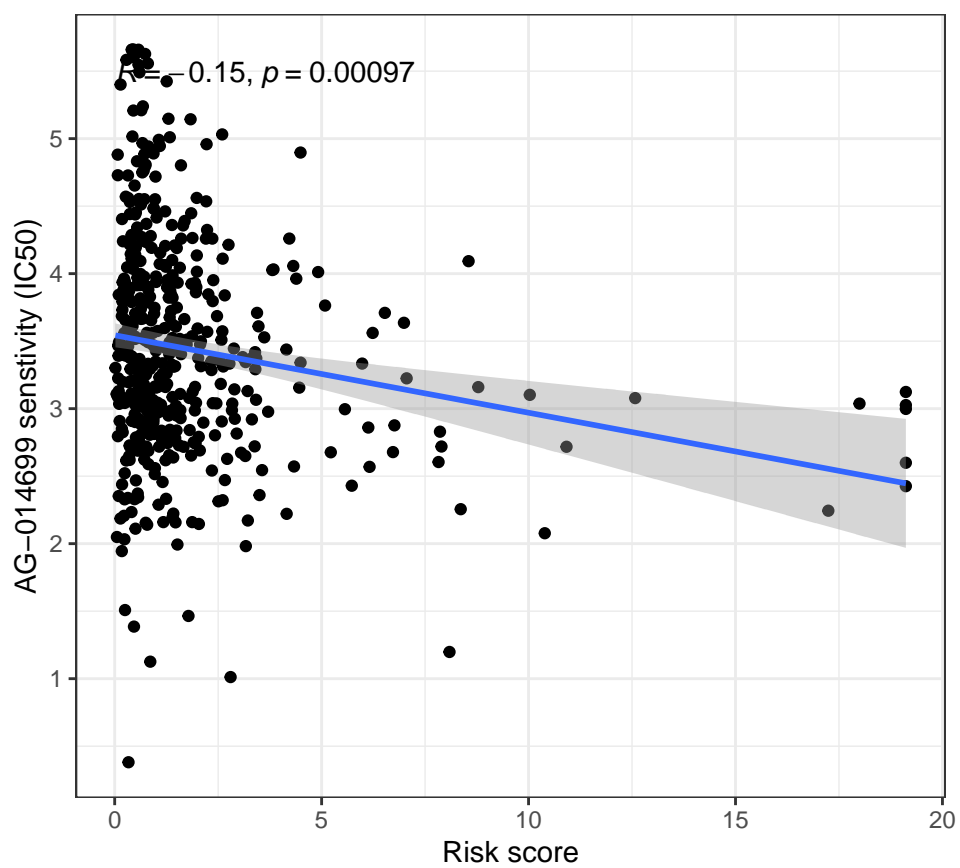

Supplement: Supplementary file 2 — Data S1. [file JCMM-28-e70059-s002.zip › Supplemental Material II/Cor.AG-014699.pdf]

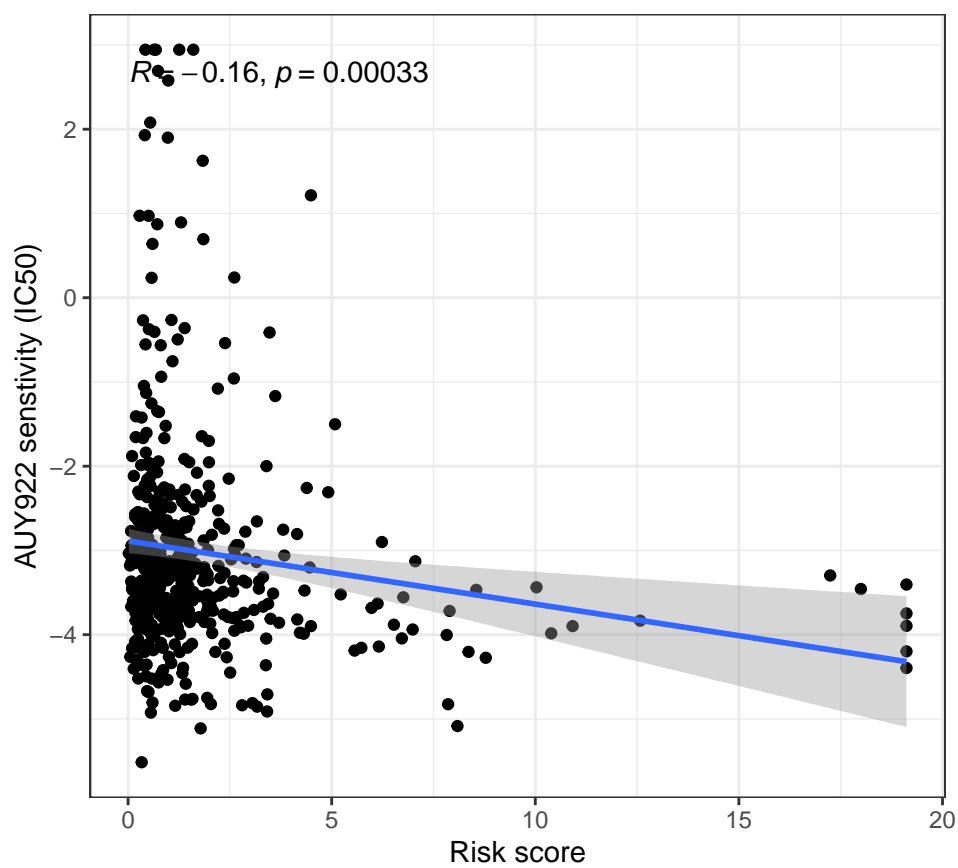

Supplement: Supplementary file 2 — Data S1. [file JCMM-28-e70059-s002.zip › Supplemental Material II/Cor.AUY922.pdf]

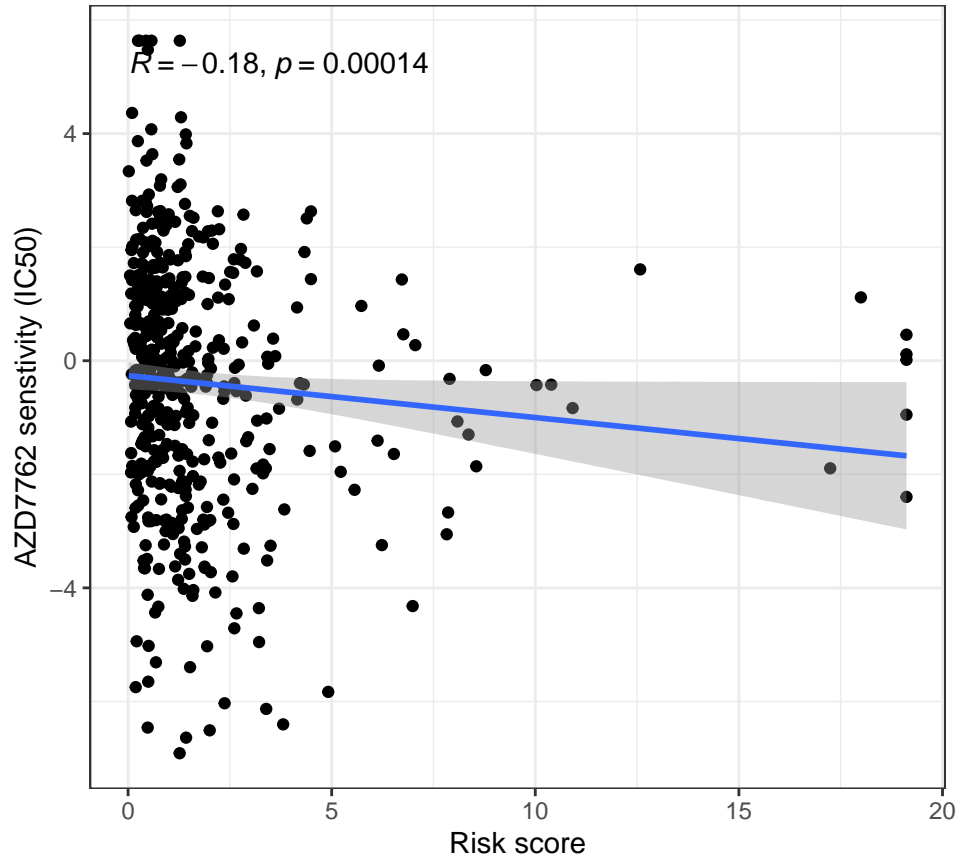

Supplement: Supplementary file 2 — Data S1. [file JCMM-28-e70059-s002.zip › Supplemental Material II/Cor.AZD7762.pdf]

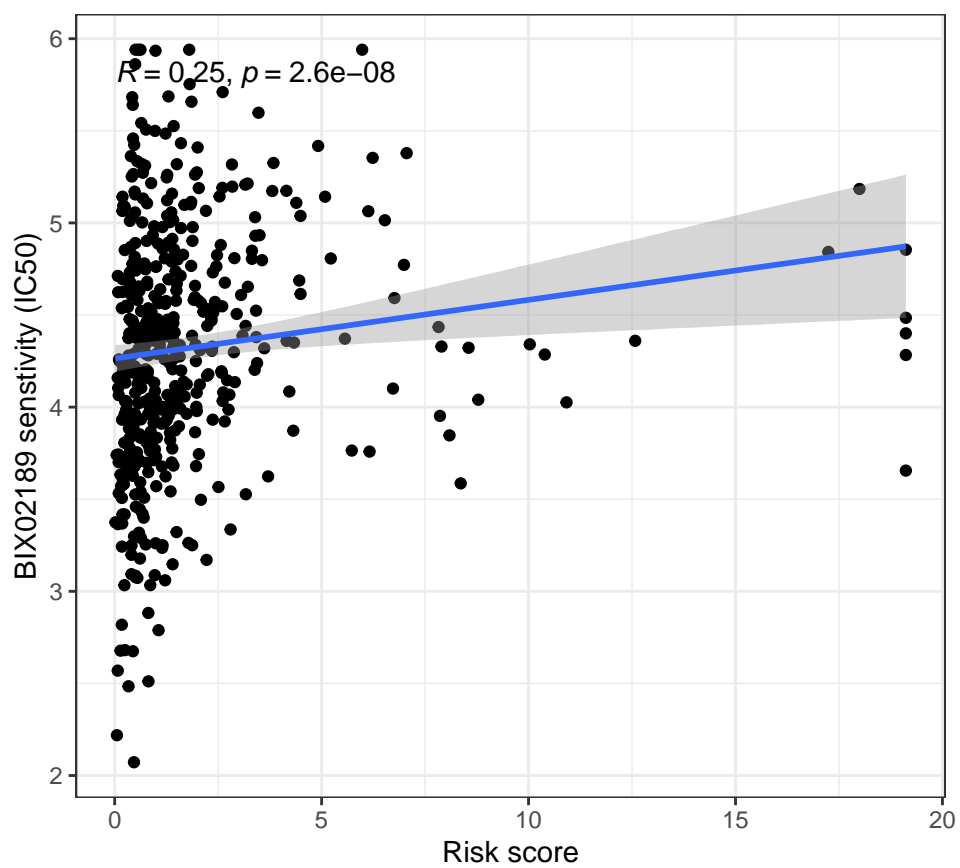

Supplement: Supplementary file 2 — Data S1. [file JCMM-28-e70059-s002.zip › Supplemental Material II/Cor.BIX02189.pdf]

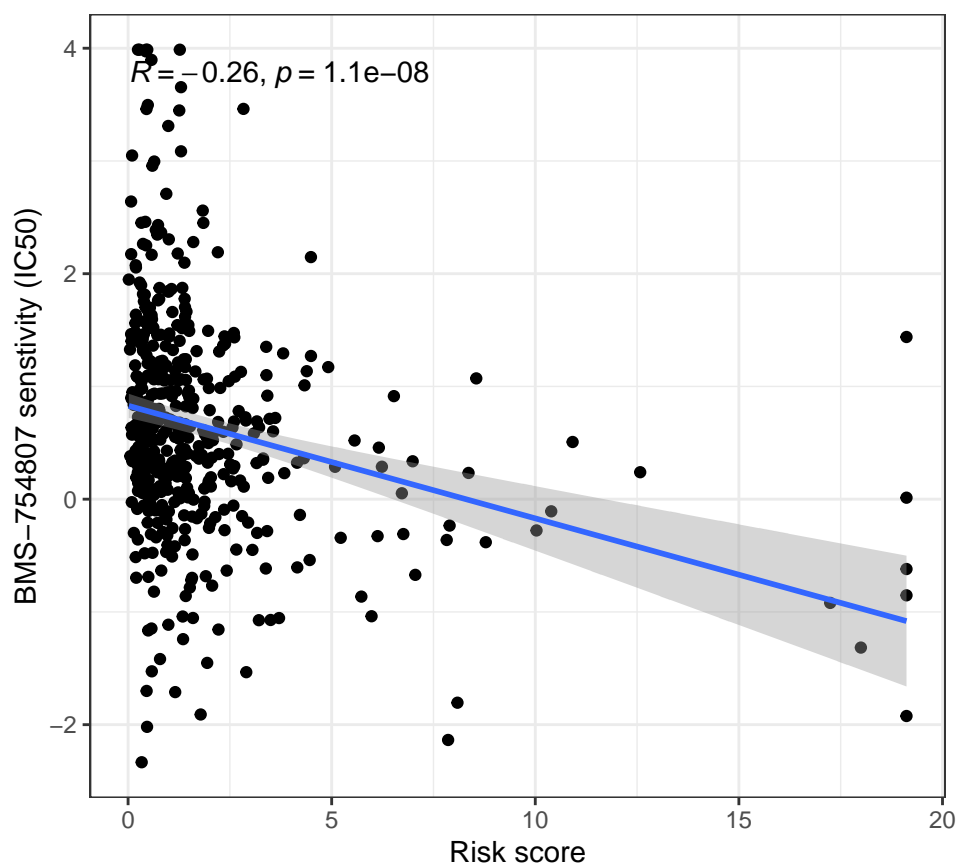

Supplement: Supplementary file 2 — Data S1. [file JCMM-28-e70059-s002.zip › Supplemental Material II/Cor.BMS-754807.pdf]

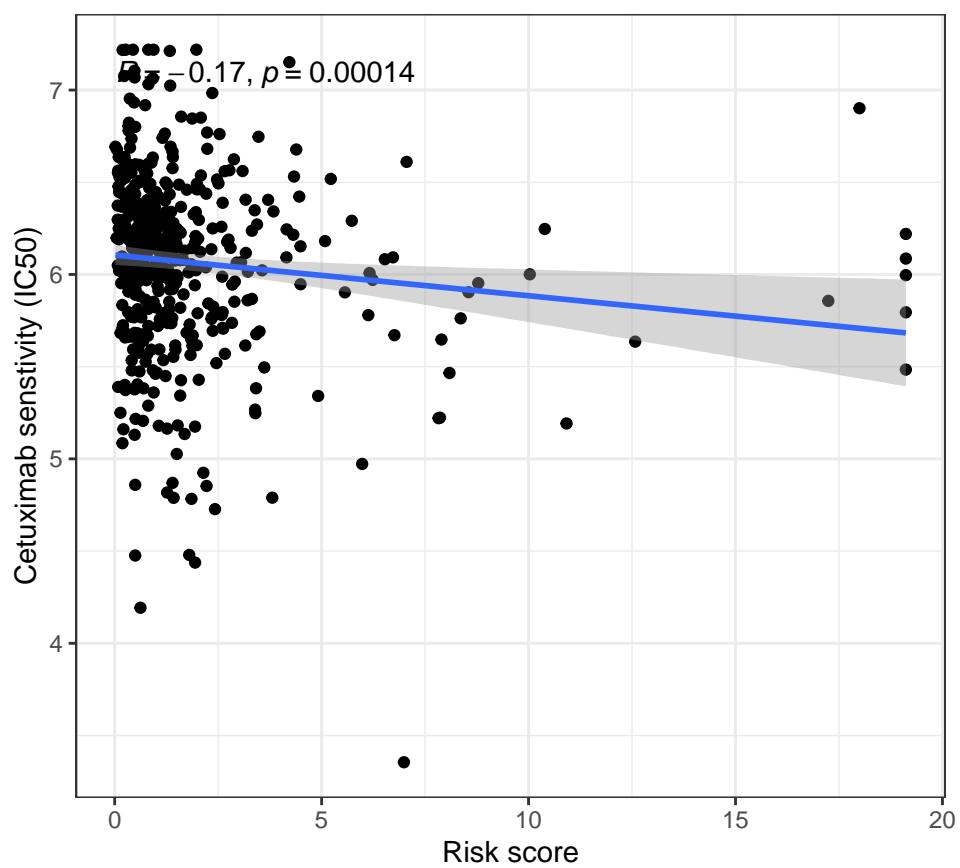

Supplement: Supplementary file 2 — Data S1. [file JCMM-28-e70059-s002.zip › Supplemental Material II/Cor.Cetuximab.pdf]

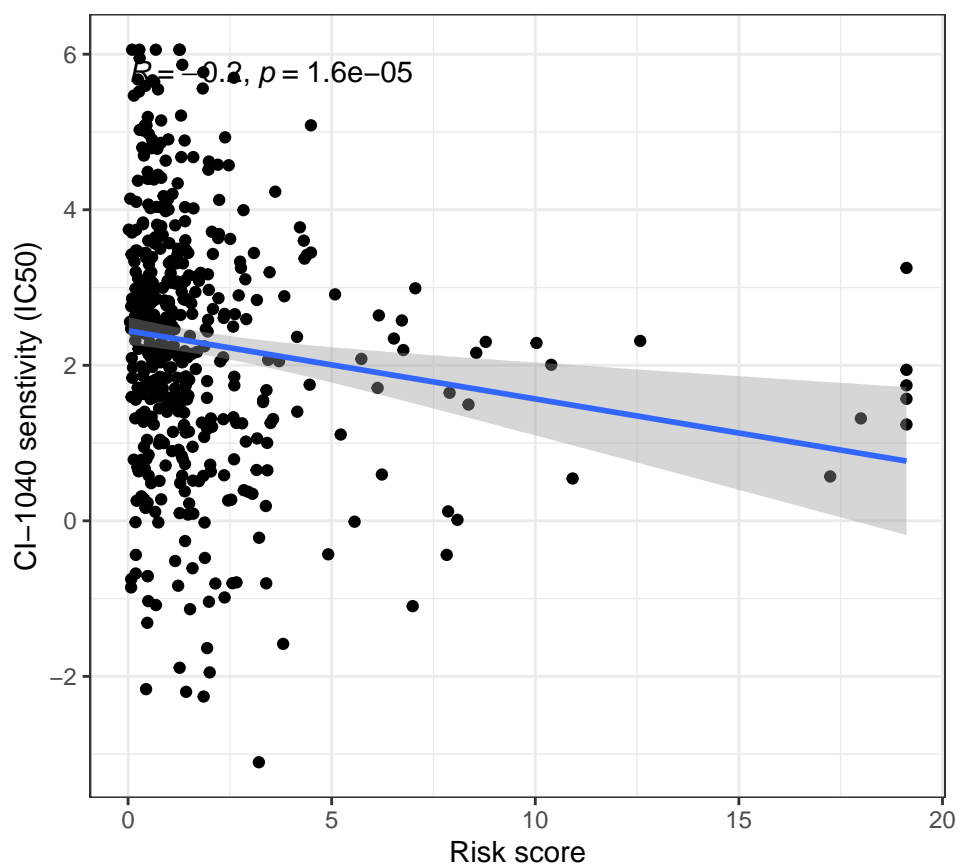

Supplement: Supplementary file 2 — Data S1. [file JCMM-28-e70059-s002.zip › Supplemental Material II/Cor.CI-1040.pdf]

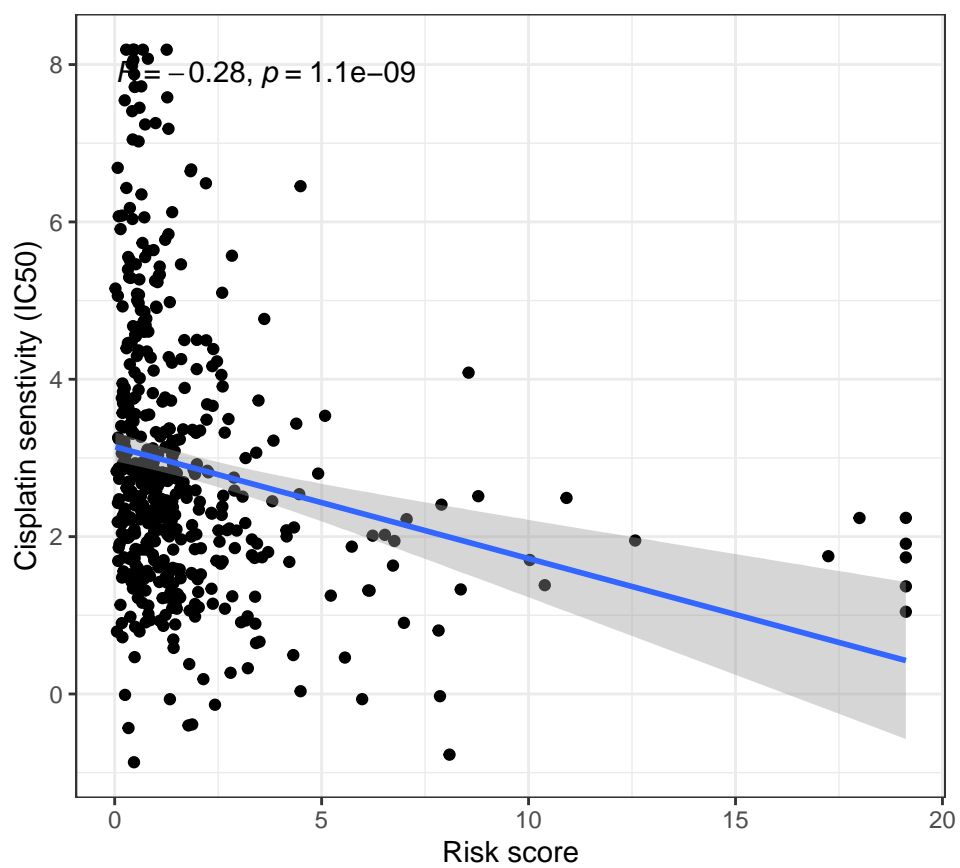

Supplement: Supplementary file 2 — Data S1. [file JCMM-28-e70059-s002.zip › Supplemental Material II/Cor.Cisplatin.pdf]

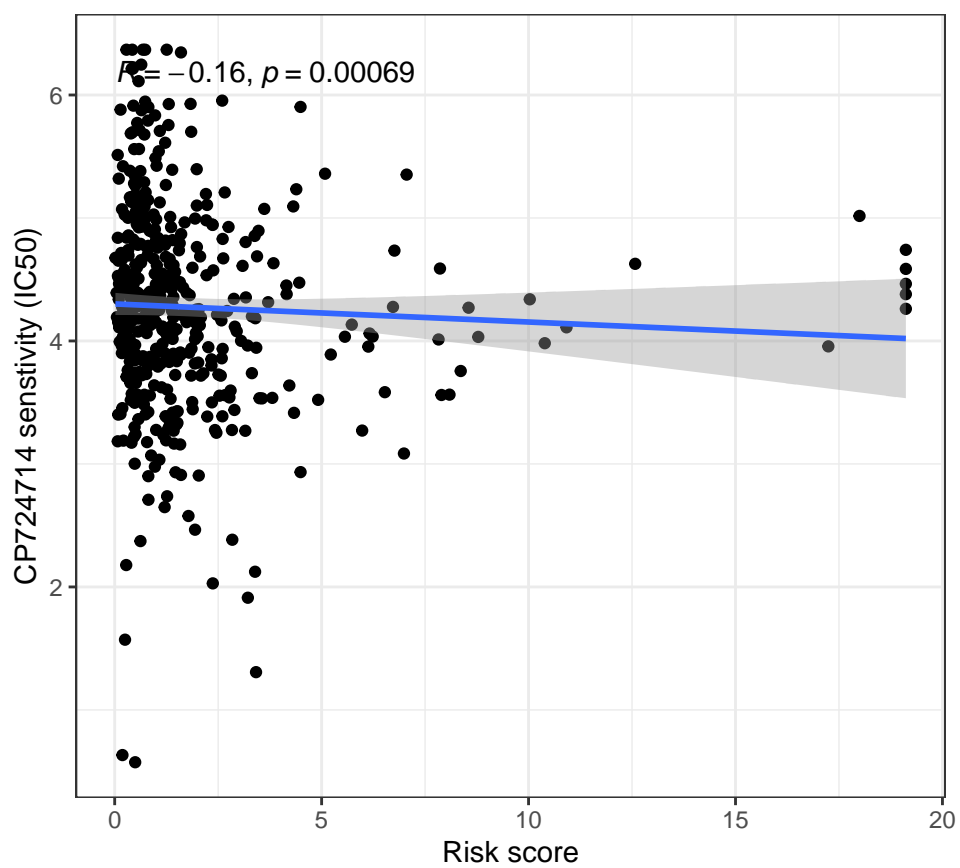

Supplement: Supplementary file 2 — Data S1. [file JCMM-28-e70059-s002.zip › Supplemental Material II/Cor.CP724714.pdf]

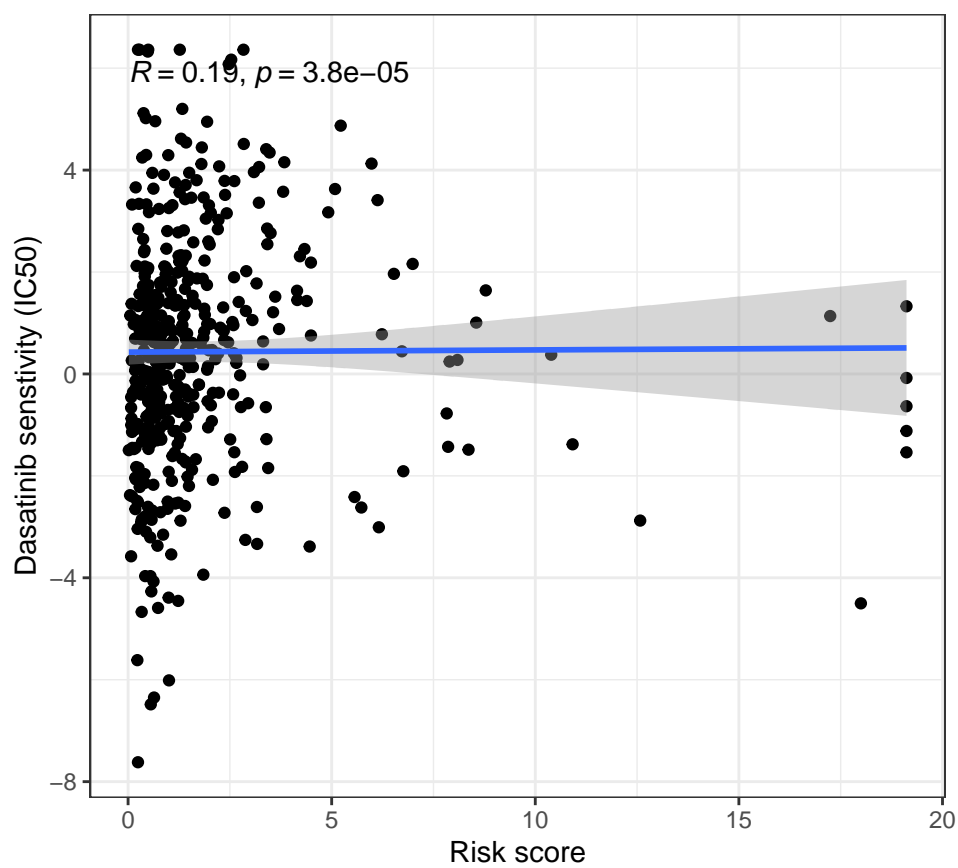

Supplement: Supplementary file 2 — Data S1. [file JCMM-28-e70059-s002.zip › Supplemental Material II/Cor.Dasatinib.pdf]

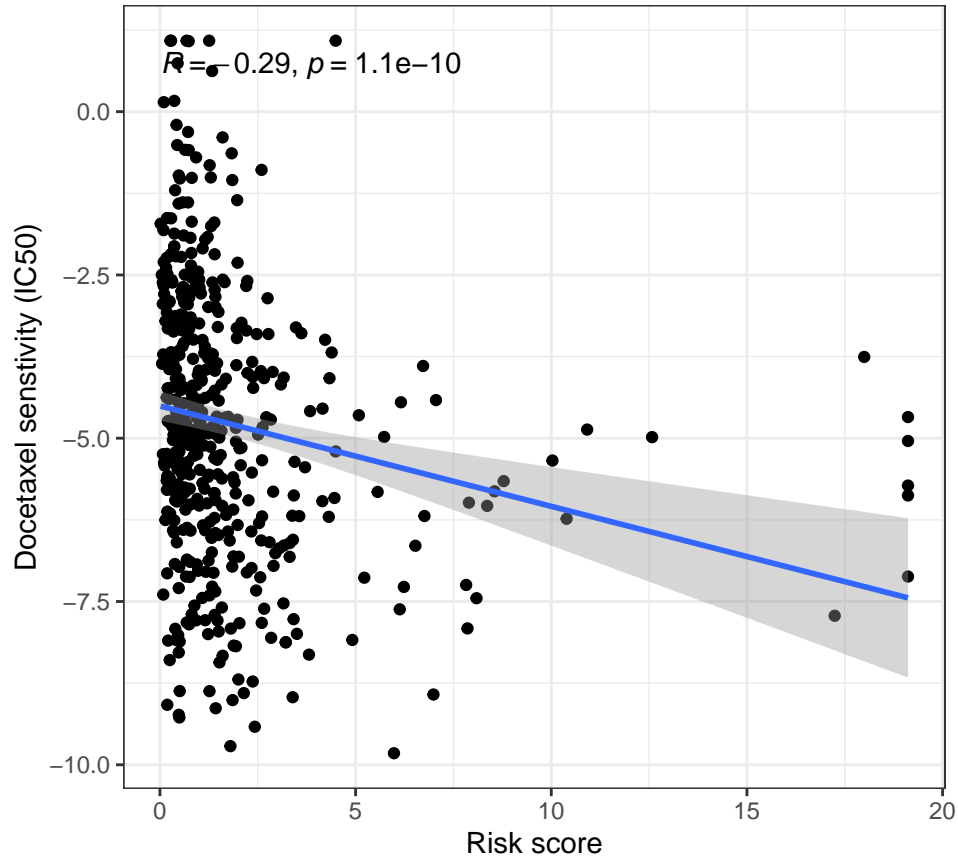

Supplement: Supplementary file 2 — Data S1. [file JCMM-28-e70059-s002.zip › Supplemental Material II/Cor.Docetaxel.pdf]

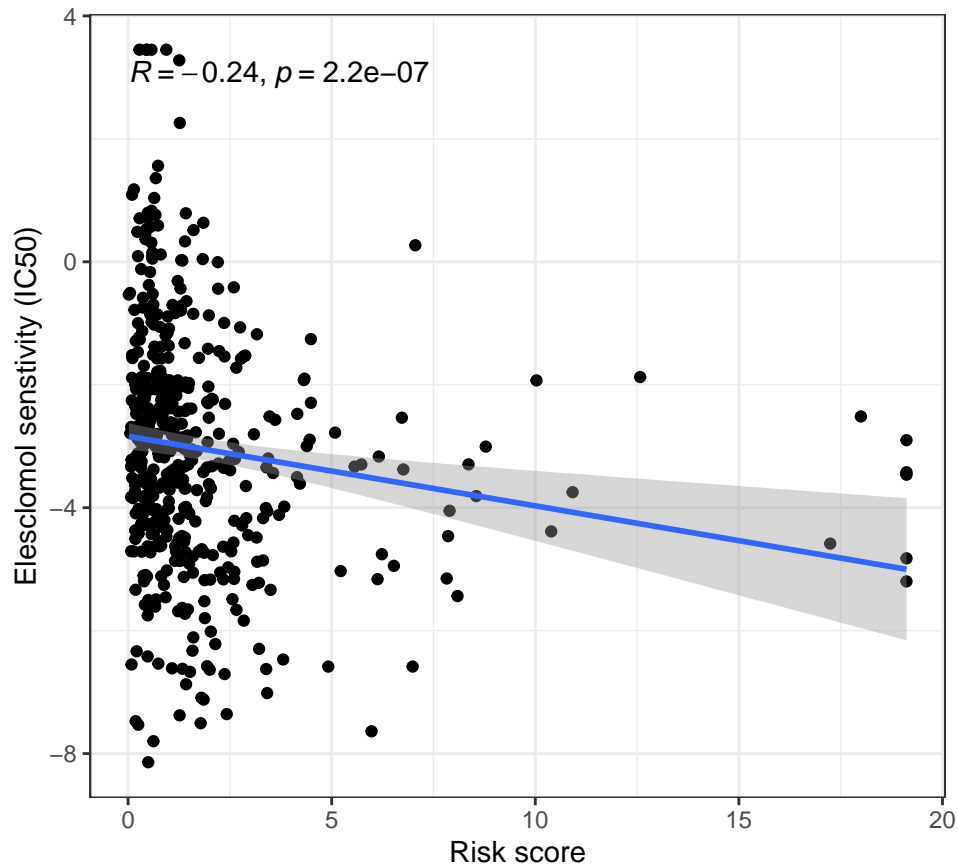

Supplement: Supplementary file 2 — Data S1. [file JCMM-28-e70059-s002.zip › Supplemental Material II/Cor.Elesclomol.pdf]

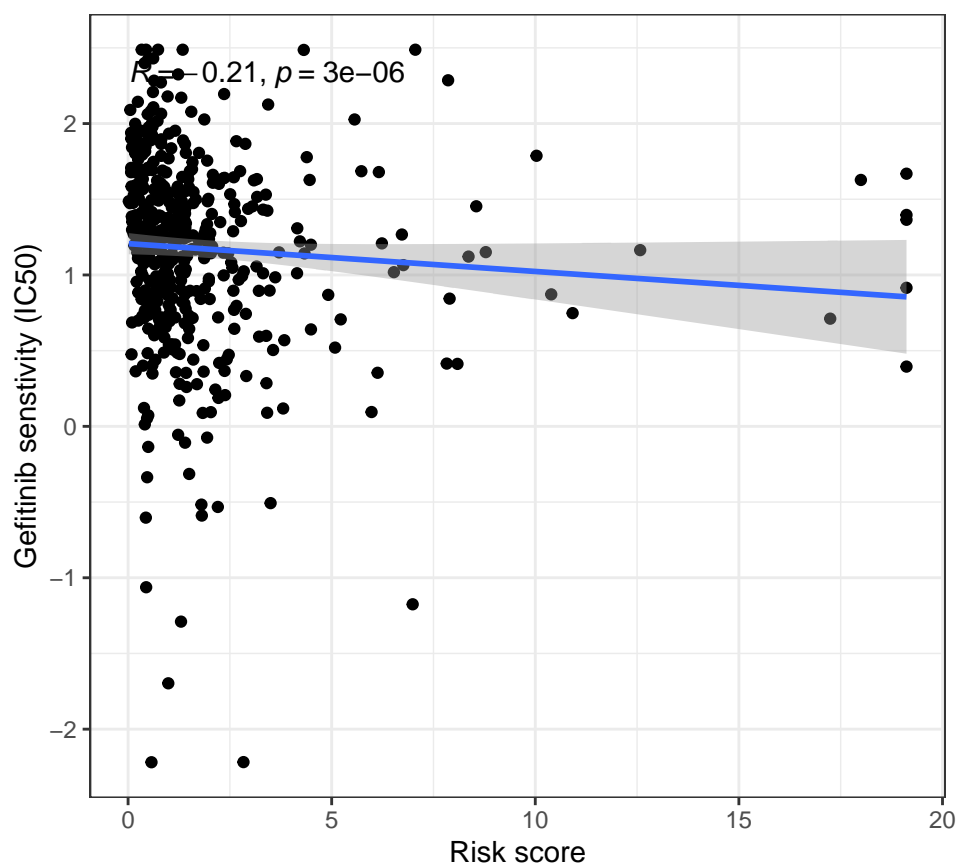

Supplement: Supplementary file 2 — Data S1. [file JCMM-28-e70059-s002.zip › Supplemental Material II/Cor.Gefitinib.pdf]

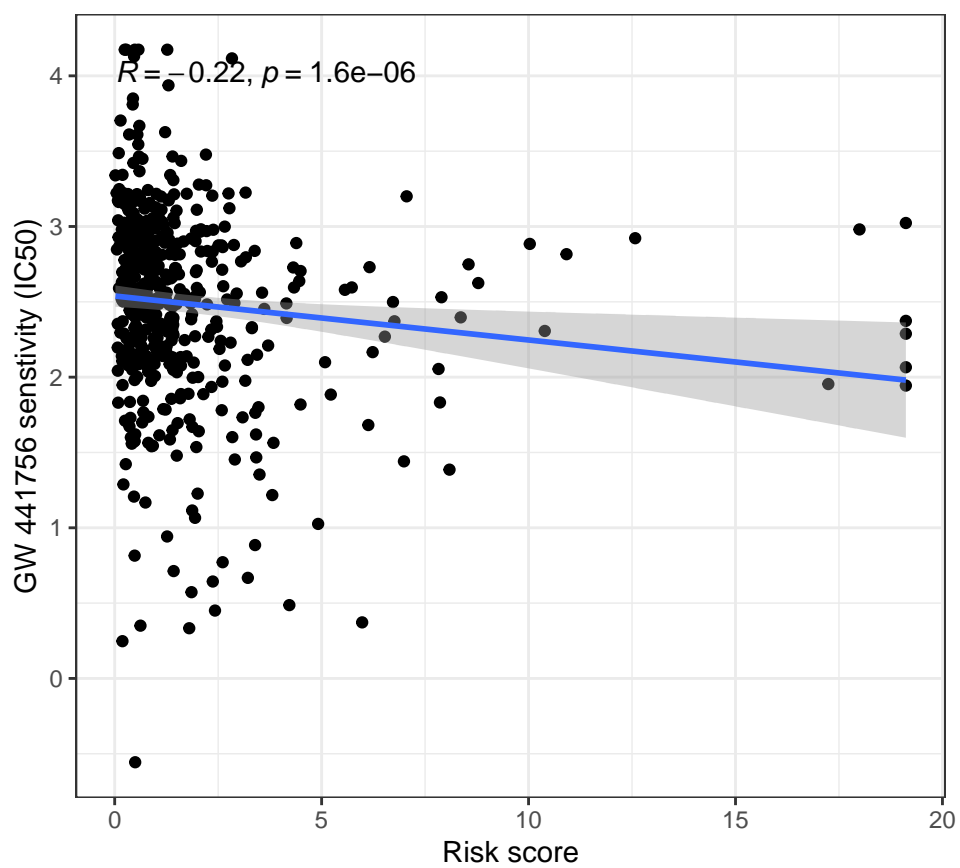

Supplement: Supplementary file 2 — Data S1. [file JCMM-28-e70059-s002.zip › Supplemental Material II/Cor.GW 441756.pdf]

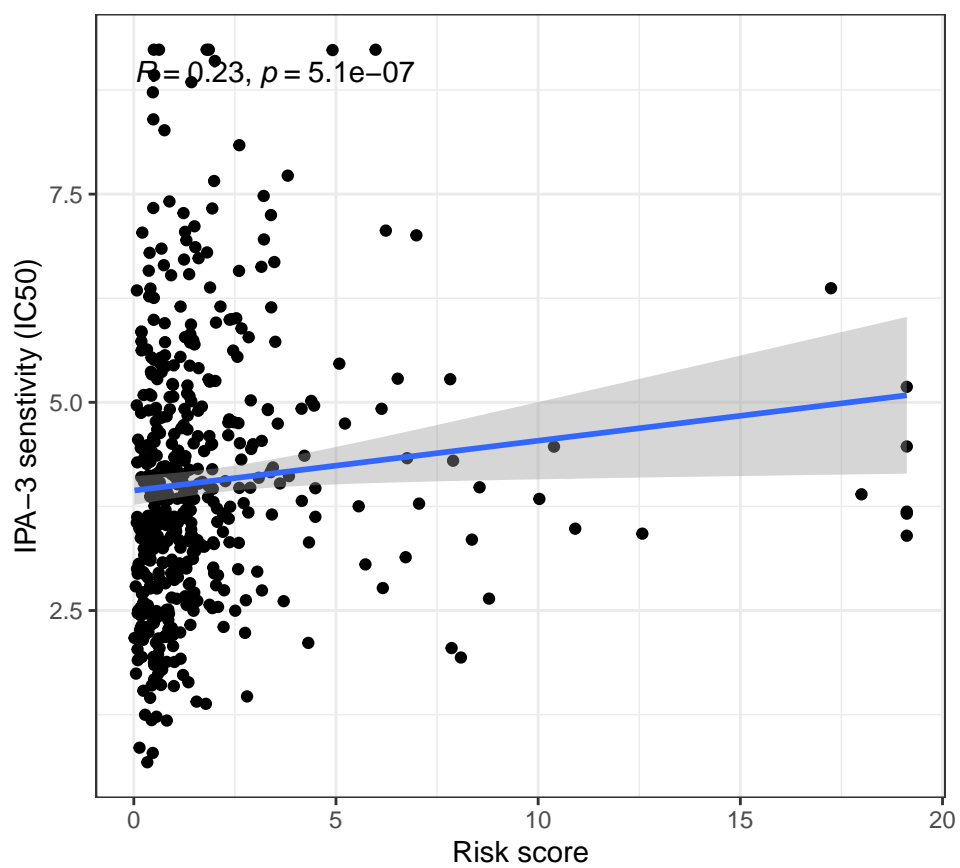

Supplement: Supplementary file 2 — Data S1. [file JCMM-28-e70059-s002.zip › Supplemental Material II/Cor.IPA-3.pdf]

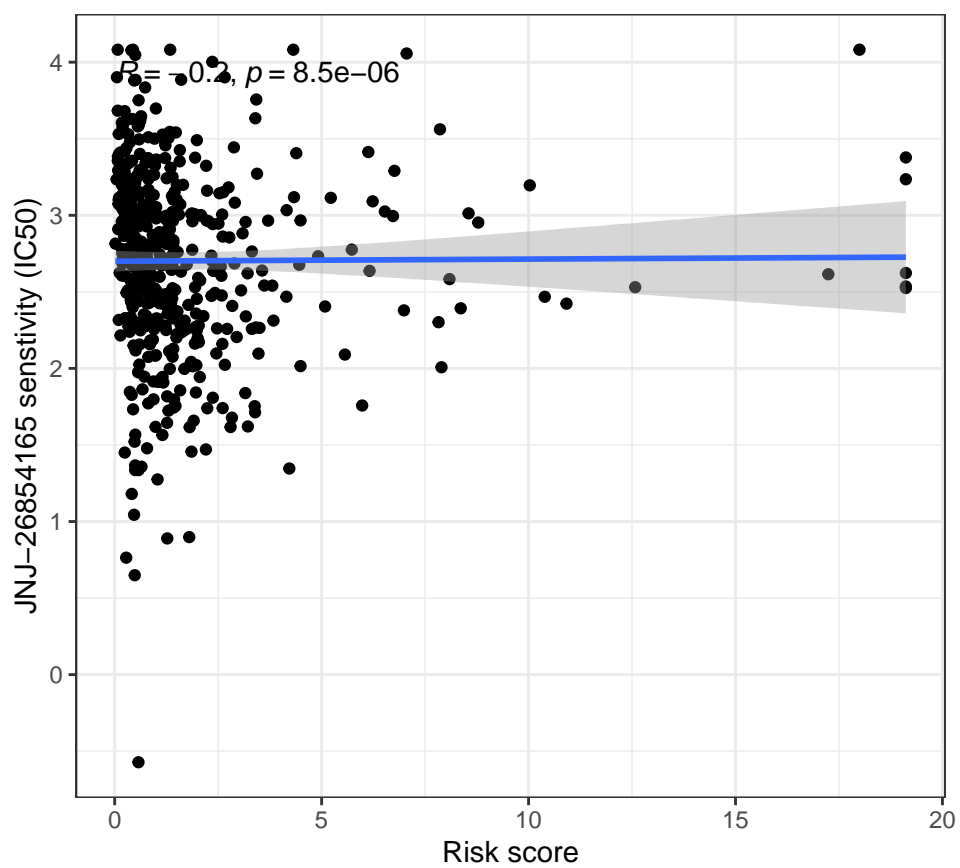

Supplement: Supplementary file 2 — Data S1. [file JCMM-28-e70059-s002.zip › Supplemental Material II/Cor.JNJ-26854165.pdf]

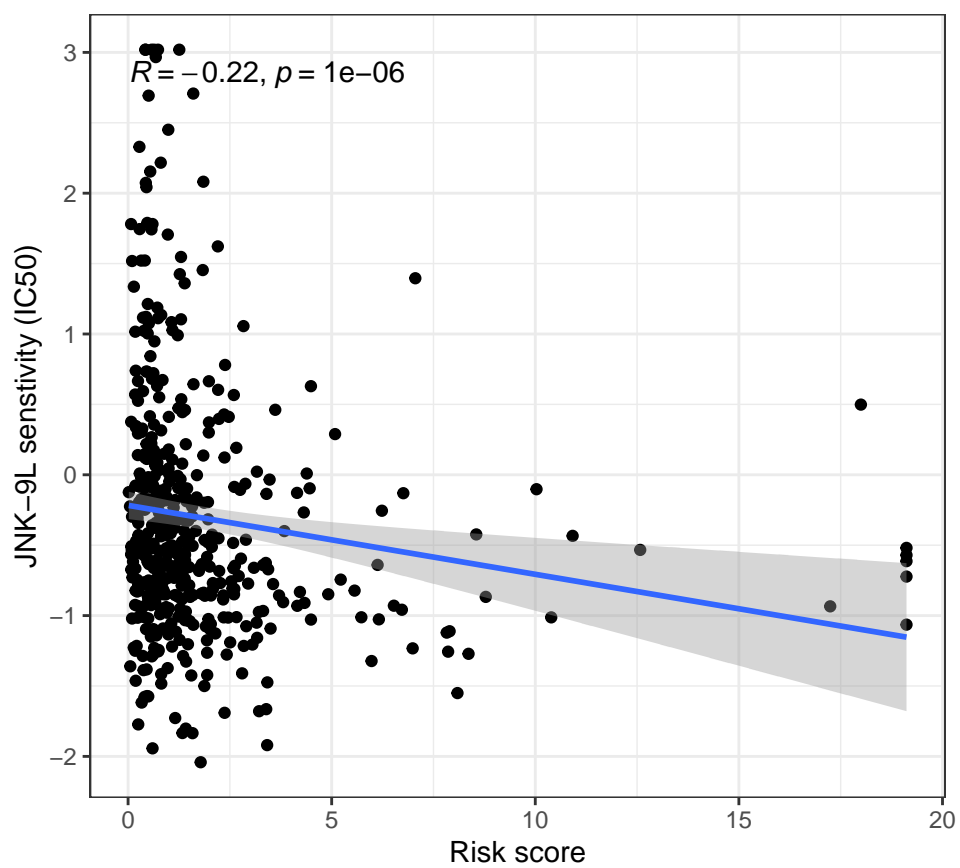

Supplement: Supplementary file 2 — Data S1. [file JCMM-28-e70059-s002.zip › Supplemental Material II/Cor.JNK-9L.pdf]

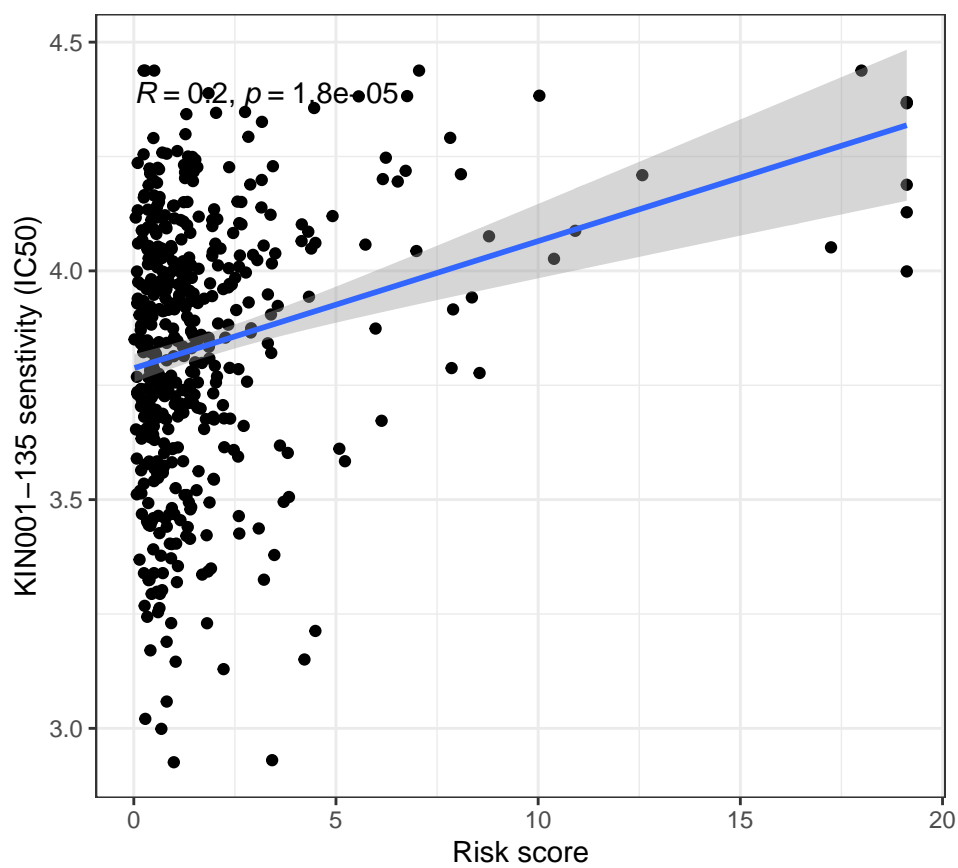

Supplement: Supplementary file 2 — Data S1. [file JCMM-28-e70059-s002.zip › Supplemental Material II/Cor.KIN001-135.pdf]

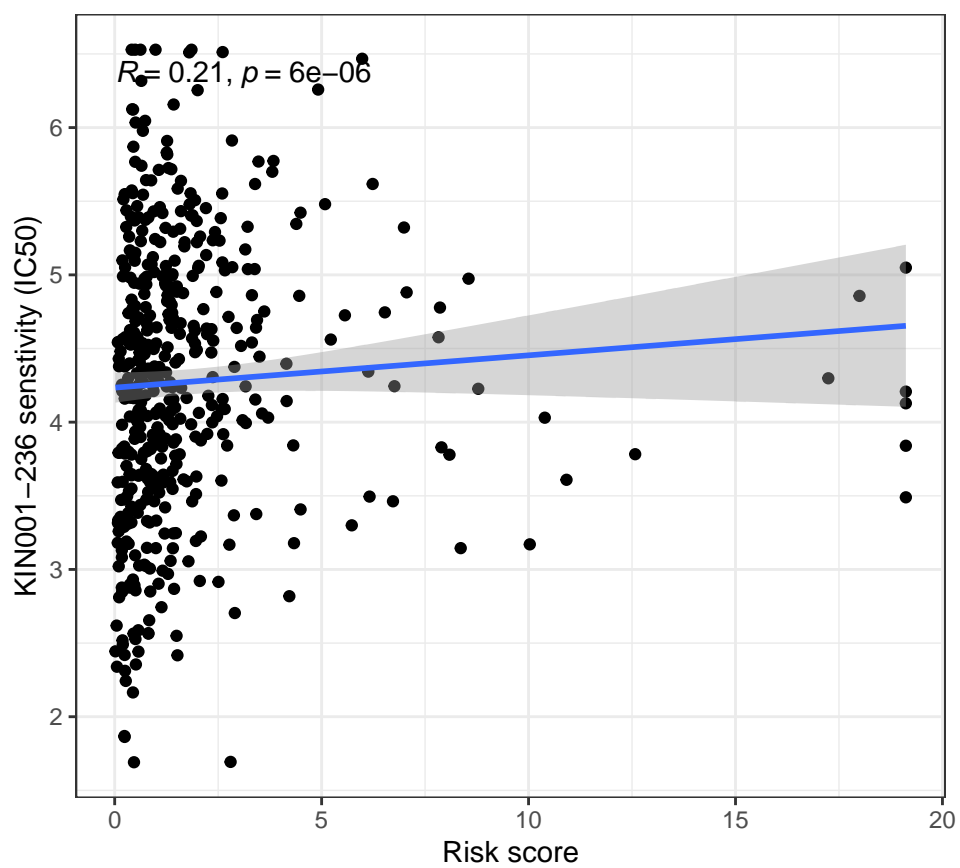

Supplement: Supplementary file 2 — Data S1. [file JCMM-28-e70059-s002.zip › Supplemental Material II/Cor.KIN001-236.pdf]

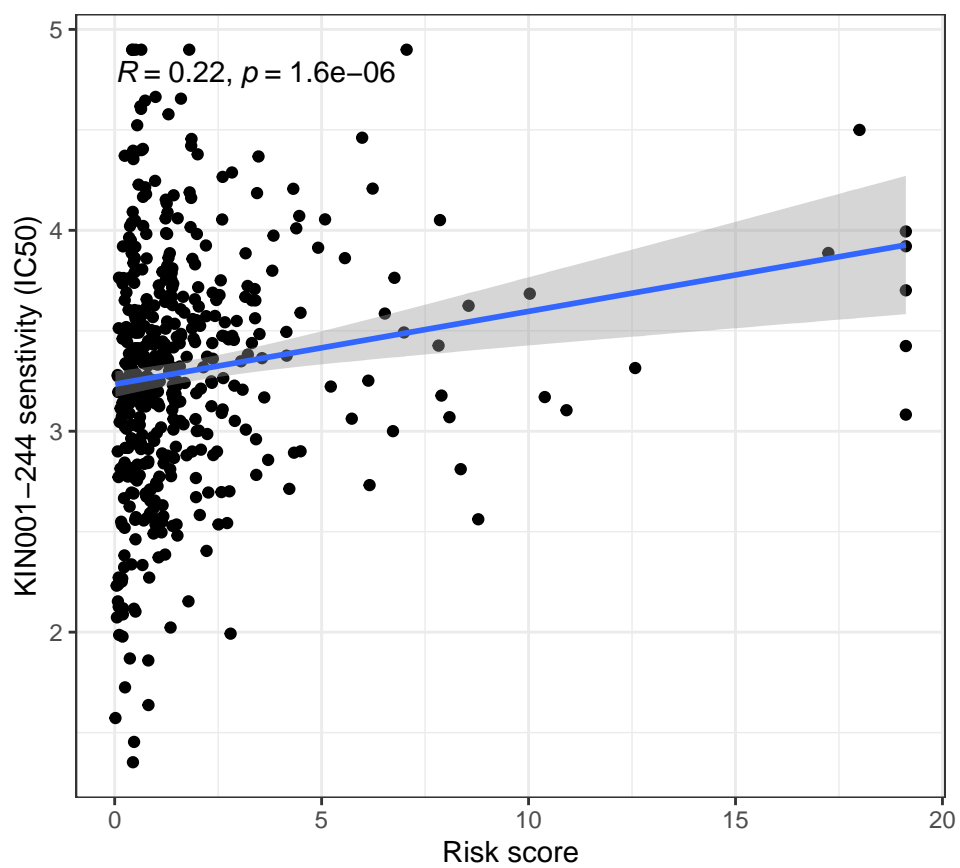

Supplement: Supplementary file 2 — Data S1. [file JCMM-28-e70059-s002.zip › Supplemental Material II/Cor.KIN001-244.pdf]

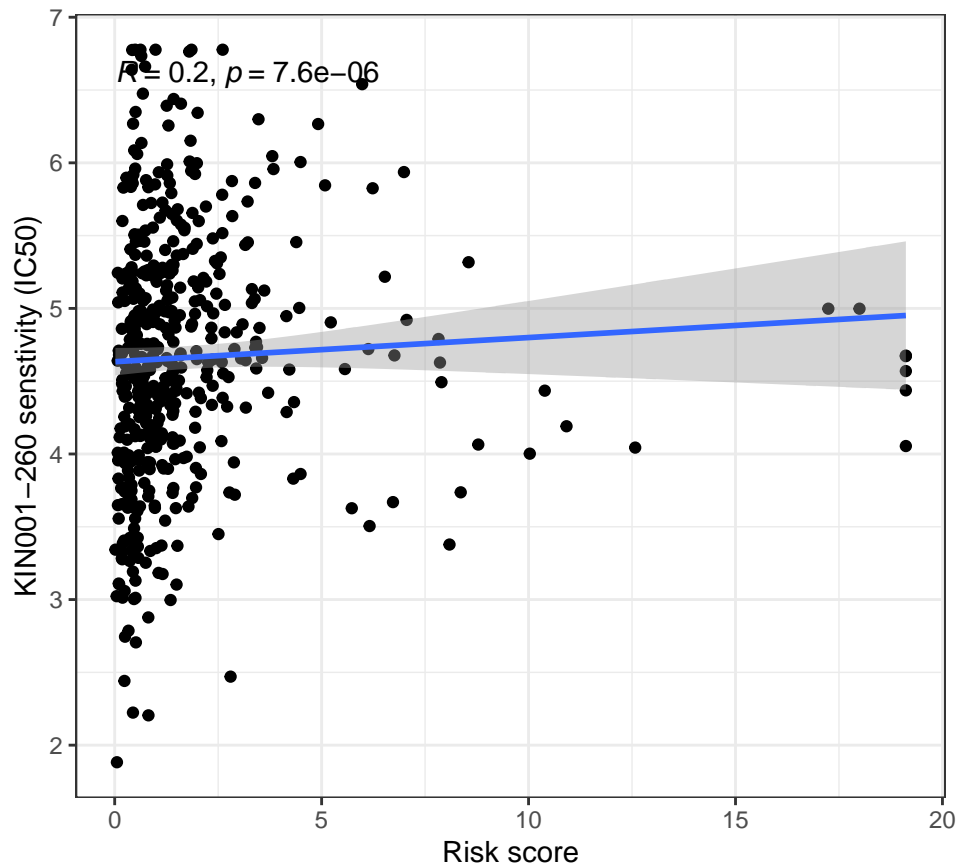

Supplement: Supplementary file 2 — Data S1. [file JCMM-28-e70059-s002.zip › Supplemental Material II/Cor.KIN001-260.pdf]

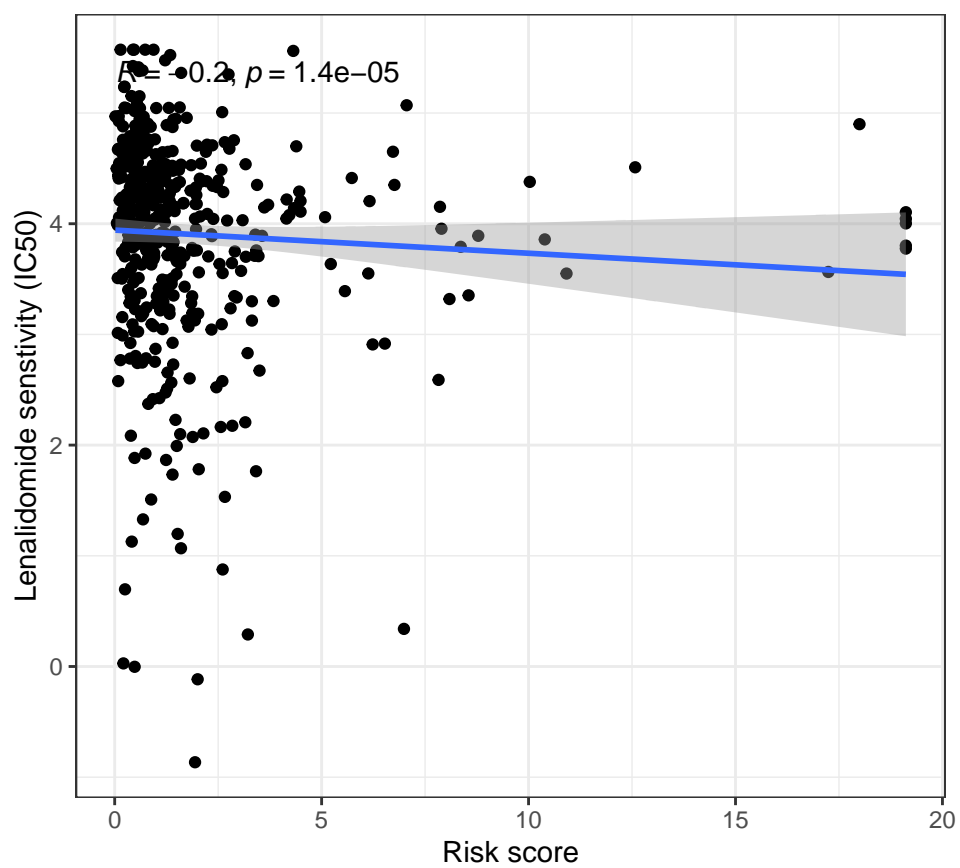

Supplement: Supplementary file 2 — Data S1. [file JCMM-28-e70059-s002.zip › Supplemental Material II/Cor.Lenalidomide.pdf]

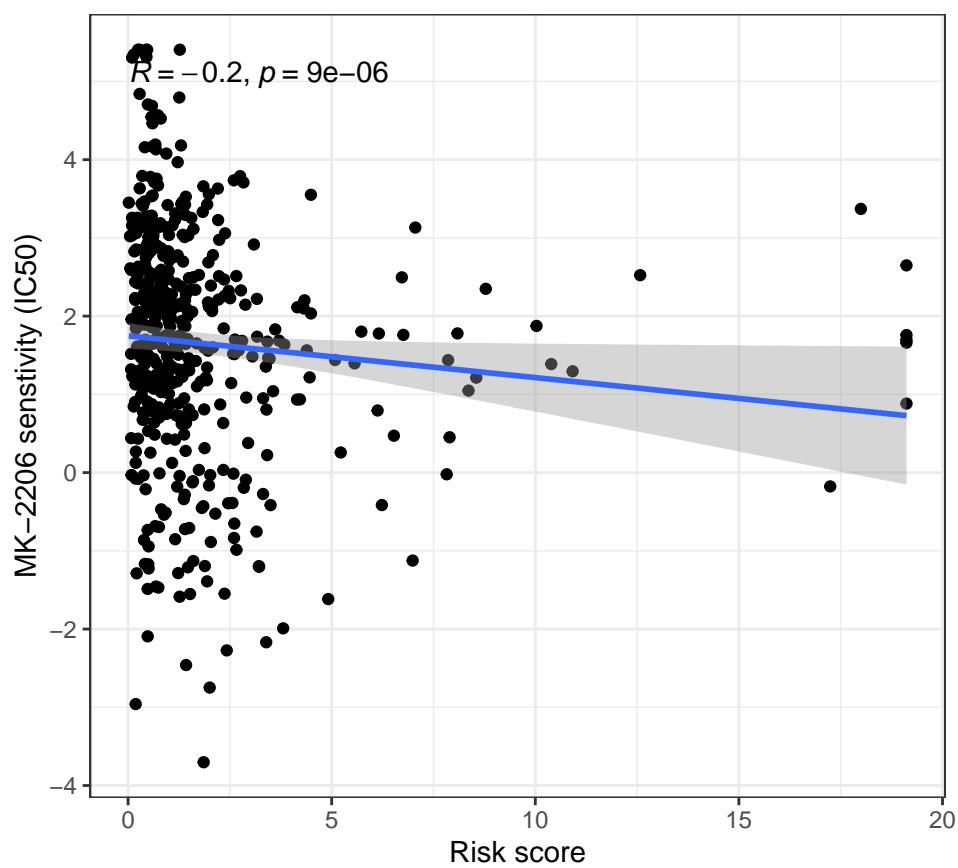

Supplement: Supplementary file 2 — Data S1. [file JCMM-28-e70059-s002.zip › Supplemental Material II/Cor.MK-2206.pdf]

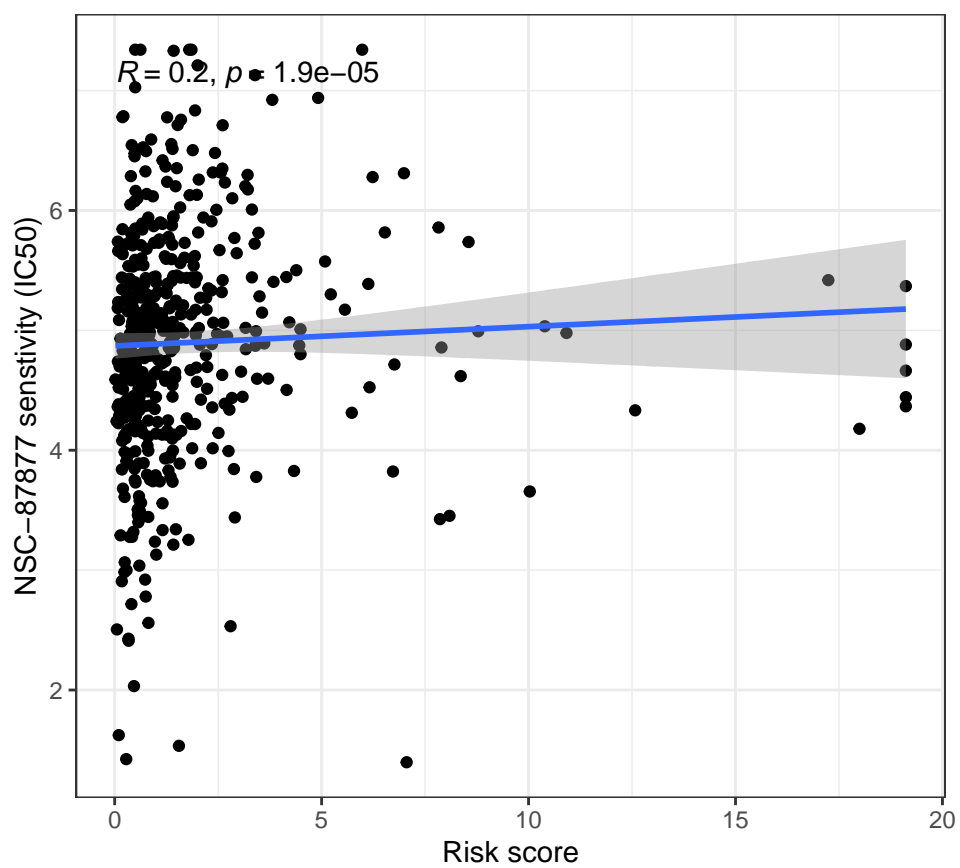

Supplement: Supplementary file 2 — Data S1. [file JCMM-28-e70059-s002.zip › Supplemental Material II/Cor.NSC-87877.pdf]

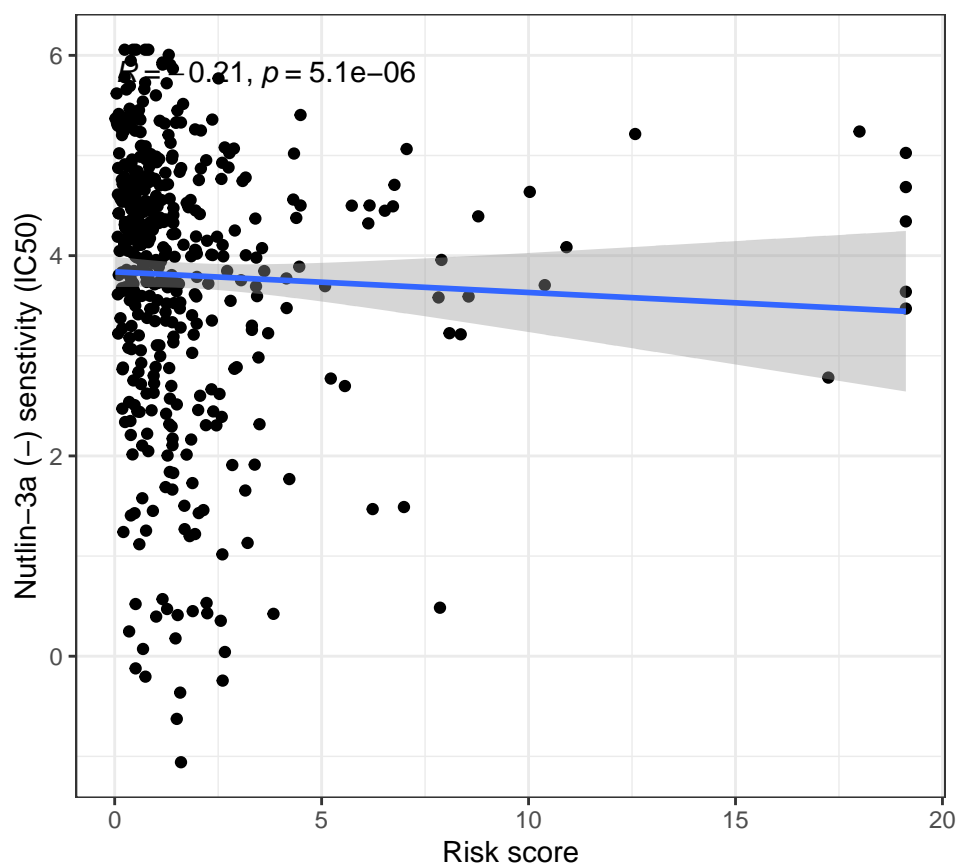

Supplement: Supplementary file 2 — Data S1. [file JCMM-28-e70059-s002.zip › Supplemental Material II/Cor.Nutlin-3a (-).pdf]

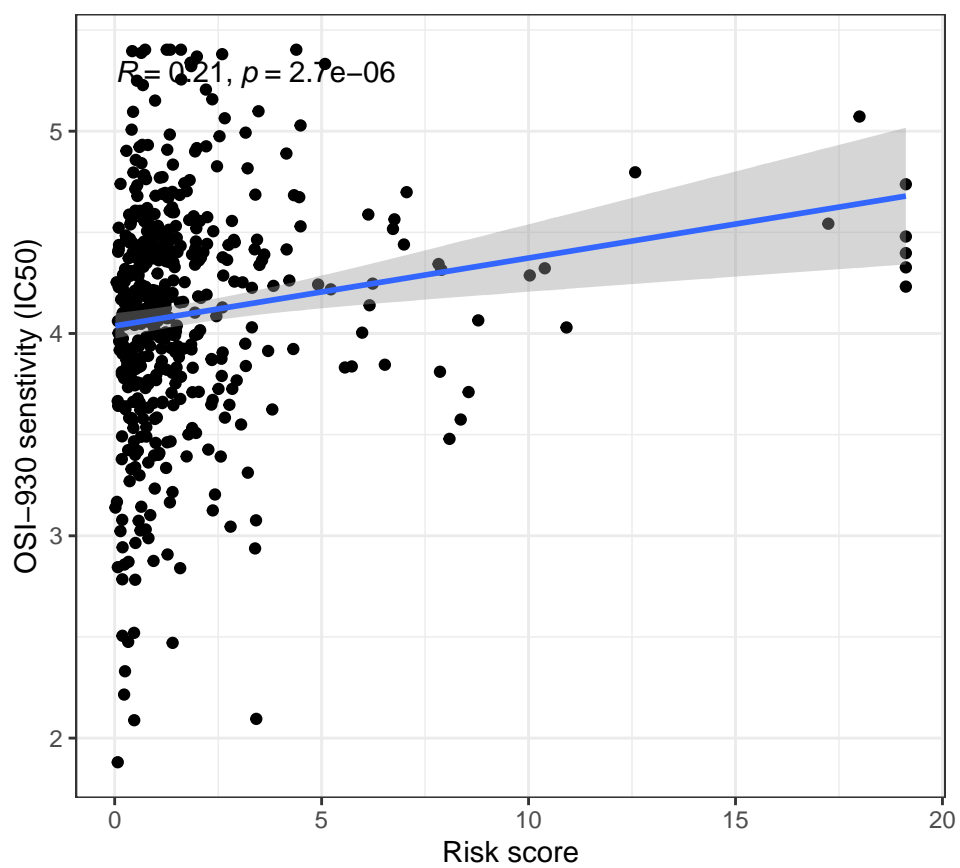

Supplement: Supplementary file 2 — Data S1. [file JCMM-28-e70059-s002.zip › Supplemental Material II/Cor.OSI-930.pdf]

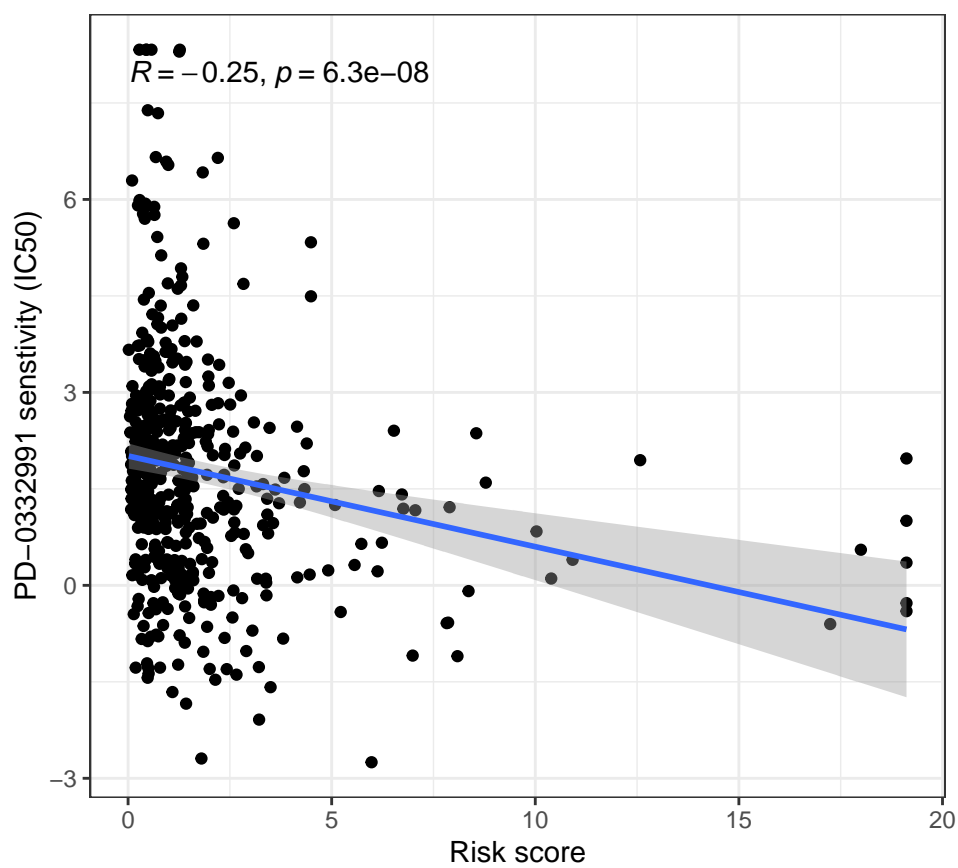

Supplement: Supplementary file 2 — Data S1. [file JCMM-28-e70059-s002.zip › Supplemental Material II/Cor.PD-0332991.pdf]

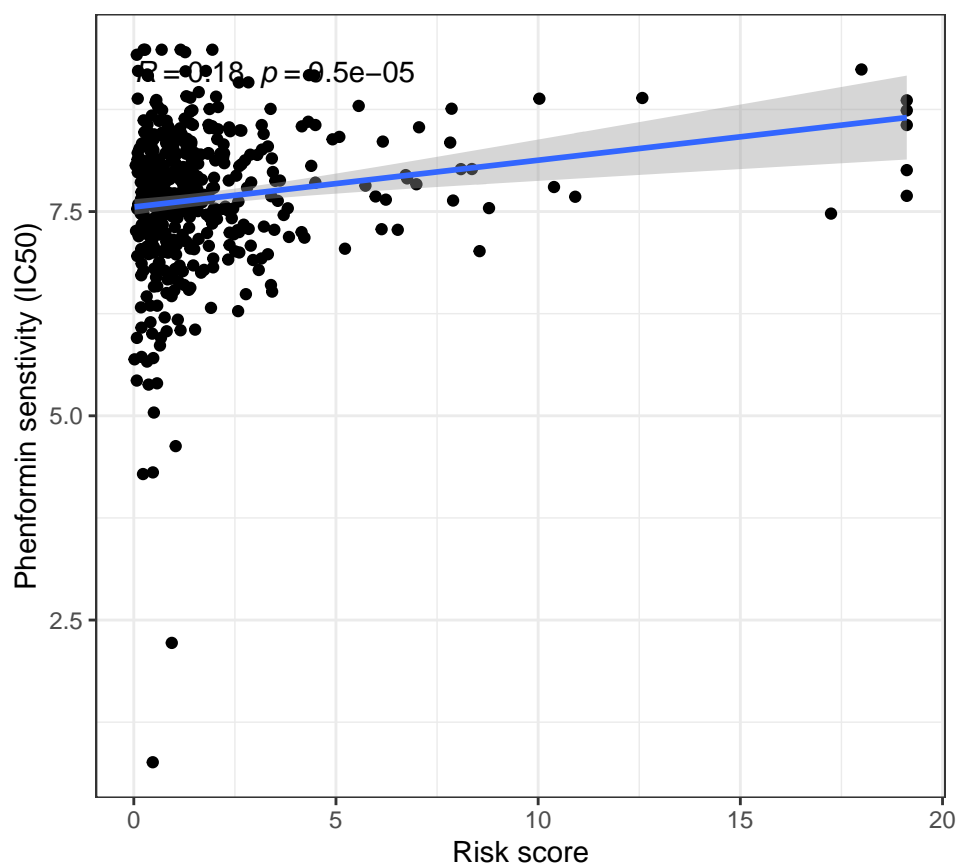

Supplement: Supplementary file 2 — Data S1. [file JCMM-28-e70059-s002.zip › Supplemental Material II/Cor.Phenformin.pdf]

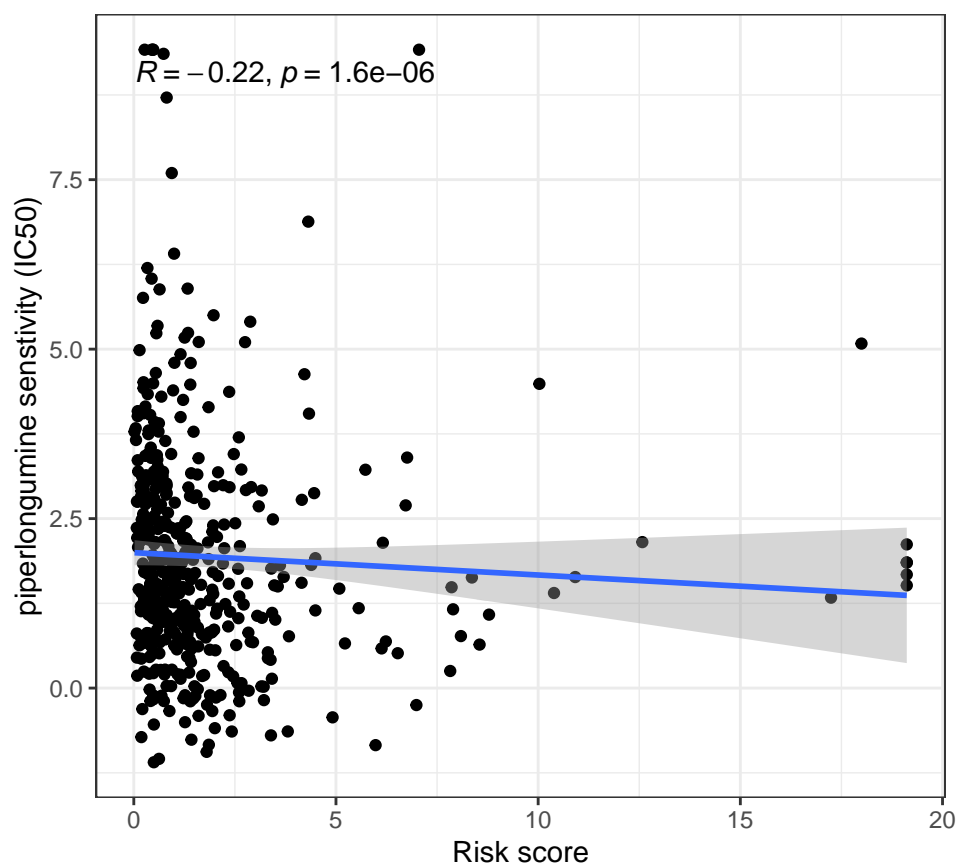

Supplement: Supplementary file 2 — Data S1. [file JCMM-28-e70059-s002.zip › Supplemental Material II/Cor.piperlongumine.pdf]

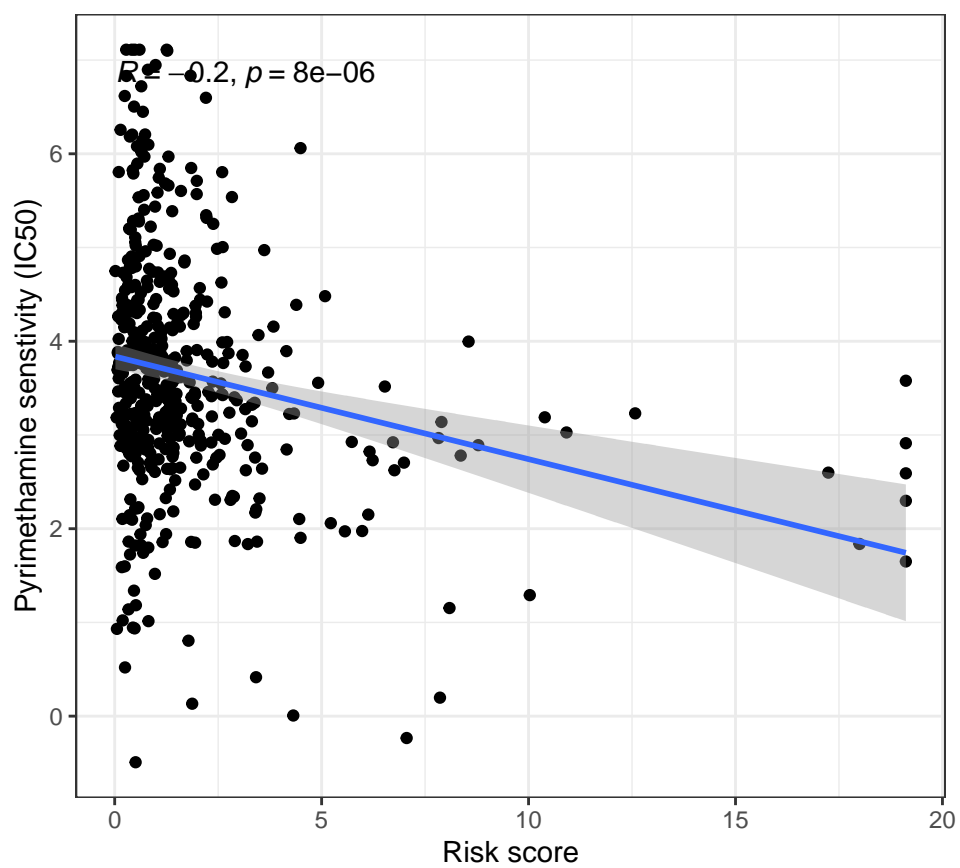

Supplement: Supplementary file 2 — Data S1. [file JCMM-28-e70059-s002.zip › Supplemental Material II/Cor.Pyrimethamine.pdf]

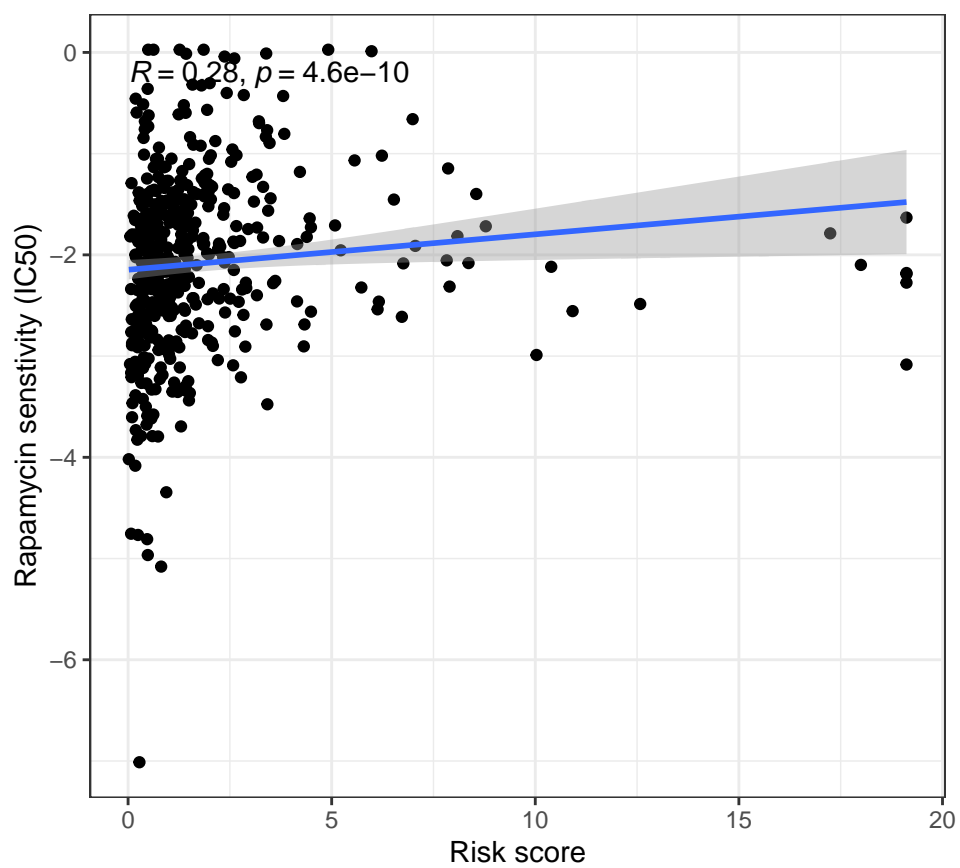

Supplement: Supplementary file 2 — Data S1. [file JCMM-28-e70059-s002.zip › Supplemental Material II/Cor.Rapamycin.pdf]

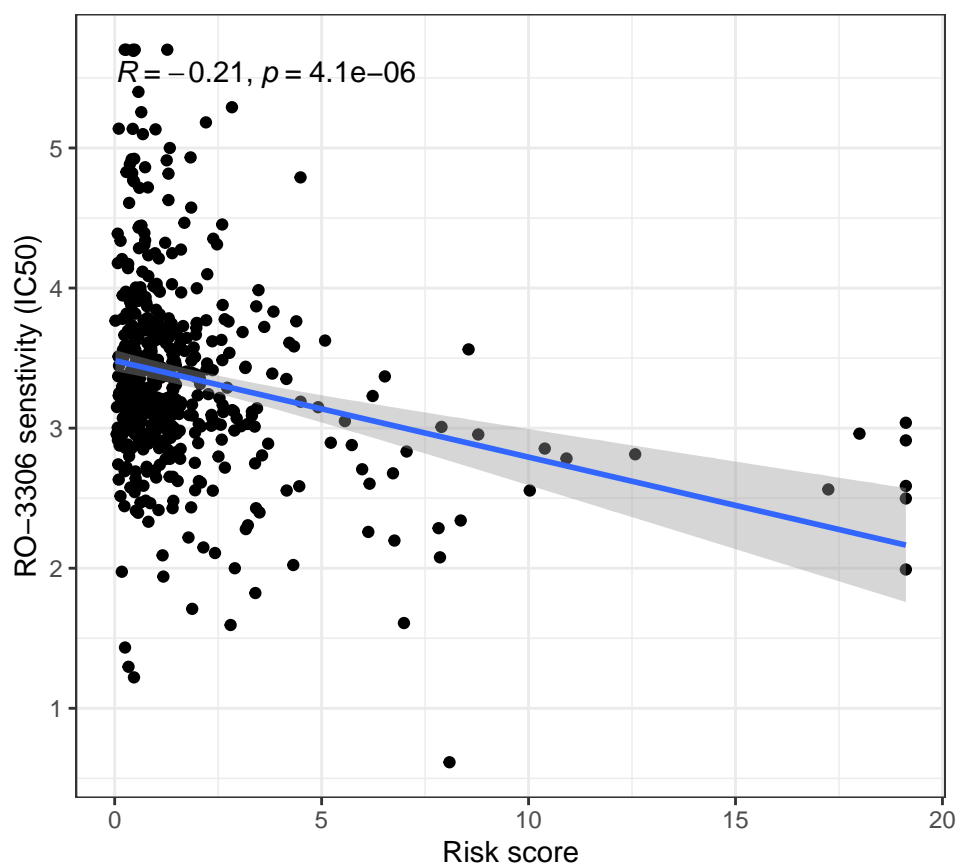

Supplement: Supplementary file 2 — Data S1. [file JCMM-28-e70059-s002.zip › Supplemental Material II/Cor.RO-3306.pdf]

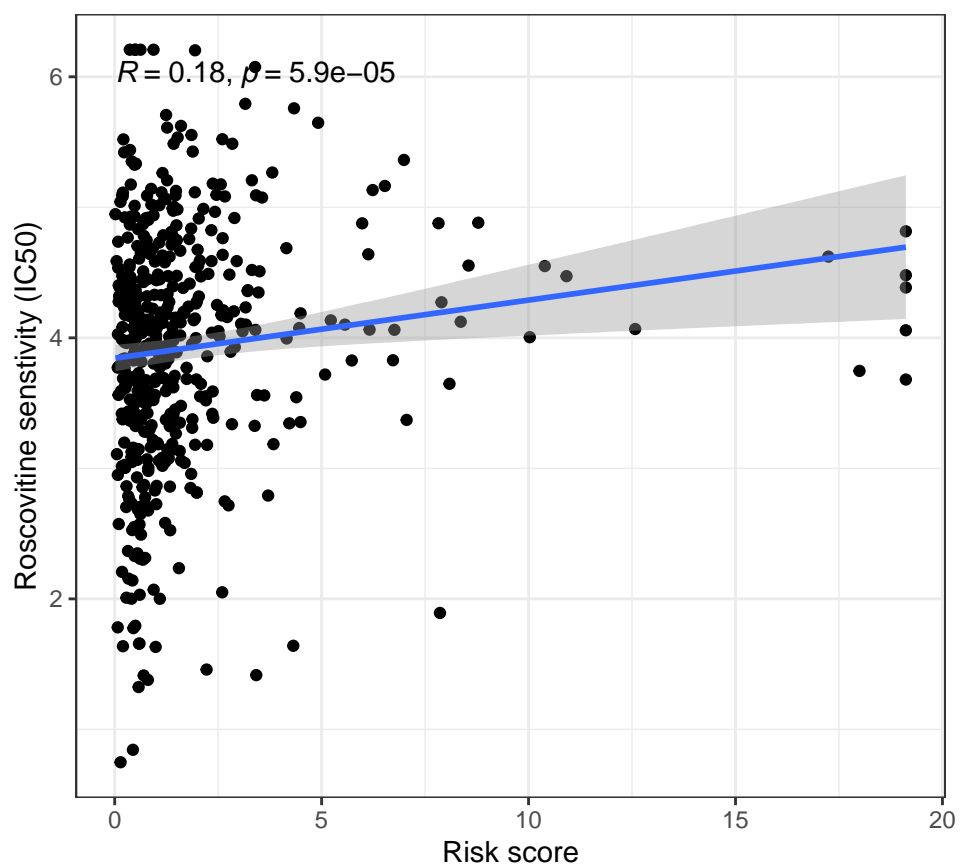

Supplement: Supplementary file 2 — Data S1. [file JCMM-28-e70059-s002.zip › Supplemental Material II/Cor.Roscovitine.pdf]

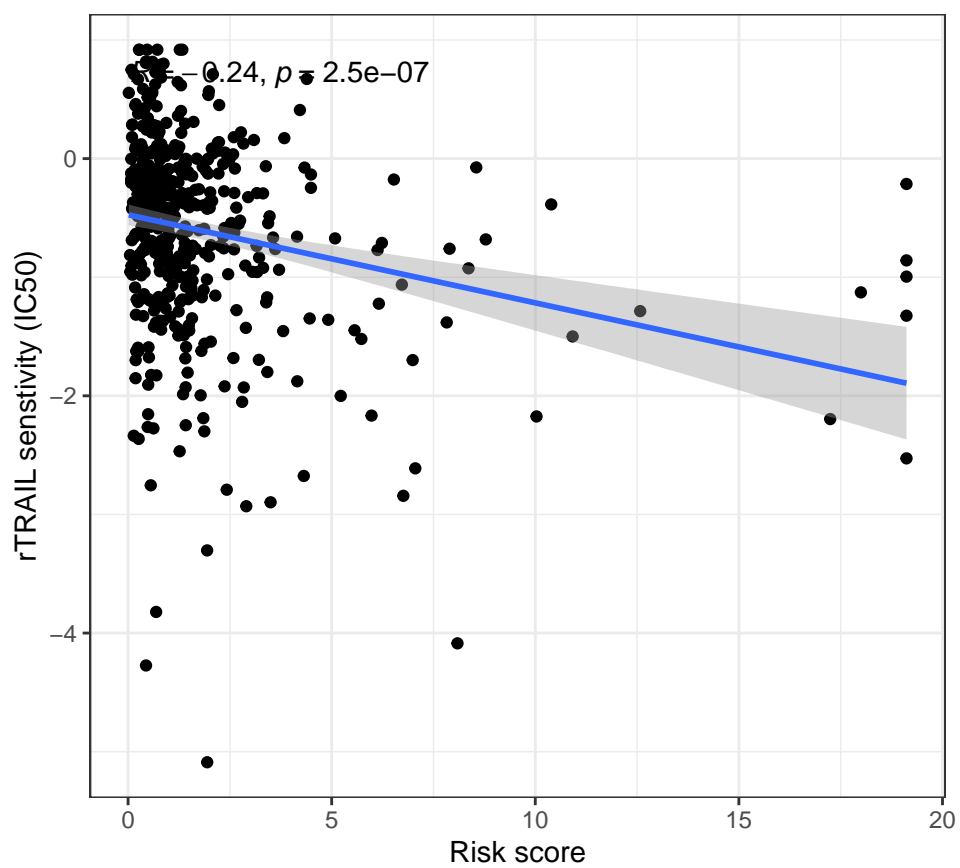

Supplement: Supplementary file 2 — Data S1. [file JCMM-28-e70059-s002.zip › Supplemental Material II/Cor.rTRAIL.pdf]

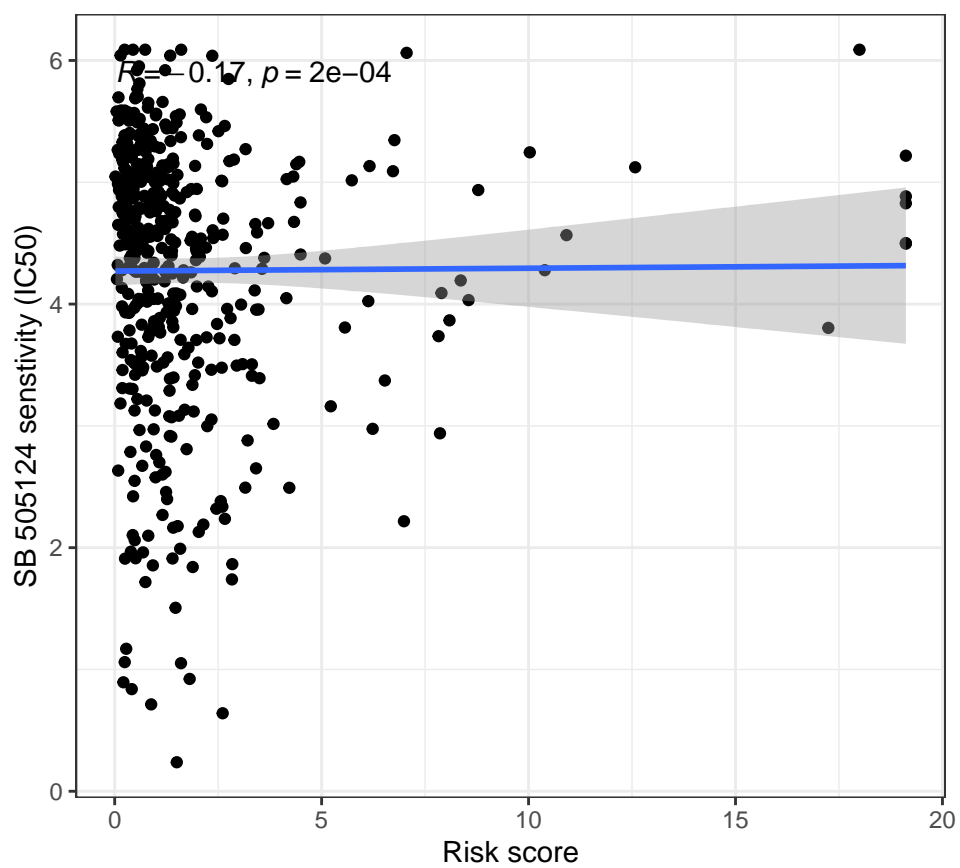

Supplement: Supplementary file 2 — Data S1. [file JCMM-28-e70059-s002.zip › Supplemental Material II/Cor.SB 505124.pdf]

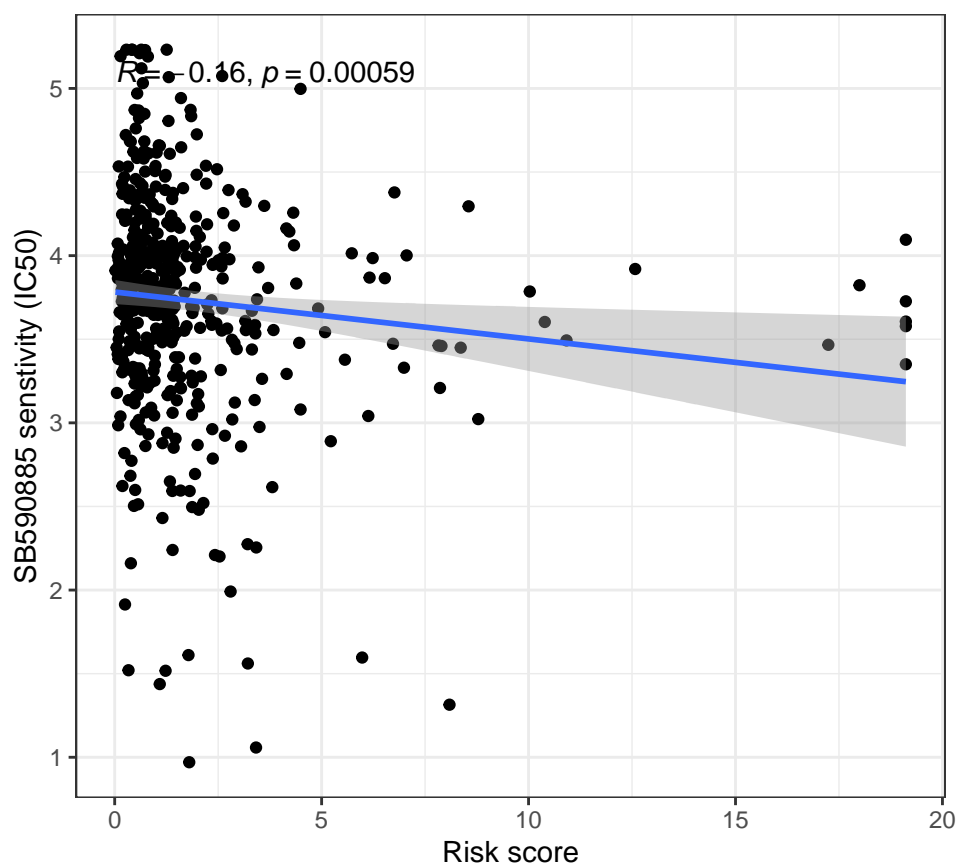

Supplement: Supplementary file 2 — Data S1. [file JCMM-28-e70059-s002.zip › Supplemental Material II/Cor.SB590885.pdf]

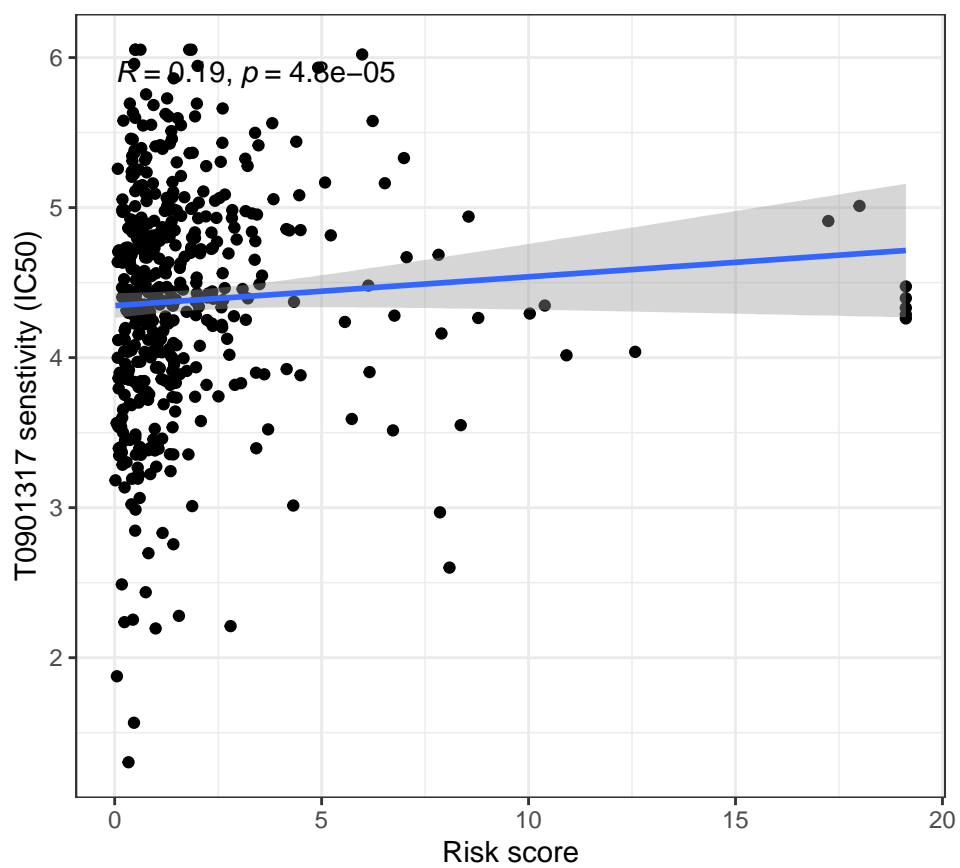

Supplement: Supplementary file 2 — Data S1. [file JCMM-28-e70059-s002.zip › Supplemental Material II/Cor.T0901317.pdf]

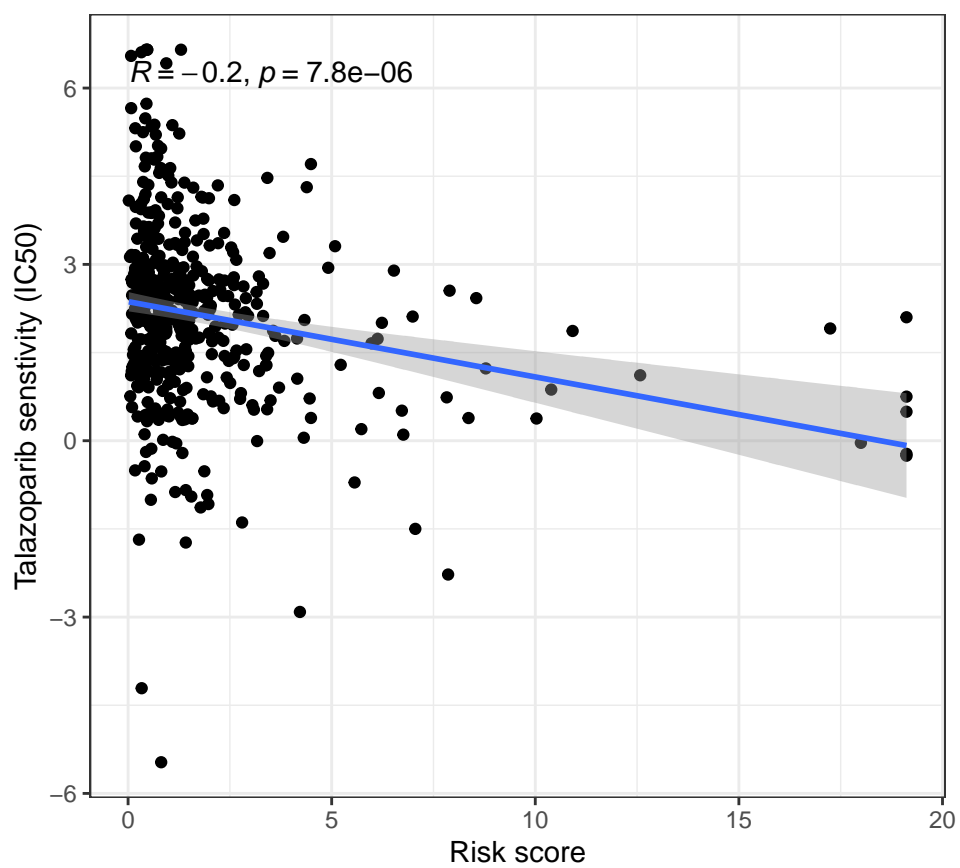

Supplement: Supplementary file 2 — Data S1. [file JCMM-28-e70059-s002.zip › Supplemental Material II/Cor.Talazoparib.pdf]

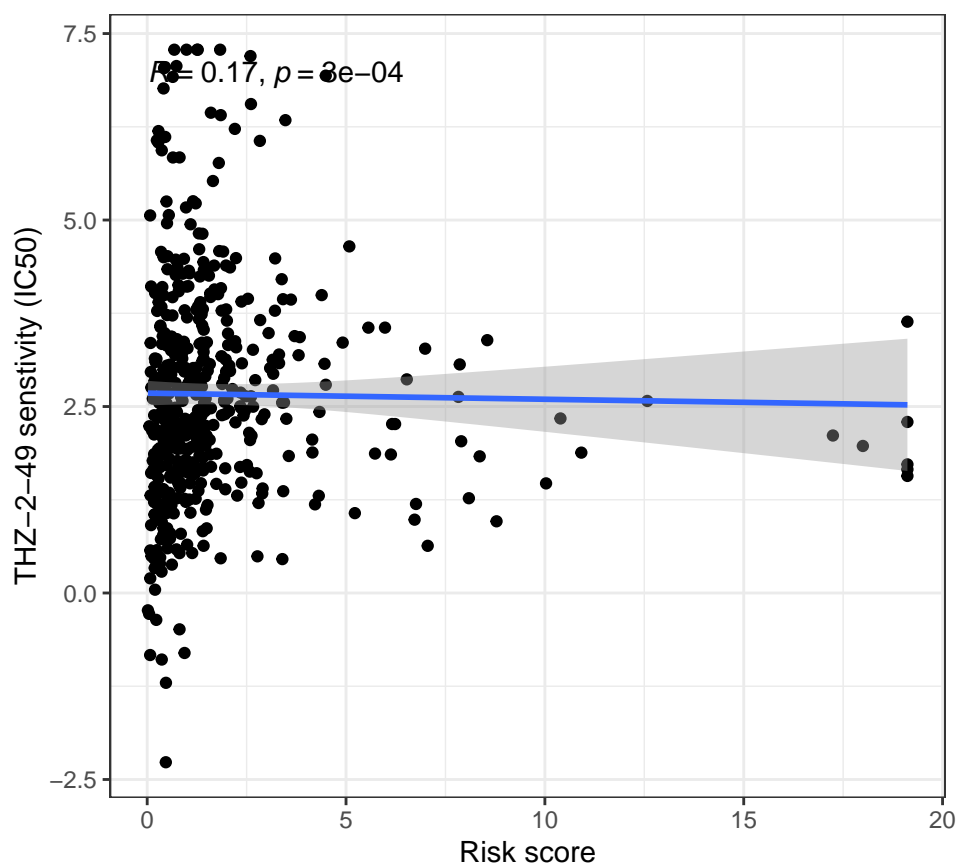

Supplement: Supplementary file 2 — Data S1. [file JCMM-28-e70059-s002.zip › Supplemental Material II/Cor.THZ-2-49.pdf]

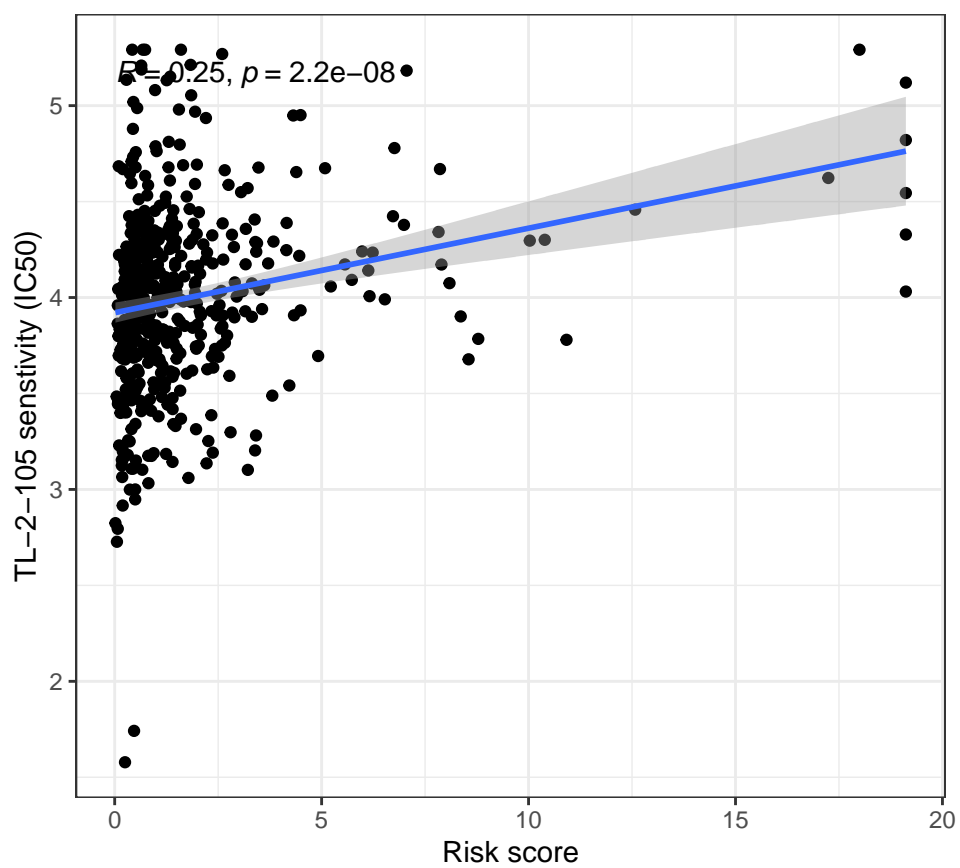

Supplement: Supplementary file 2 — Data S1. [file JCMM-28-e70059-s002.zip › Supplemental Material II/Cor.TL-2-105.pdf]

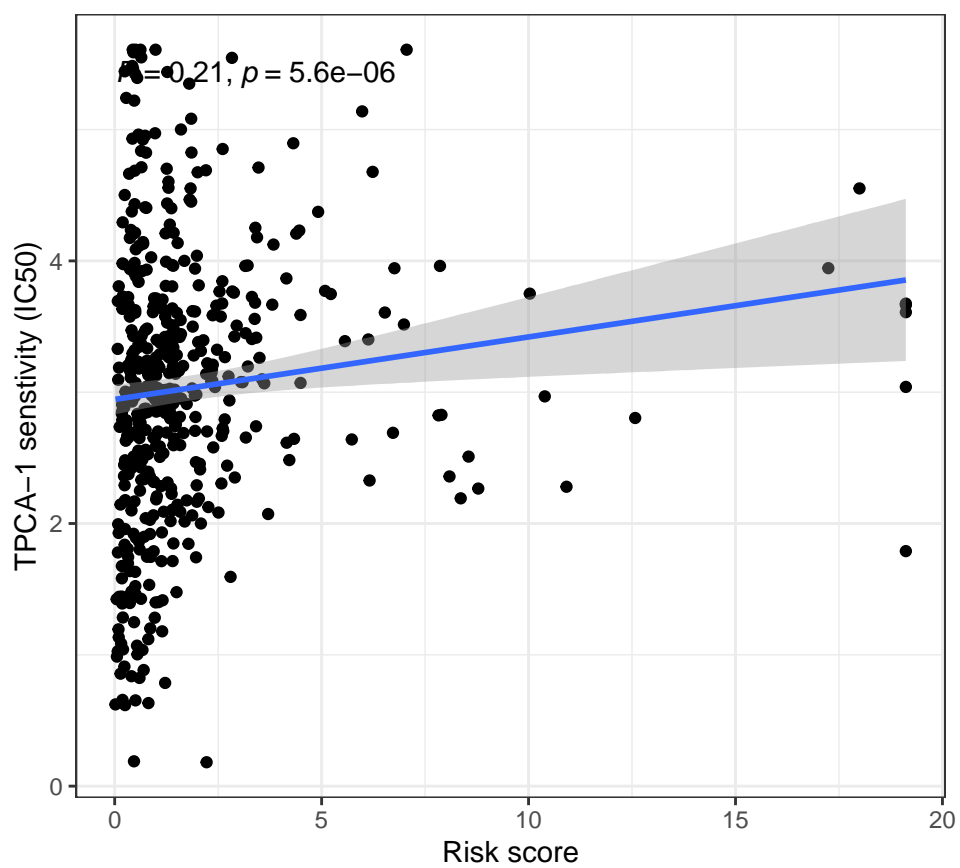

Supplement: Supplementary file 2 — Data S1. [file JCMM-28-e70059-s002.zip › Supplemental Material II/Cor.TPCA-1.pdf]

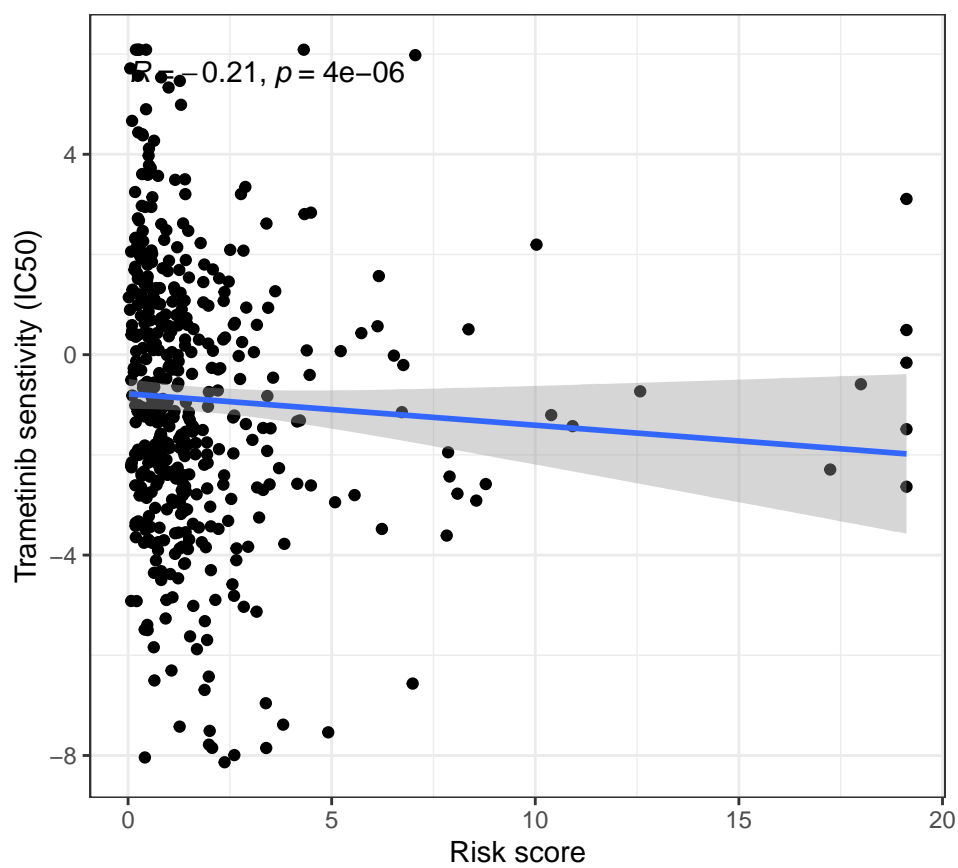

Supplement: Supplementary file 2 — Data S1. [file JCMM-28-e70059-s002.zip › Supplemental Material II/Cor.Trametinib.pdf]

TW 37 sensitivity (IC50)

$R = -0.18, p = 5.5e-05$

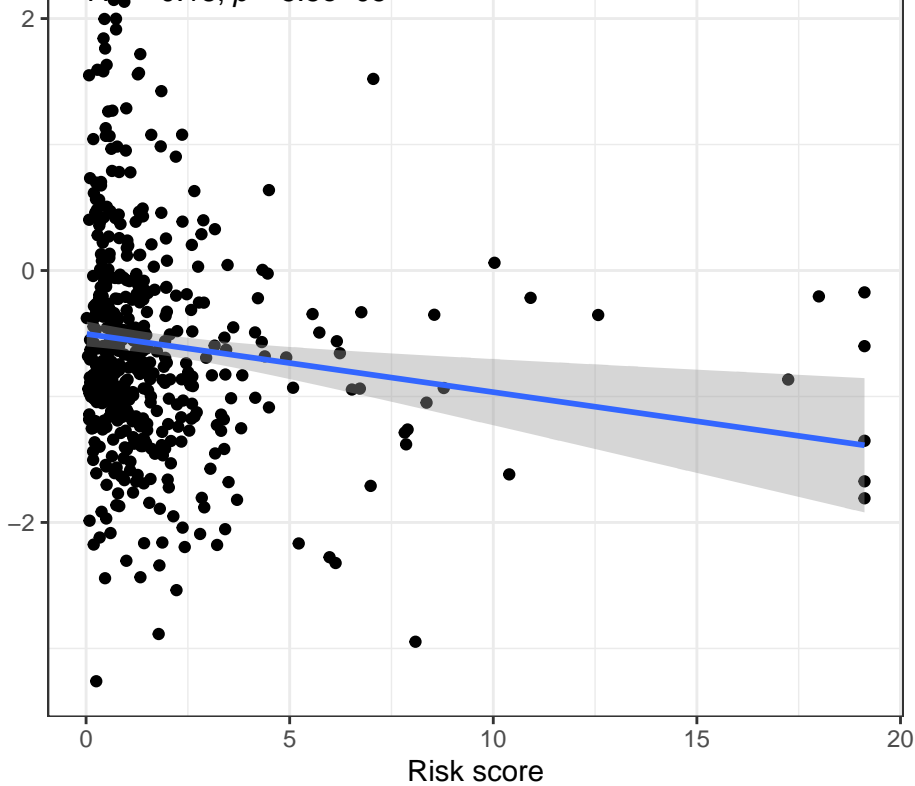

Supplement: Supplementary file 2 — Data S1. [file JCMM-28-e70059-s002.zip › Supplemental Material II/Cor.TW 37.pdf]

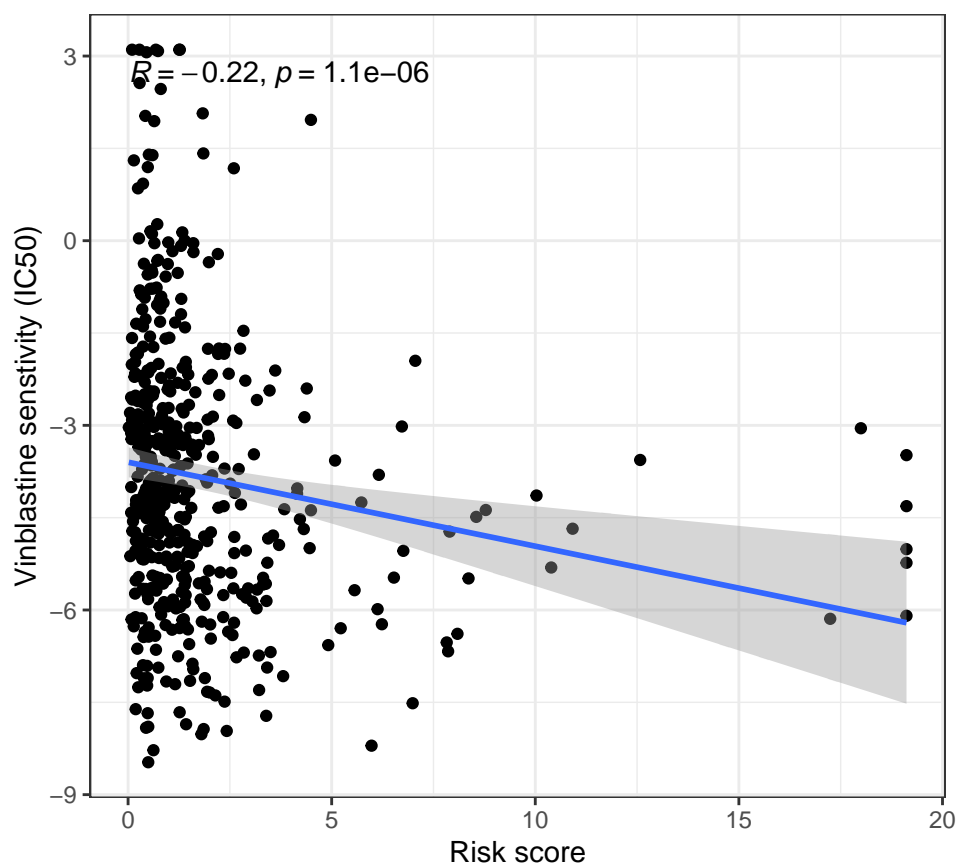

Supplement: Supplementary file 2 — Data S1. [file JCMM-28-e70059-s002.zip › Supplemental Material II/Cor.Vinblastine.pdf]

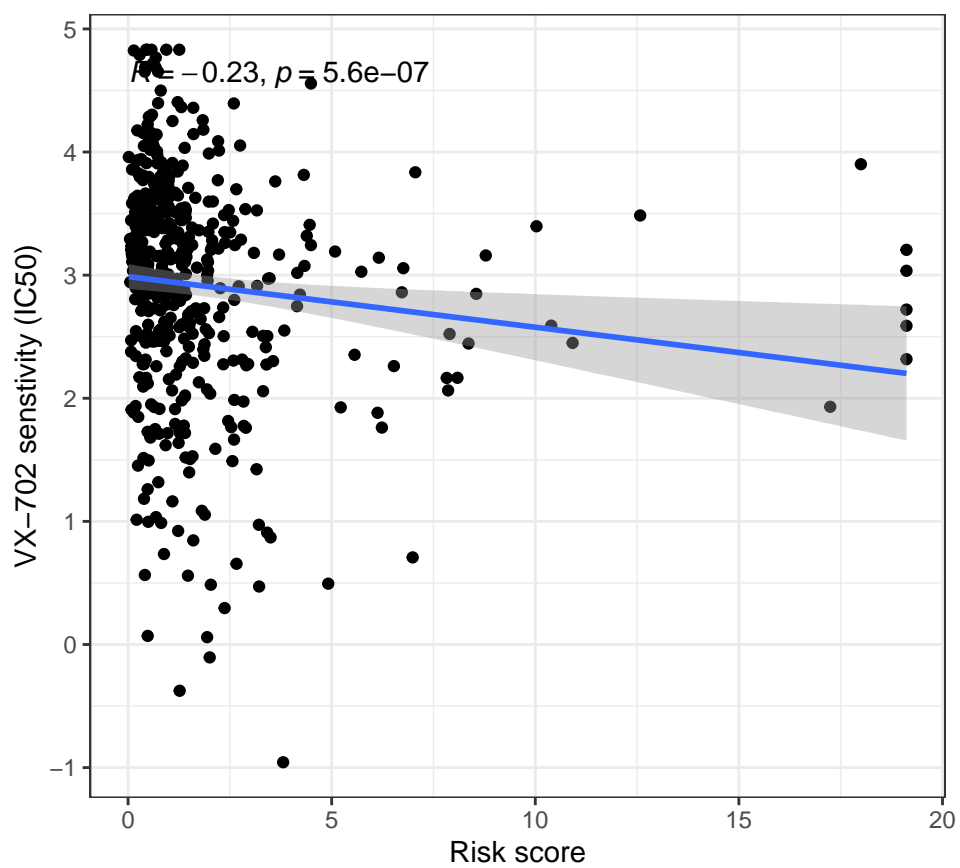

Supplement: Supplementary file 2 — Data S1. [file JCMM-28-e70059-s002.zip › Supplemental Material II/Cor.VX-702.pdf]

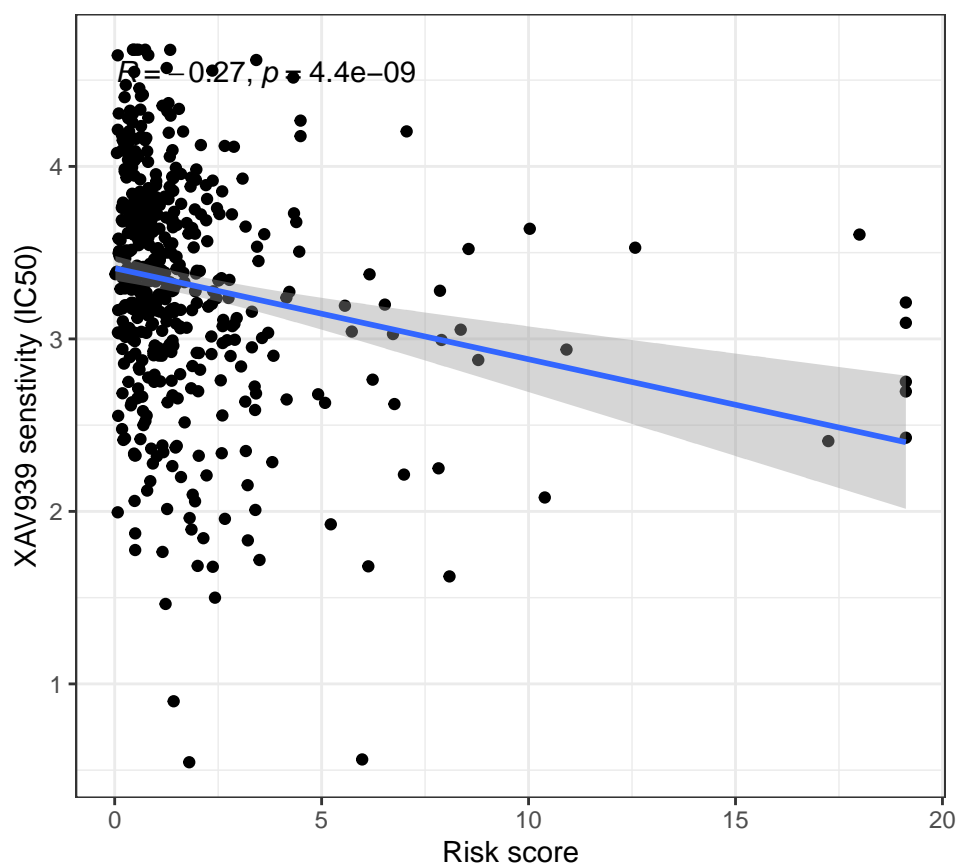

Supplement: Supplementary file 2 — Data S1. [file JCMM-28-e70059-s002.zip › Supplemental Material II/Cor.XAV939.pdf]

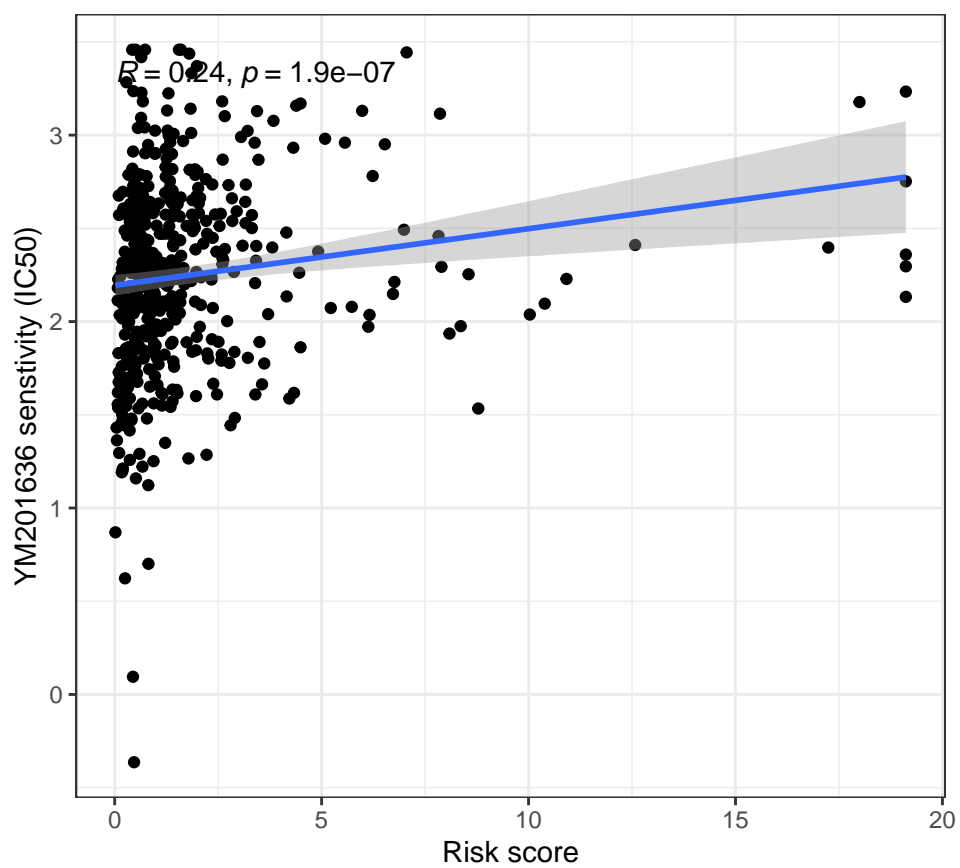

Supplement: Supplementary file 2 — Data S1. [file JCMM-28-e70059-s002.zip › Supplemental Material II/Cor.YM201636.pdf]

Risk 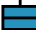 low 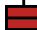 high

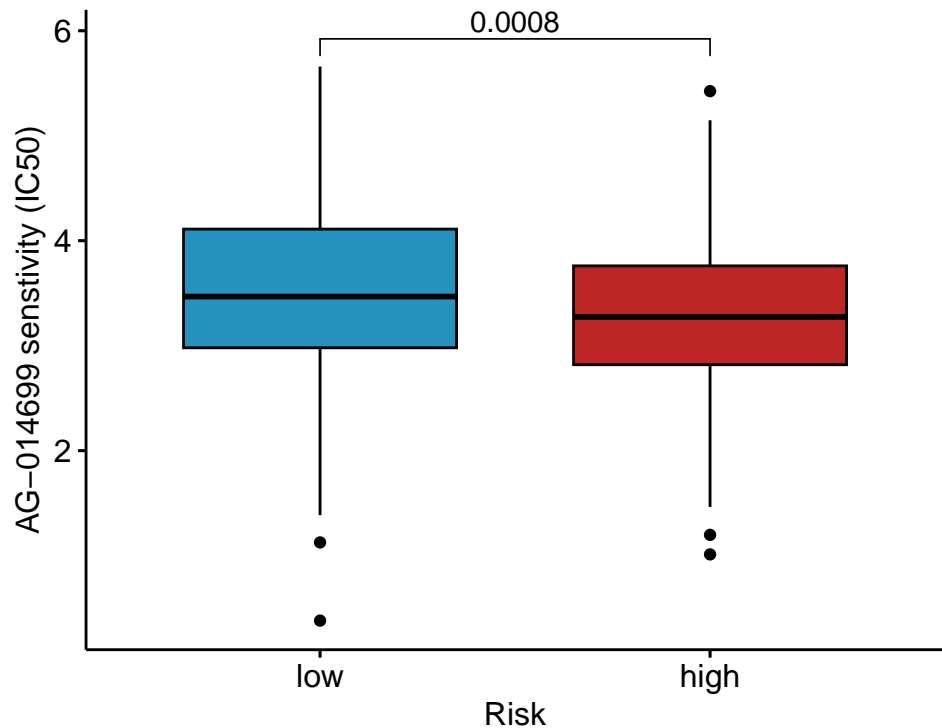

Supplement: Supplementary file 2 — Data S1. [file JCMM-28-e70059-s002.zip › Supplemental Material II/durgSenstivity.AG-014699.pdf]

Risk 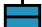 low 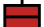 high

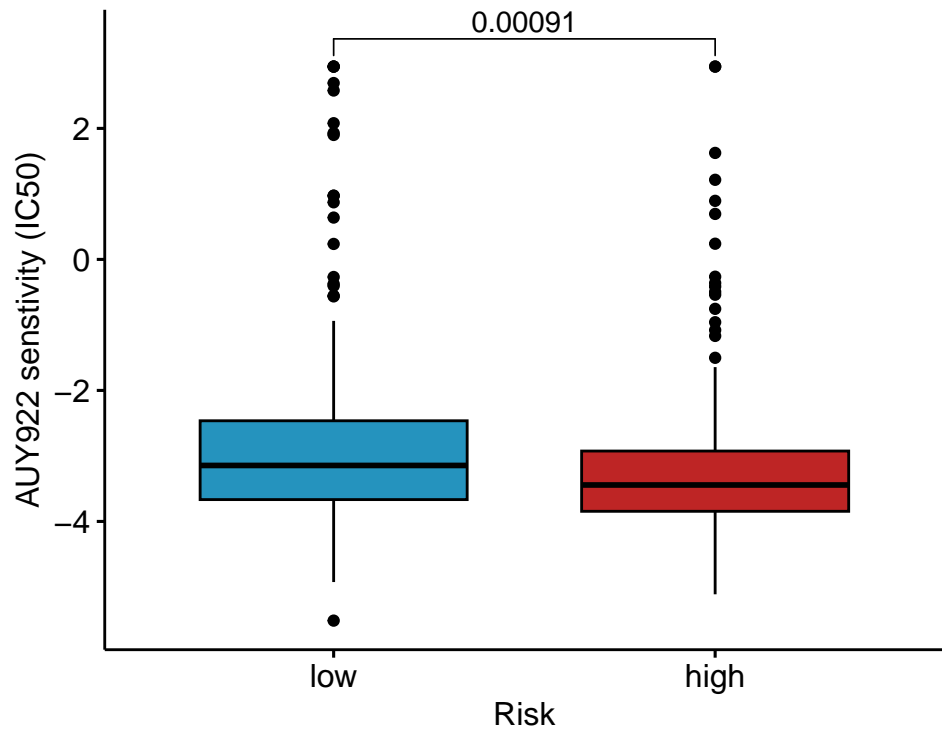

Supplement: Supplementary file 2 — Data S1. [file JCMM-28-e70059-s002.zip › Supplemental Material II/durgSenstivity.AUY922.pdf]

Risk 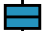 low 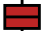 high

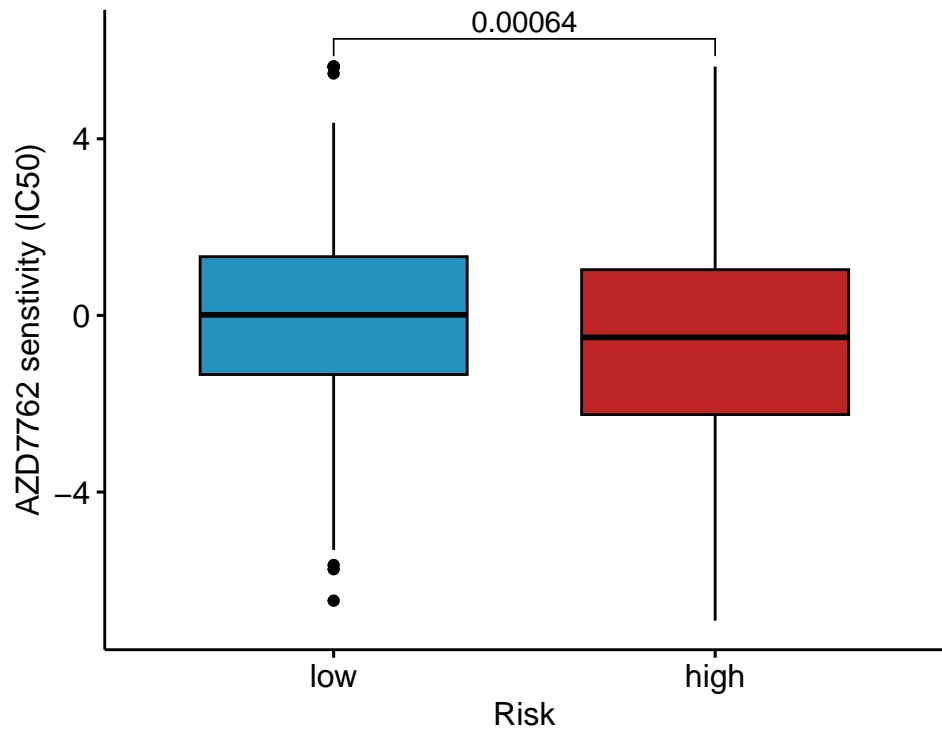

Supplement: Supplementary file 2 — Data S1. [file JCMM-28-e70059-s002.zip › Supplemental Material II/durgSenstivity.AZD7762.pdf]

Risk low high

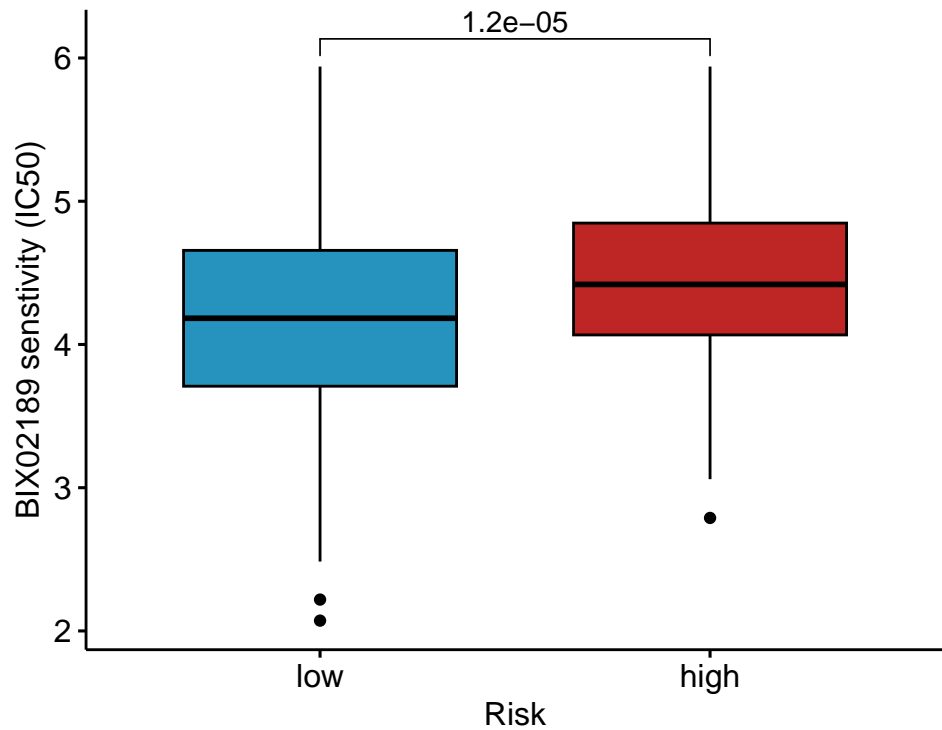

Supplement: Supplementary file 2 — Data S1. [file JCMM-28-e70059-s002.zip › Supplemental Material II/durgSenstivity.BIX02189.pdf]

Risk 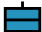 low 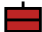 high

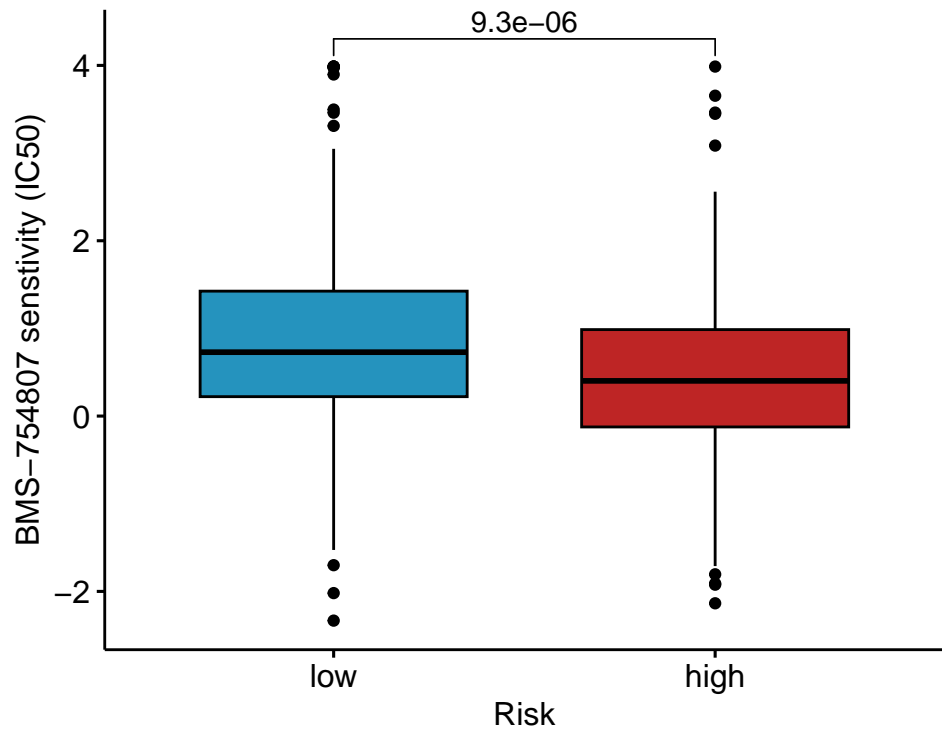

Supplement: Supplementary file 2 — Data S1. [file JCMM-28-e70059-s002.zip › Supplemental Material II/durgSenstivity.BMS-754807.pdf]

Risk 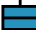 low 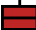 high

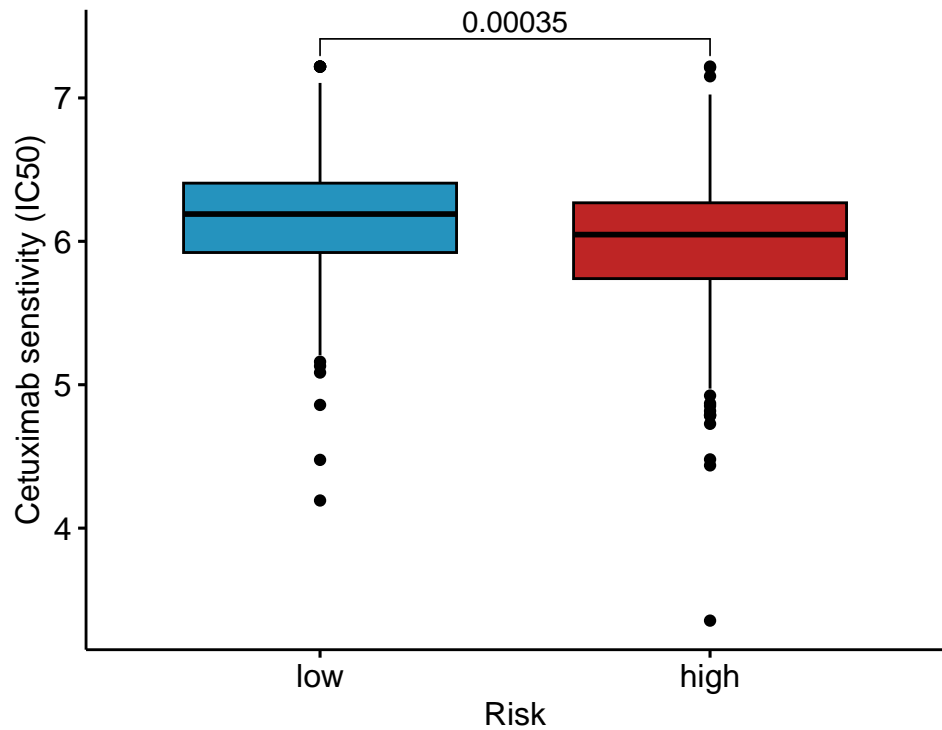

Supplement: Supplementary file 2 — Data S1. [file JCMM-28-e70059-s002.zip › Supplemental Material II/durgSenstivity.Cetuximab.pdf]

Risk 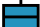 low 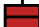 high

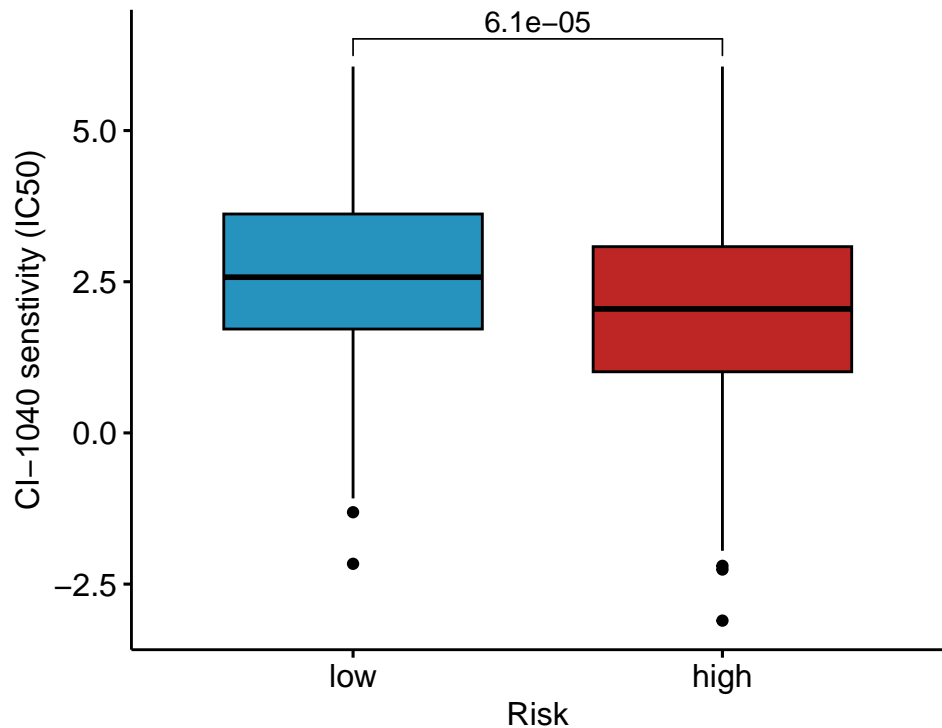

Supplement: Supplementary file 2 — Data S1. [file JCMM-28-e70059-s002.zip › Supplemental Material II/durgSenstivity.CI-1040.pdf]

Risk 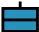 low 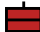 high

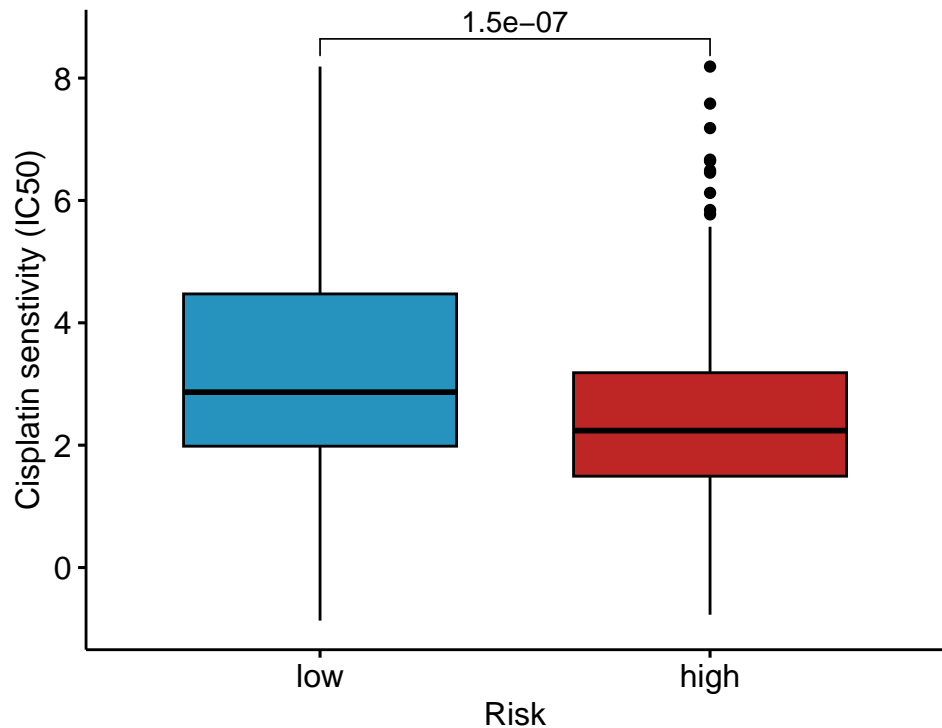

Supplement: Supplementary file 2 — Data S1. [file JCMM-28-e70059-s002.zip › Supplemental Material II/durgSenstivity.Cisplatin.pdf]

Risk 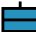 low 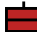 high

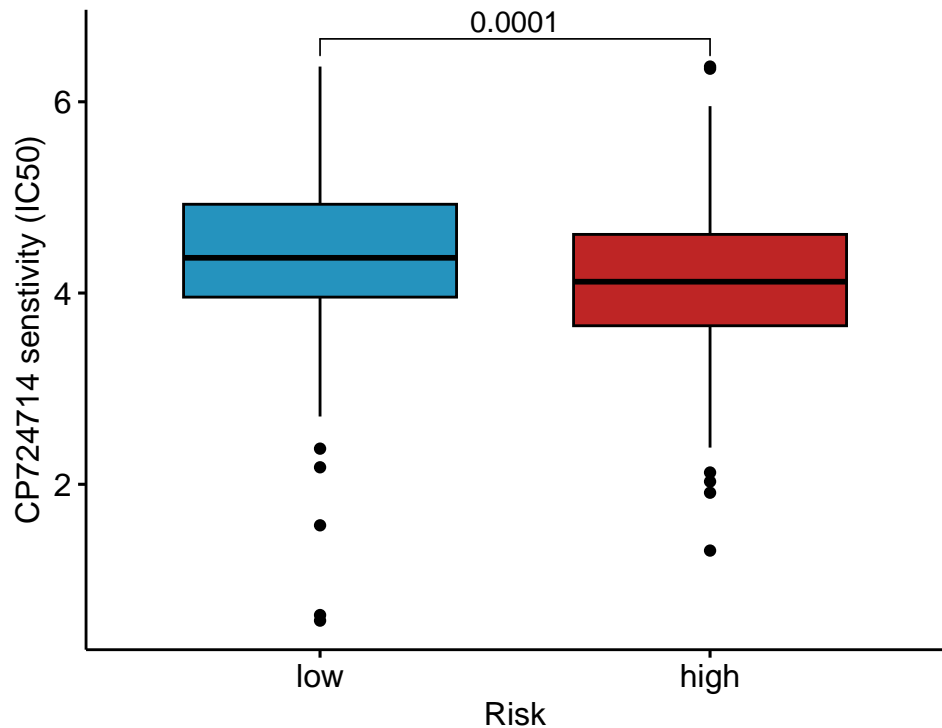

Supplement: Supplementary file 2 — Data S1. [file JCMM-28-e70059-s002.zip › Supplemental Material II/durgSenstivity.CP724714.pdf]

Risk 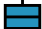 low 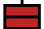 high

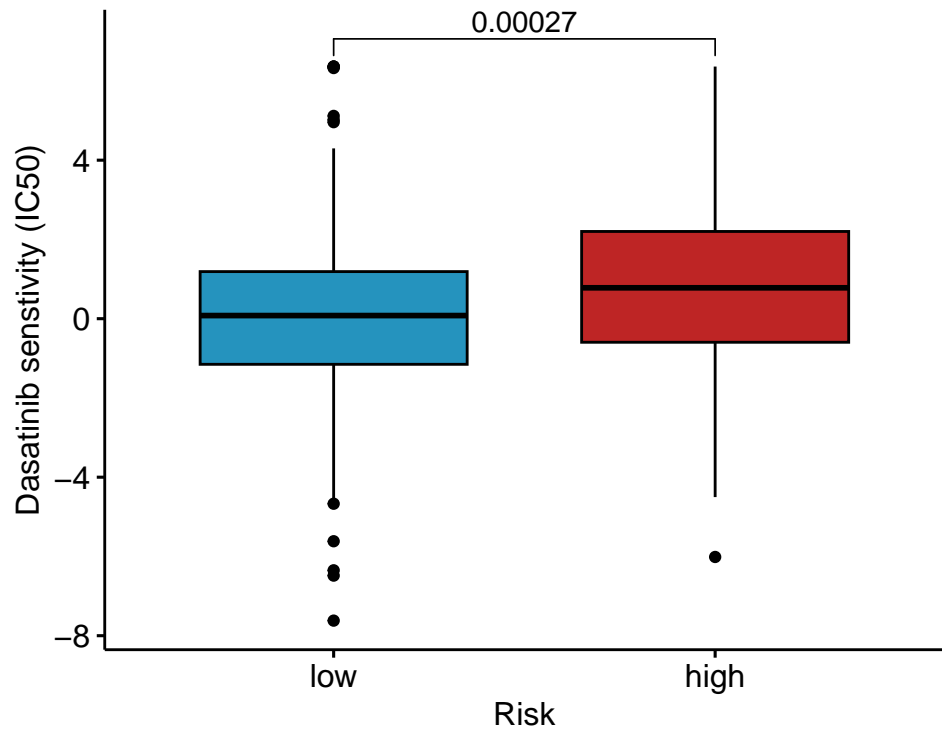

Supplement: Supplementary file 2 — Data S1. [file JCMM-28-e70059-s002.zip › Supplemental Material II/durgSenstivity.Dasatinib.pdf]

Risk 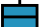 low 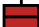 high

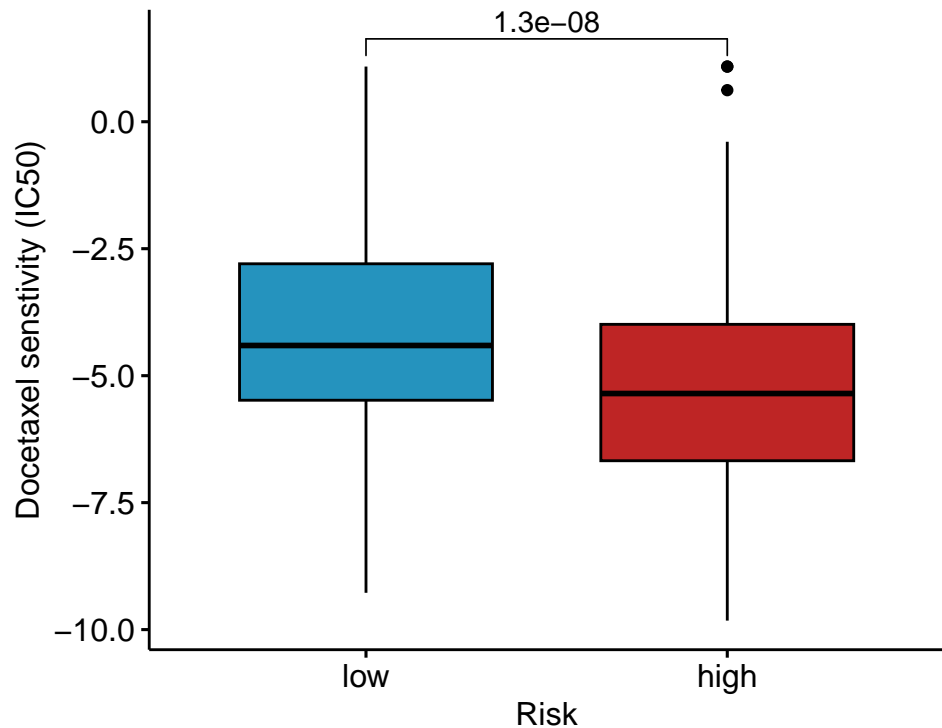

Supplement: Supplementary file 2 — Data S1. [file JCMM-28-e70059-s002.zip › Supplemental Material II/durgSenstivity.Docetaxel.pdf]

Risk 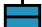 low 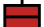 high

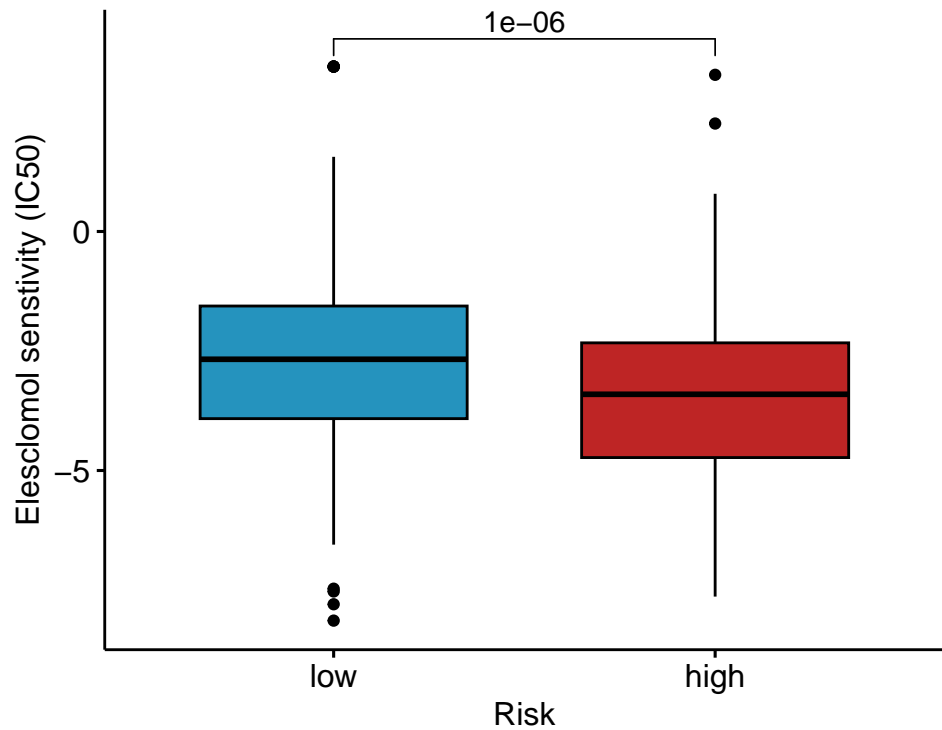

Supplement: Supplementary file 2 — Data S1. [file JCMM-28-e70059-s002.zip › Supplemental Material II/durgSenstivity.Elesclomol.pdf]

Risk 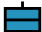 low 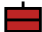 high

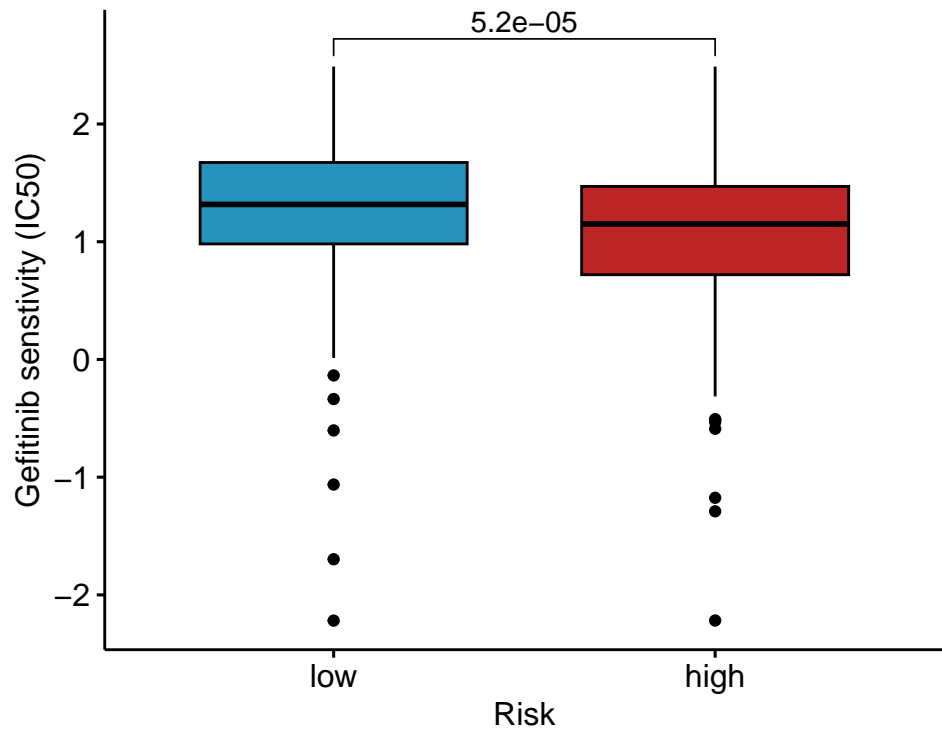

Supplement: Supplementary file 2 — Data S1. [file JCMM-28-e70059-s002.zip › Supplemental Material II/durgSenstivity.Gefitinib.pdf]

Risk 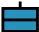 low 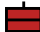 high

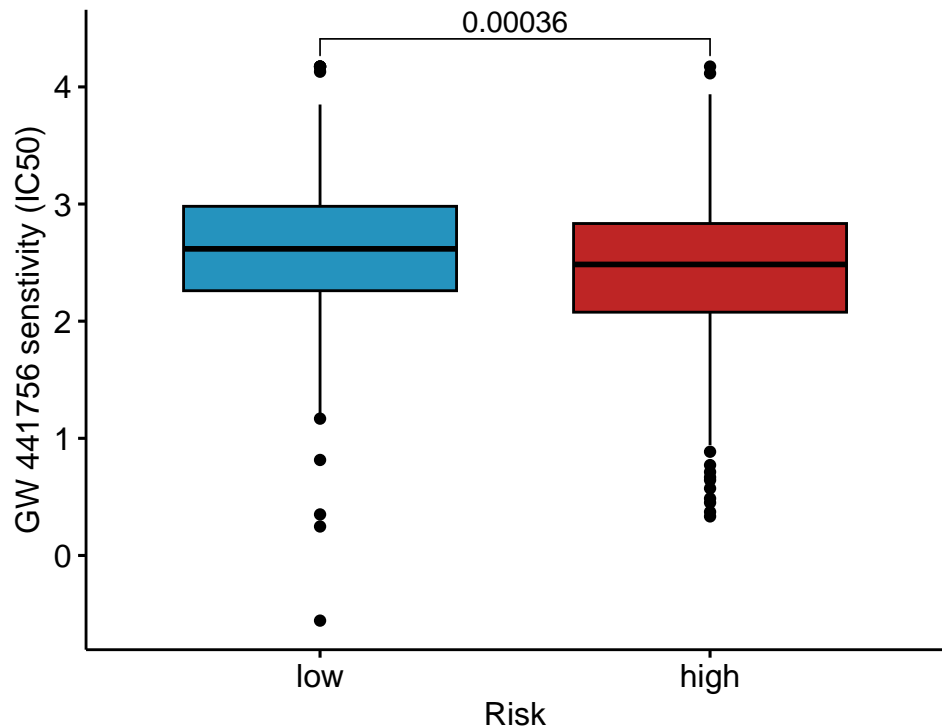

Supplement: Supplementary file 2 — Data S1. [file JCMM-28-e70059-s002.zip › Supplemental Material II/durgSenstivity.GW 441756.pdf]

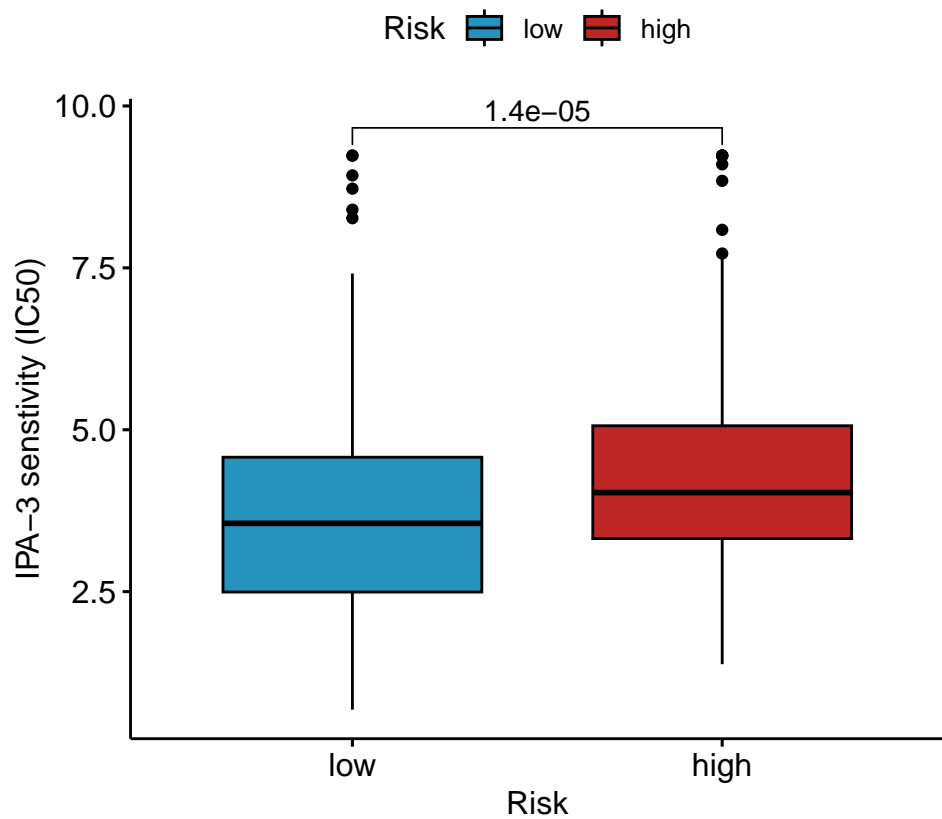

Supplement: Supplementary file 2 — Data S1. [file JCMM-28-e70059-s002.zip › Supplemental Material II/durgSenstivity.IPA-3.pdf]

Risk 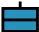 low 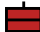 high

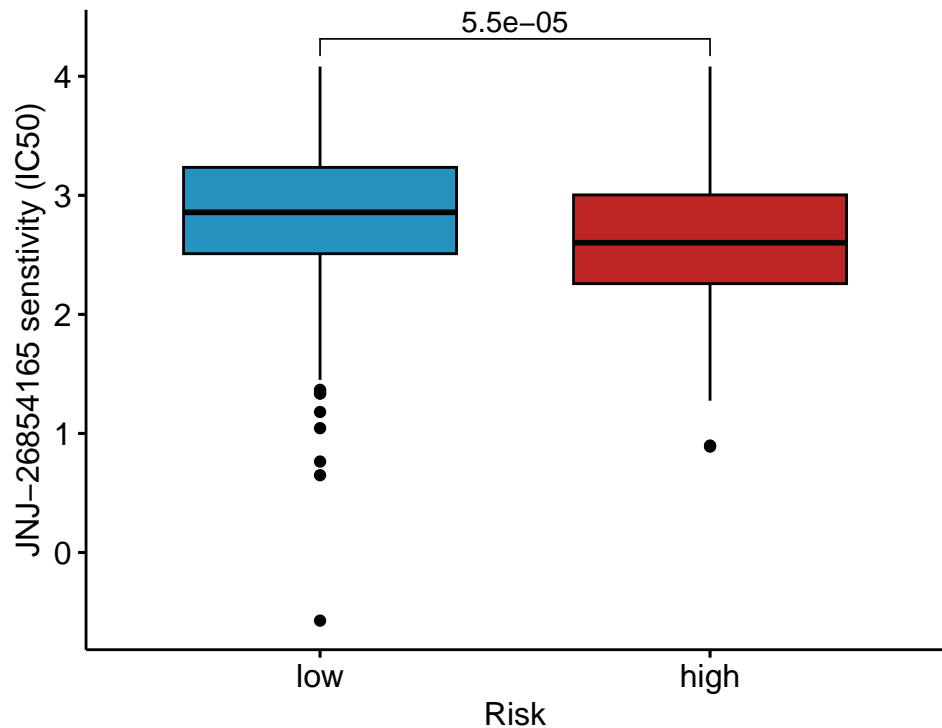

Supplement: Supplementary file 2 — Data S1. [file JCMM-28-e70059-s002.zip › Supplemental Material II/durgSenstivity.JNJ-26854165.pdf]

Risk 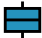 low 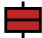 high

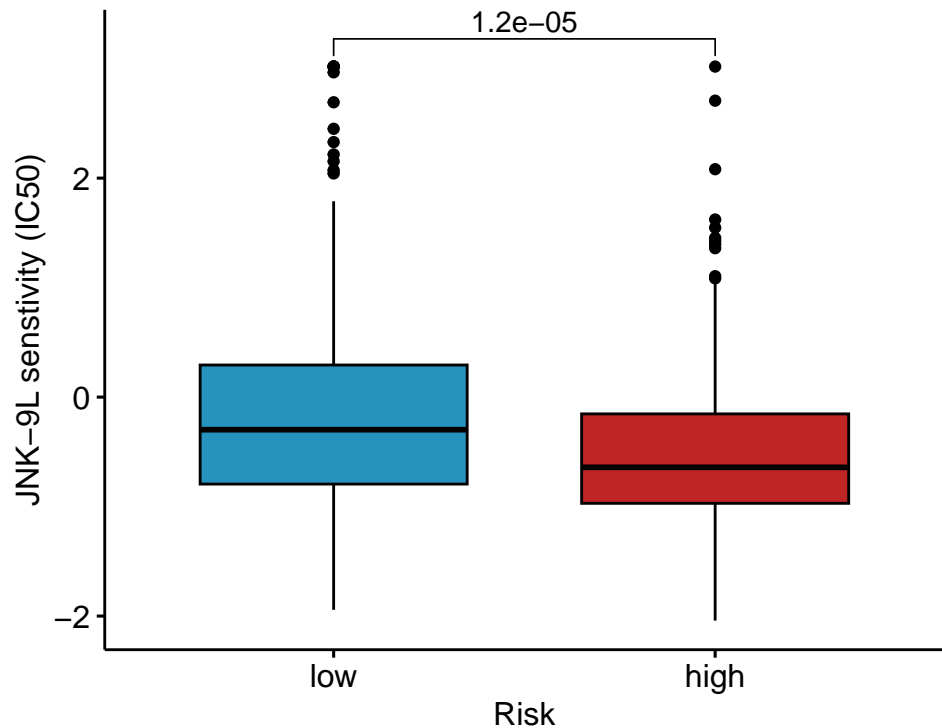

Supplement: Supplementary file 2 — Data S1. [file JCMM-28-e70059-s002.zip › Supplemental Material II/durgSenstivity.JNK-9L.pdf]

Risk 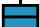 low 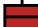 high

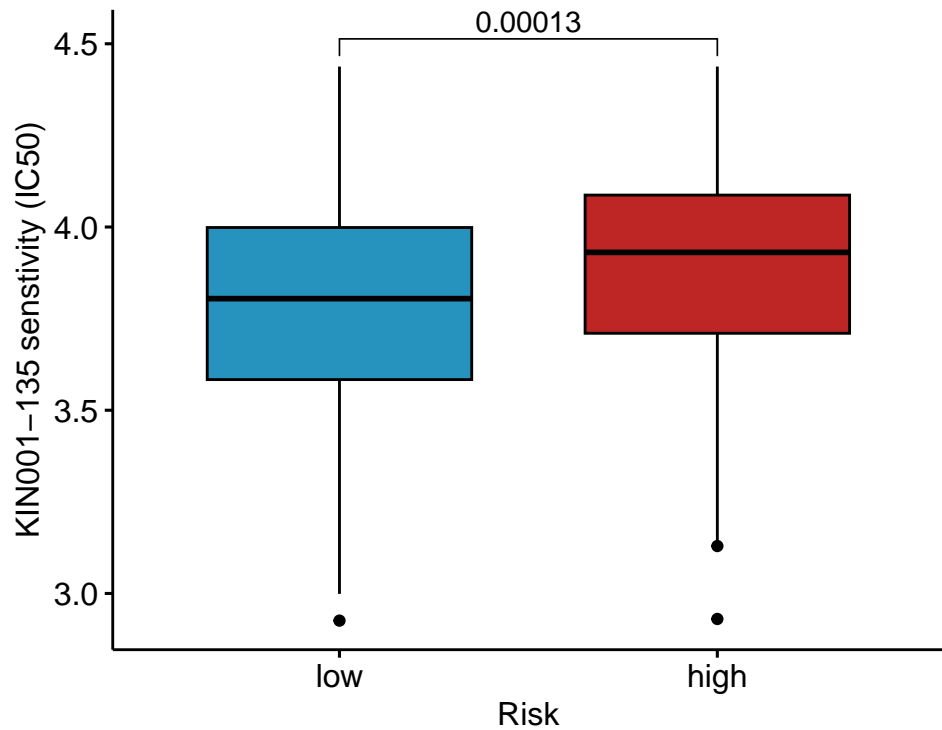

Supplement: Supplementary file 2 — Data S1. [file JCMM-28-e70059-s002.zip › Supplemental Material II/durgSenstivity.KIN001-135.pdf]

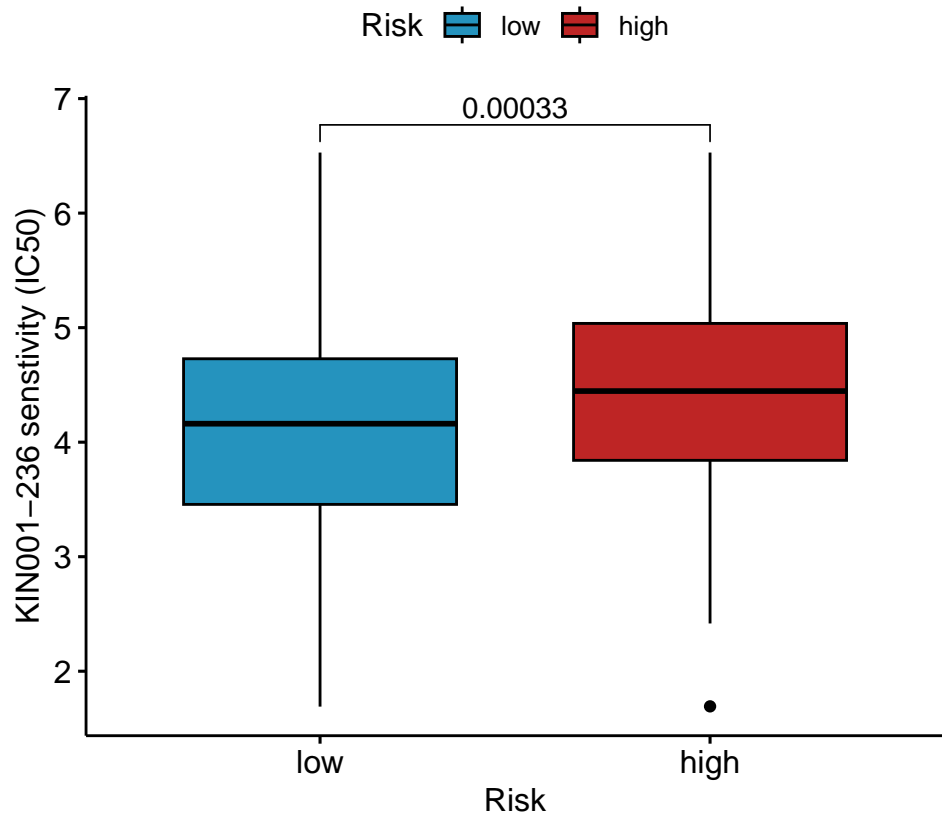

Supplement: Supplementary file 2 — Data S1. [file JCMM-28-e70059-s002.zip › Supplemental Material II/durgSenstivity.KIN001-236.pdf]

Risk low high

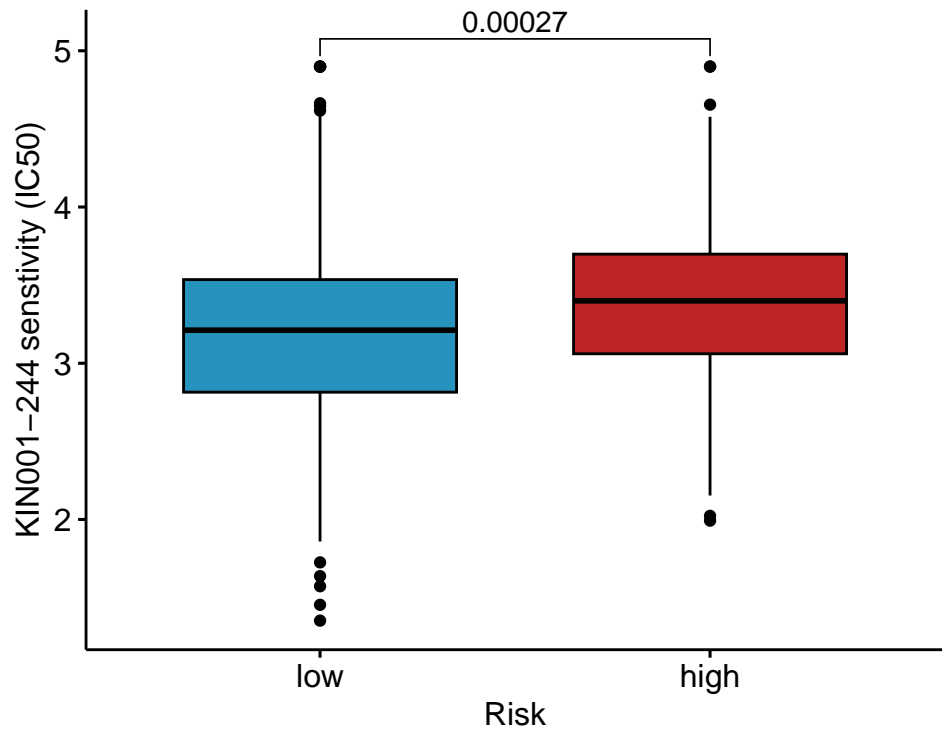

Supplement: Supplementary file 2 — Data S1. [file JCMM-28-e70059-s002.zip › Supplemental Material II/durgSenstivity.KIN001-244.pdf]

Risk low high

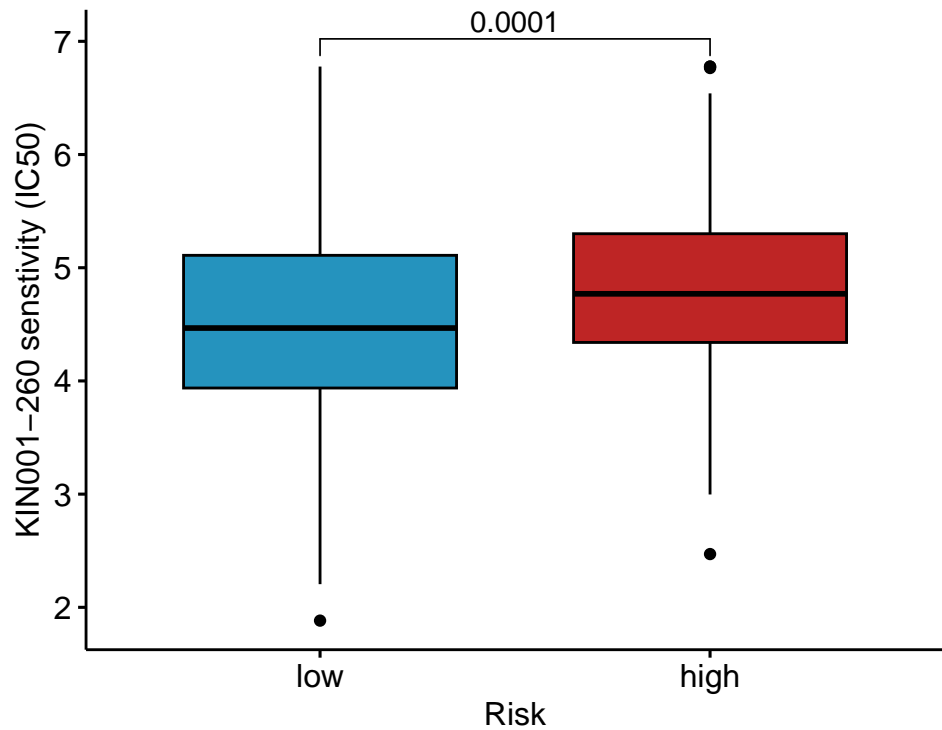

Supplement: Supplementary file 2 — Data S1. [file JCMM-28-e70059-s002.zip › Supplemental Material II/durgSenstivity.KIN001-260.pdf]

Risk 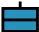 low 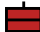 high

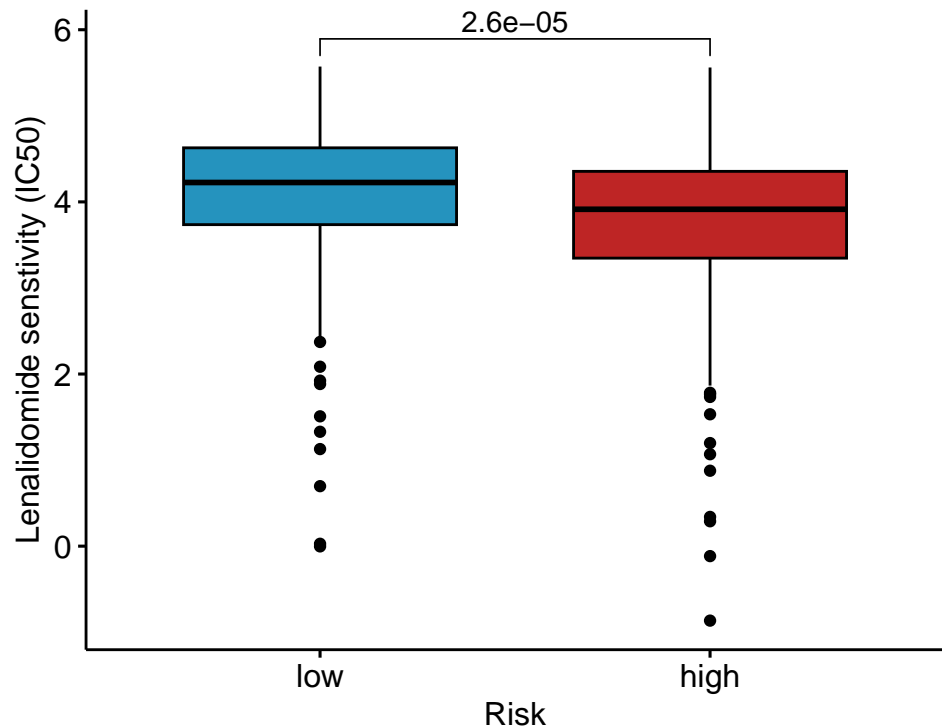

Supplement: Supplementary file 2 — Data S1. [file JCMM-28-e70059-s002.zip › Supplemental Material II/durgSenstivity.Lenalidomide.pdf]

Risk 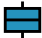 low 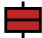 high

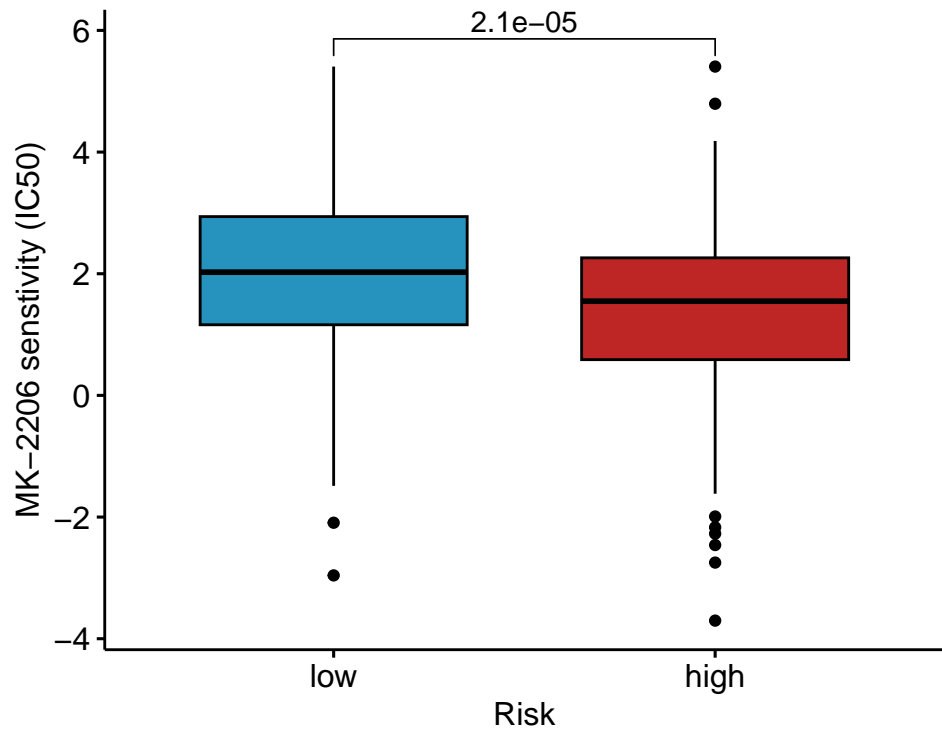

Supplement: Supplementary file 2 — Data S1. [file JCMM-28-e70059-s002.zip › Supplemental Material II/durgSenstivity.MK-2206.pdf]

Risk 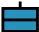 low 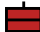 high

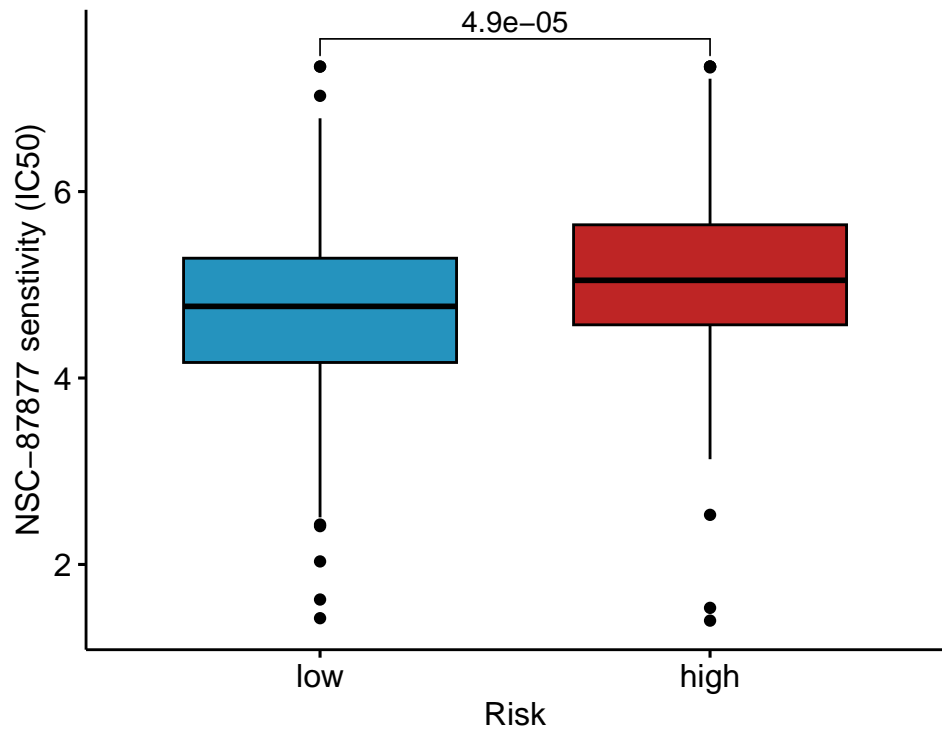

Supplement: Supplementary file 2 — Data S1. [file JCMM-28-e70059-s002.zip › Supplemental Material II/durgSenstivity.NSC-87877.pdf]

Risk 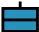 low 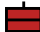 high

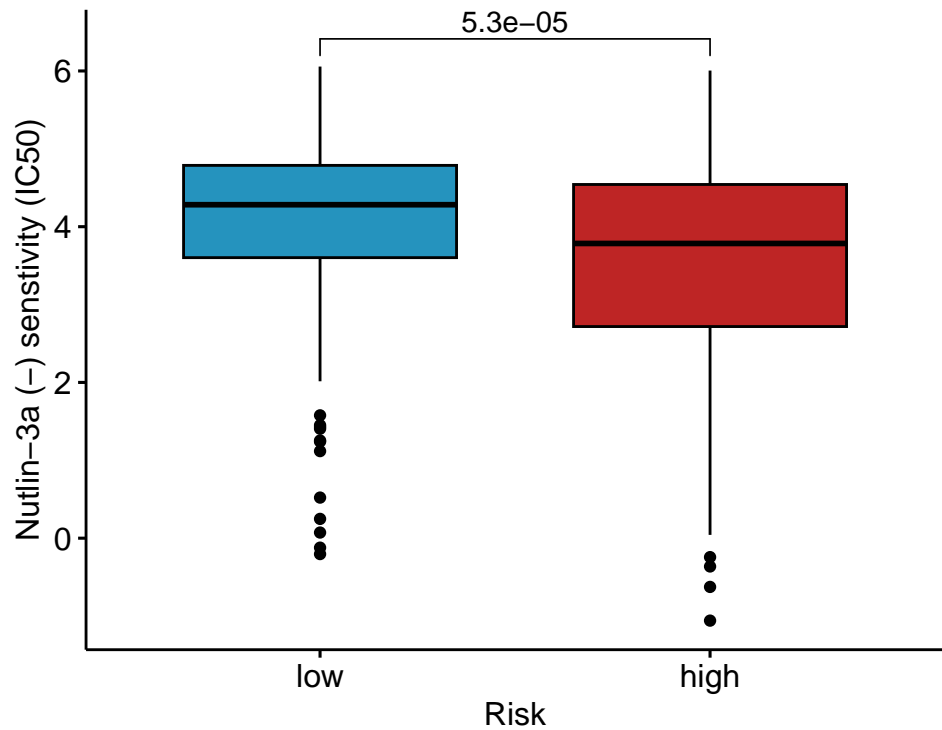

Supplement: Supplementary file 2 — Data S1. [file JCMM-28-e70059-s002.zip › Supplemental Material II/durgSenstivity.Nutlin-3a (-).pdf]

Risk 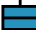 low 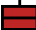 high

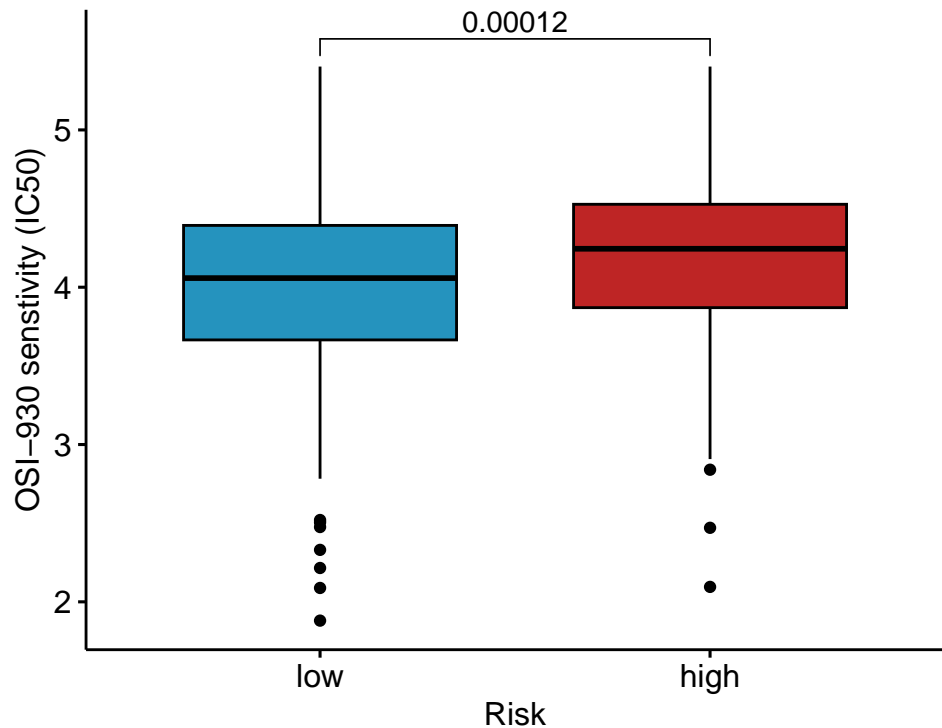

Supplement: Supplementary file 2 — Data S1. [file JCMM-28-e70059-s002.zip › Supplemental Material II/durgSenstivity.OSI-930.pdf]

Risk 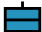 low 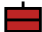 high

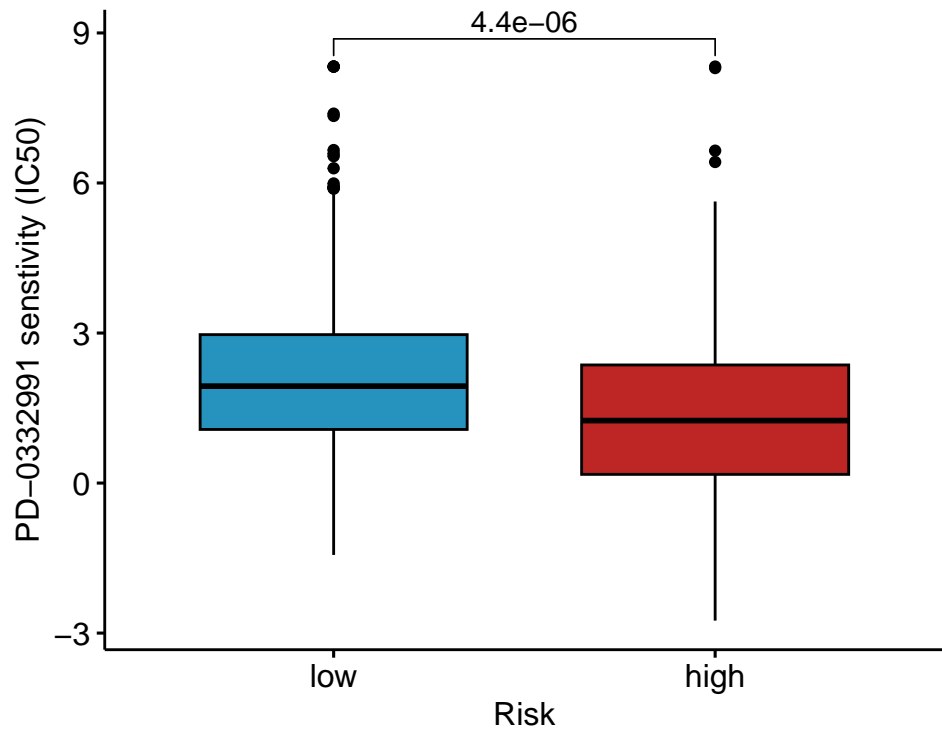

Supplement: Supplementary file 2 — Data S1. [file JCMM-28-e70059-s002.zip › Supplemental Material II/durgSenstivity.PD-0332991.pdf]

Risk 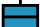 low 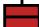 high

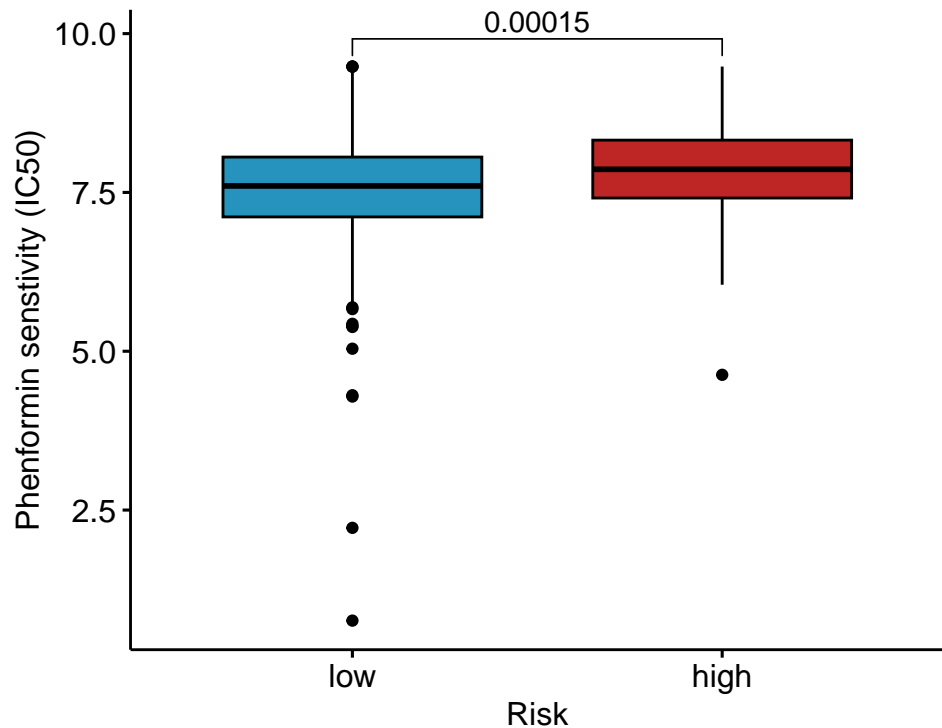

Supplement: Supplementary file 2 — Data S1. [file JCMM-28-e70059-s002.zip › Supplemental Material II/durgSenstivity.Phenformin.pdf]

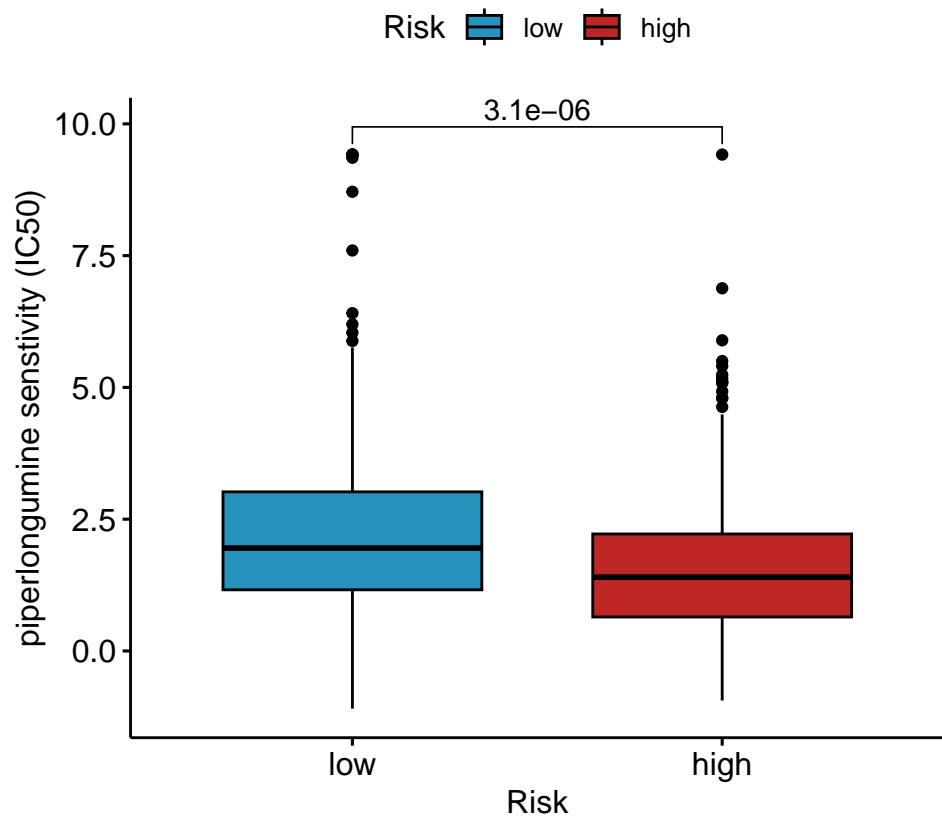

Supplement: Supplementary file 2 — Data S1. [file JCMM-28-e70059-s002.zip › Supplemental Material II/durgSenstivity.piperlongumine.pdf]

Risk 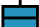 low 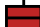 high

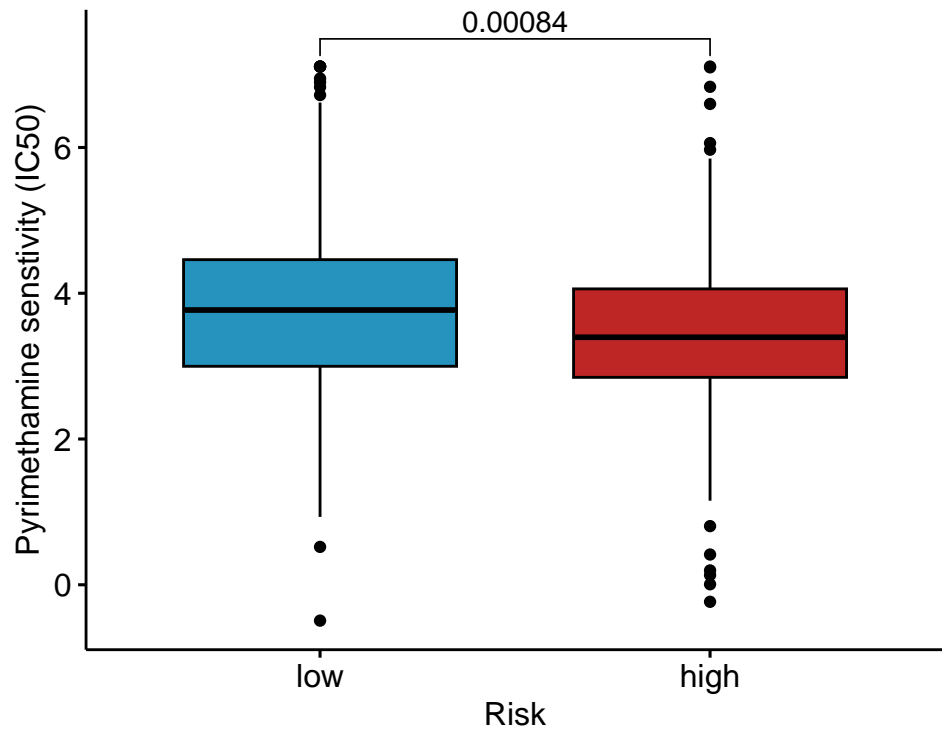

Supplement: Supplementary file 2 — Data S1. [file JCMM-28-e70059-s002.zip › Supplemental Material II/durgSenstivity.Pyrimethamine.pdf]

Risk 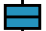 low 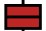 high

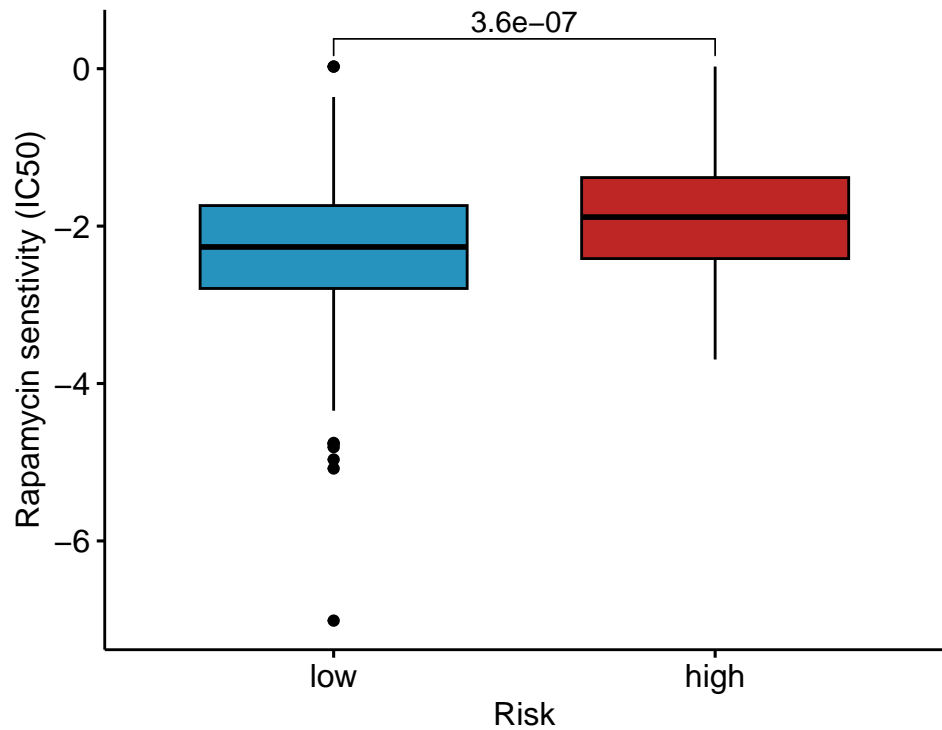

Supplement: Supplementary file 2 — Data S1. [file JCMM-28-e70059-s002.zip › Supplemental Material II/durgSenstivity.Rapamycin.pdf]

Risk 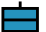 low 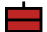 high

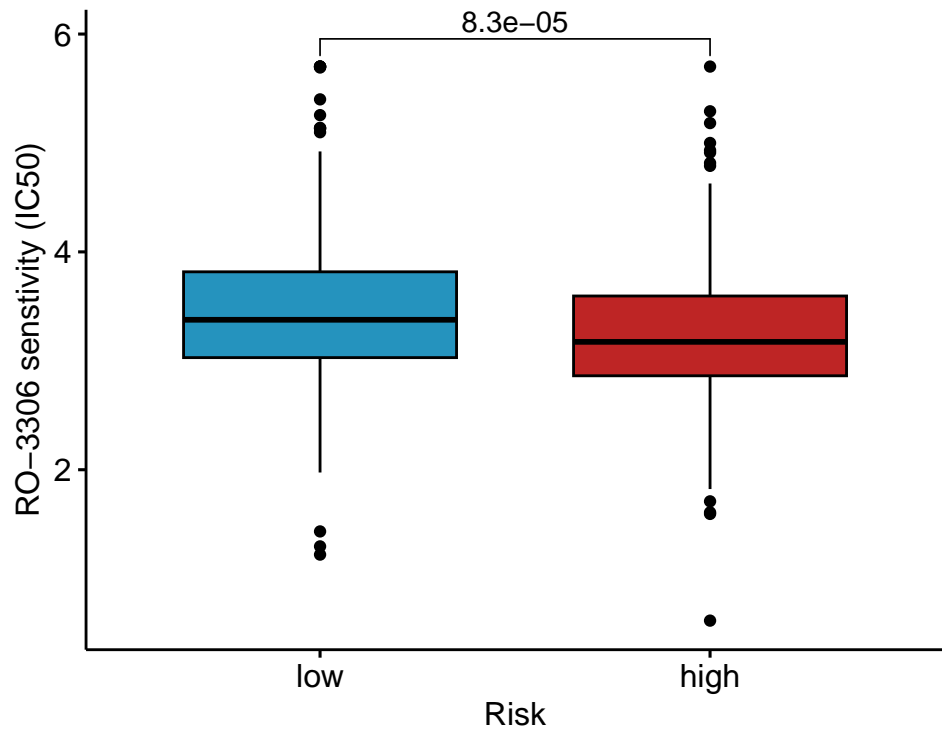

Supplement: Supplementary file 2 — Data S1. [file JCMM-28-e70059-s002.zip › Supplemental Material II/durgSenstivity.RO-3306.pdf]

Risk 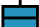 low 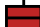 high

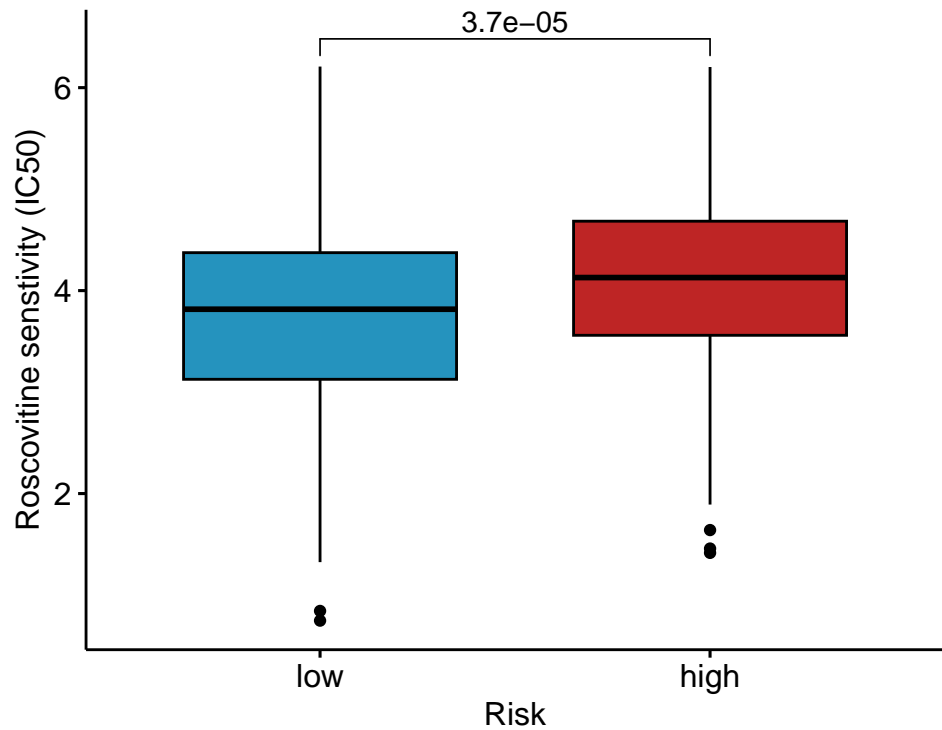

Supplement: Supplementary file 2 — Data S1. [file JCMM-28-e70059-s002.zip › Supplemental Material II/durgSenstivity.Roscovitine.pdf]

Risk 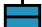 low 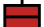 high

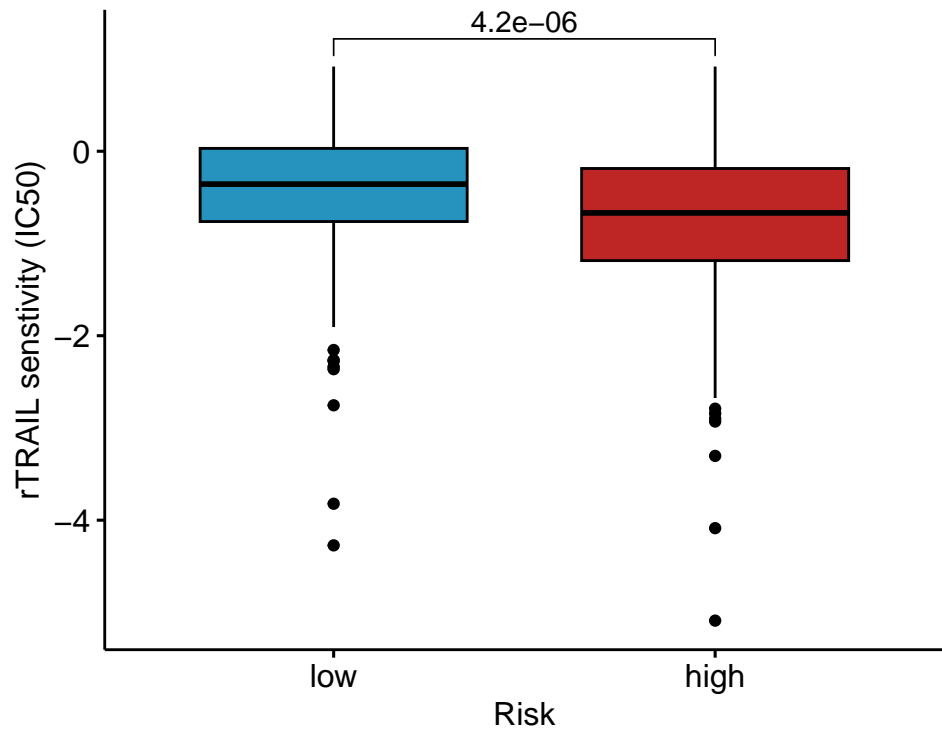

Supplement: Supplementary file 2 — Data S1. [file JCMM-28-e70059-s002.zip › Supplemental Material II/durgSenstivity.rTRAIL.pdf]

Risk 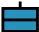 low 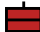 high

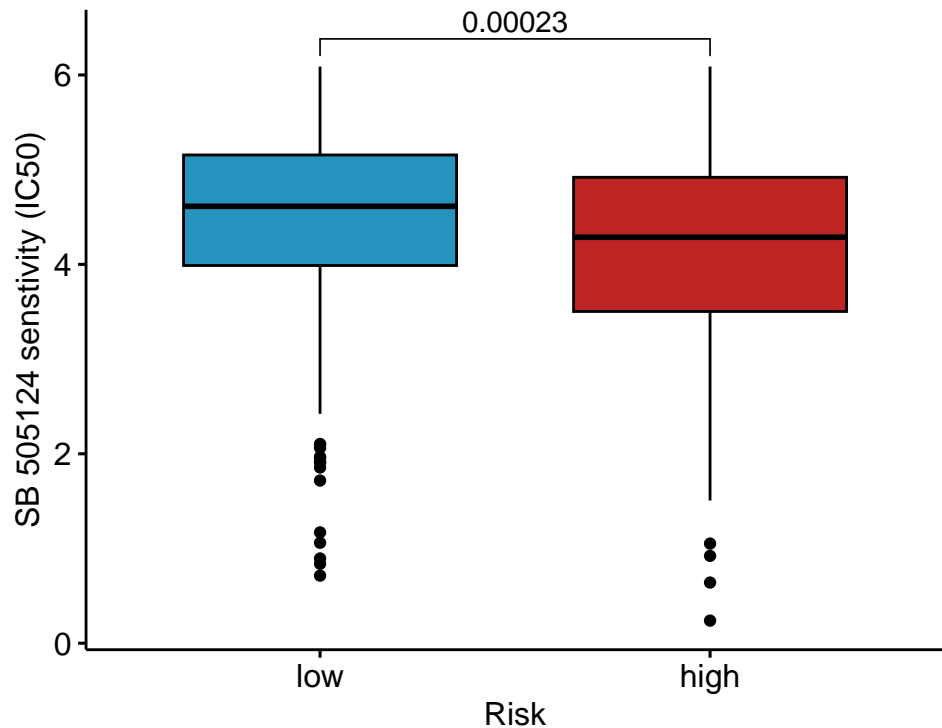

Supplement: Supplementary file 2 — Data S1. [file JCMM-28-e70059-s002.zip › Supplemental Material II/durgSenstivity.SB 505124.pdf]

Risk low high

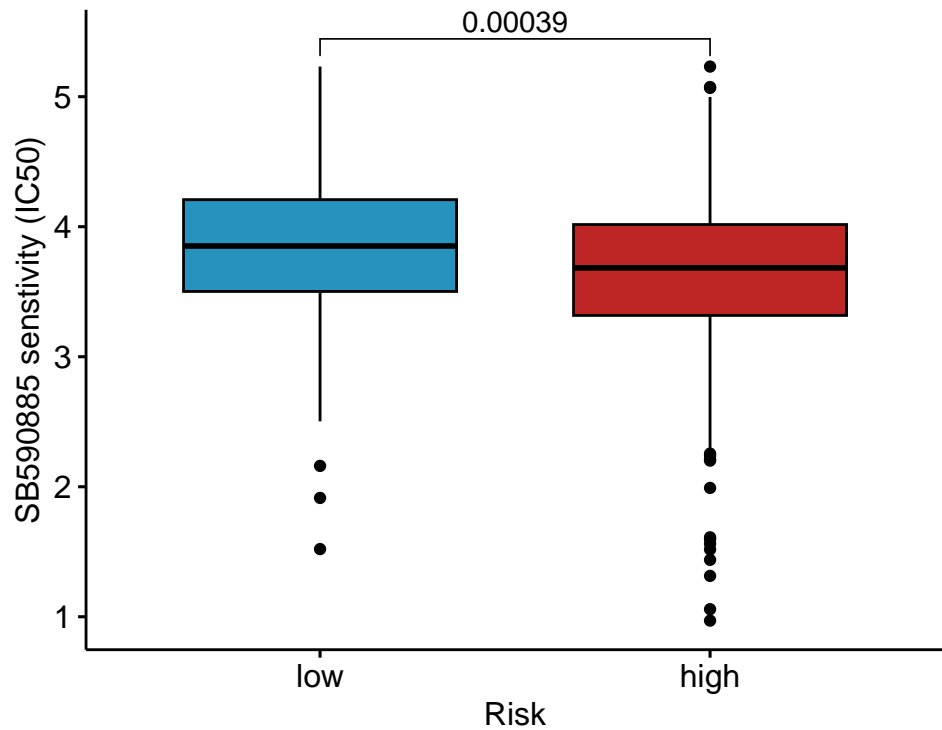

Supplement: Supplementary file 2 — Data S1. [file JCMM-28-e70059-s002.zip › Supplemental Material II/durgSenstivity.SB590885.pdf]

Risk low high

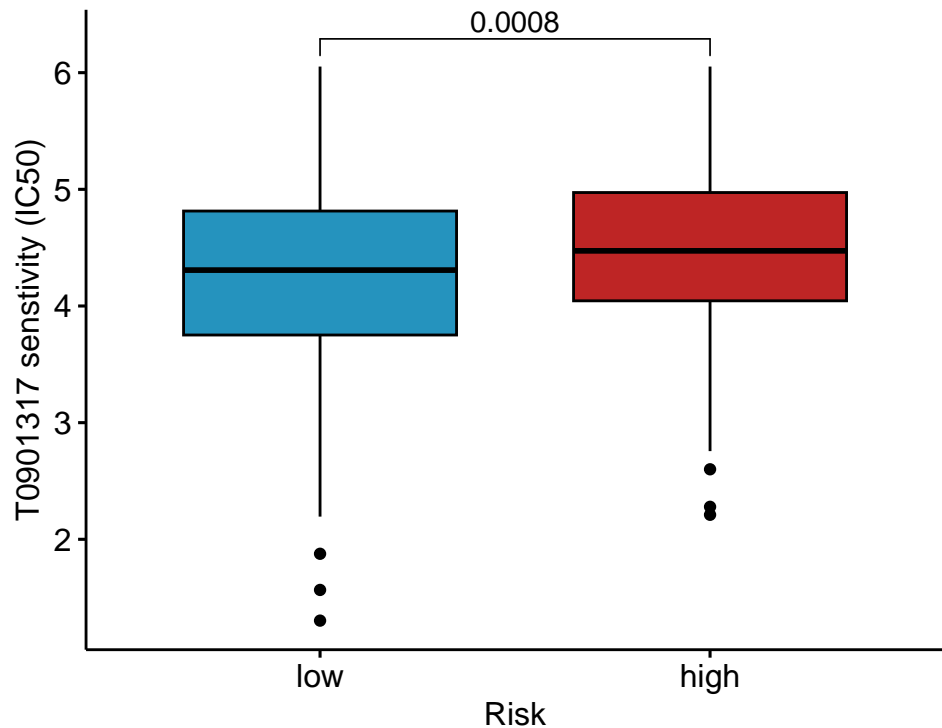

Supplement: Supplementary file 2 — Data S1. [file JCMM-28-e70059-s002.zip › Supplemental Material II/durgSenstivity.T0901317.pdf]

Risk 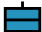 low 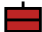 high

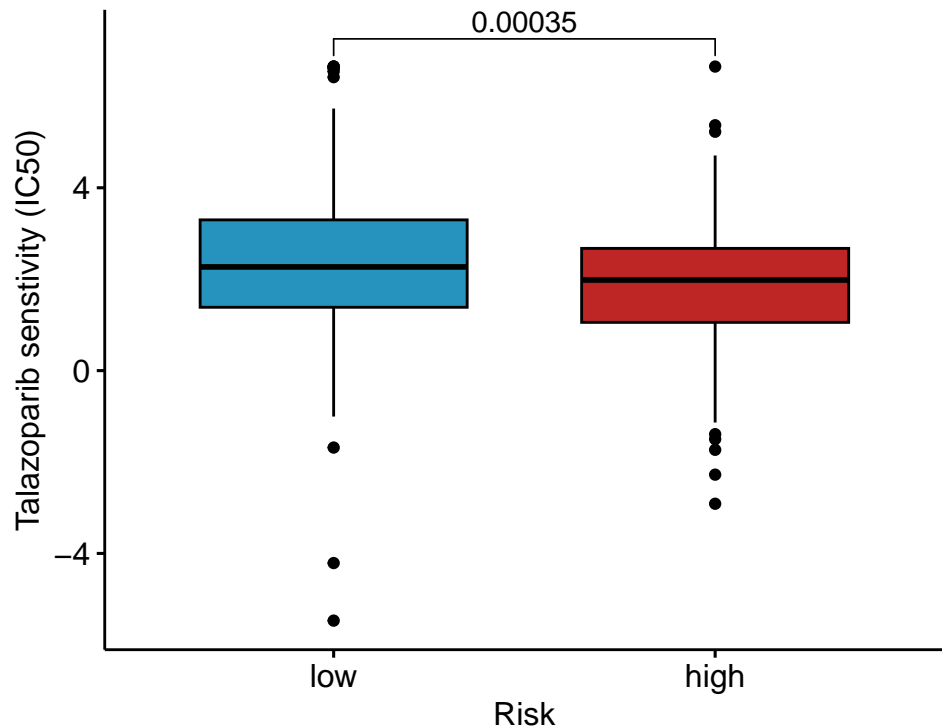

Supplement: Supplementary file 2 — Data S1. [file JCMM-28-e70059-s002.zip › Supplemental Material II/durgSenstivity.Talazoparib.pdf]

THZ-2-49 sensitivity (IC50)

Risk low high

0.00021

low

high

Risk

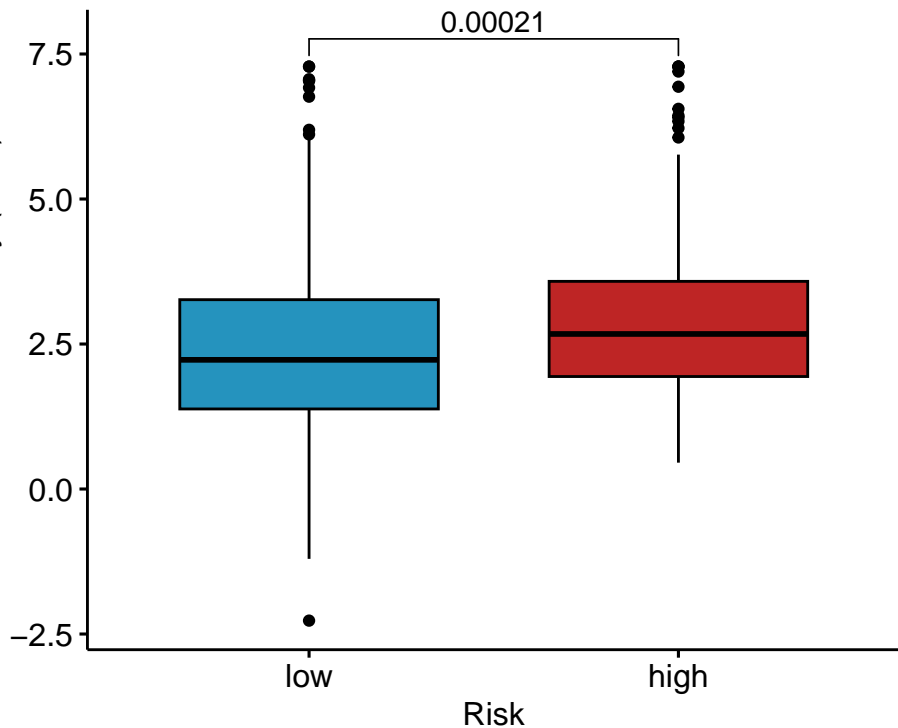

Supplement: Supplementary file 2 — Data S1. [file JCMM-28-e70059-s002.zip › Supplemental Material II/durgSenstivity.THZ-2-49.pdf]

Risk low high

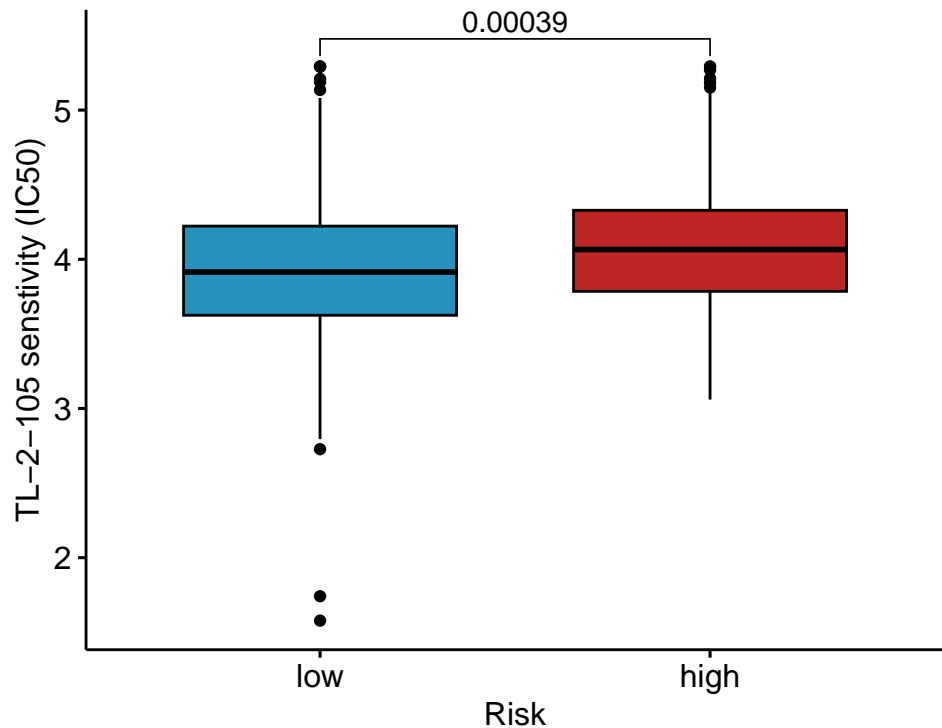

Supplement: Supplementary file 2 — Data S1. [file JCMM-28-e70059-s002.zip › Supplemental Material II/durgSenstivity.TL-2-105.pdf]

Risk low high

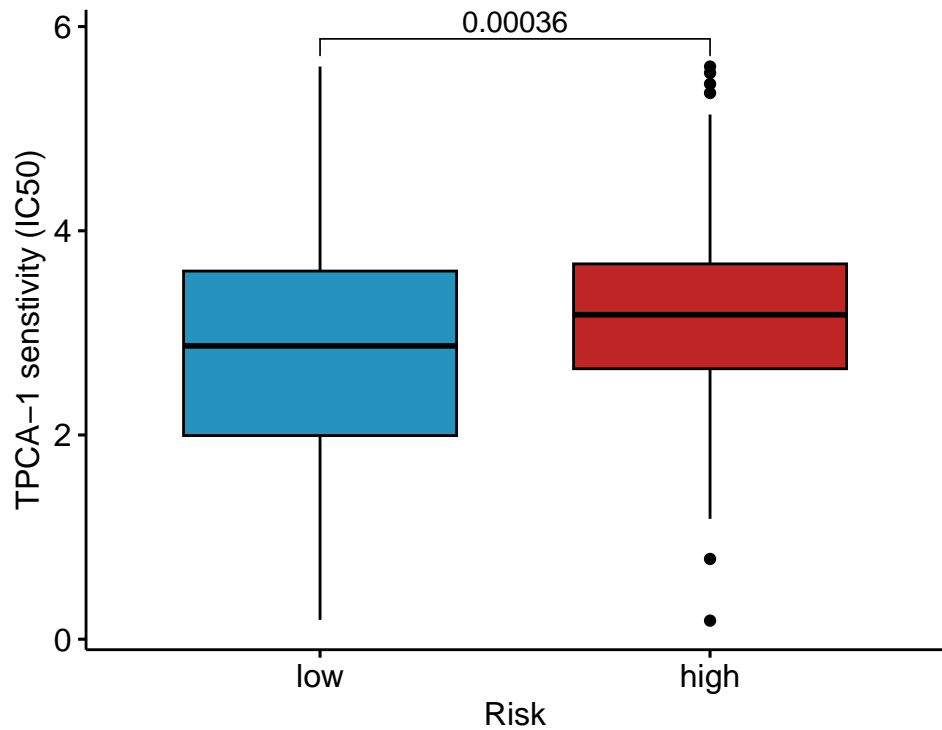

Supplement: Supplementary file 2 — Data S1. [file JCMM-28-e70059-s002.zip › Supplemental Material II/durgSenstivity.TPCA-1.pdf]

Risk 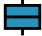 low 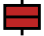 high

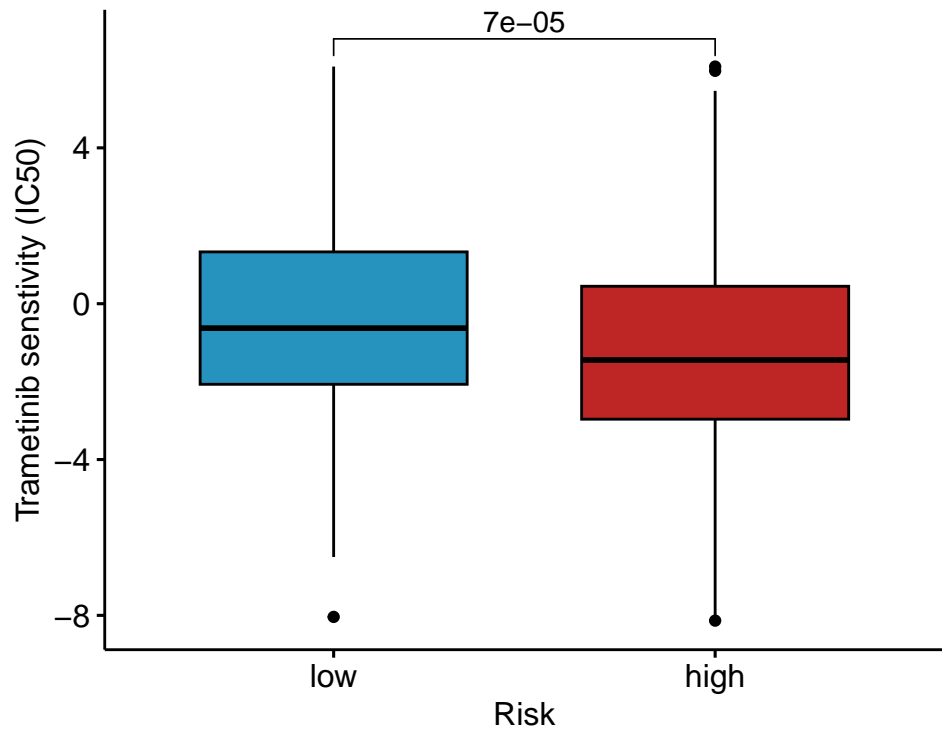

Supplement: Supplementary file 2 — Data S1. [file JCMM-28-e70059-s002.zip › Supplemental Material II/durgSenstivity.Trametinib.pdf]

Risk 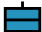 low 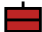 high

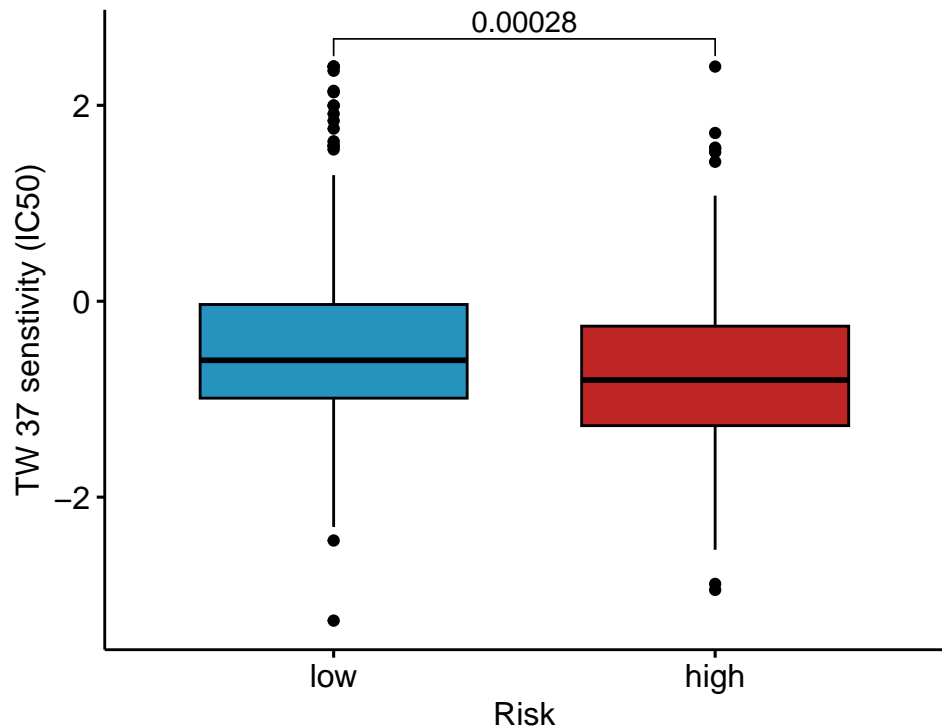

Supplement: Supplementary file 2 — Data S1. [file JCMM-28-e70059-s002.zip › Supplemental Material II/durgSenstivity.TW 37.pdf]

Risk 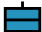 low 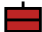 high

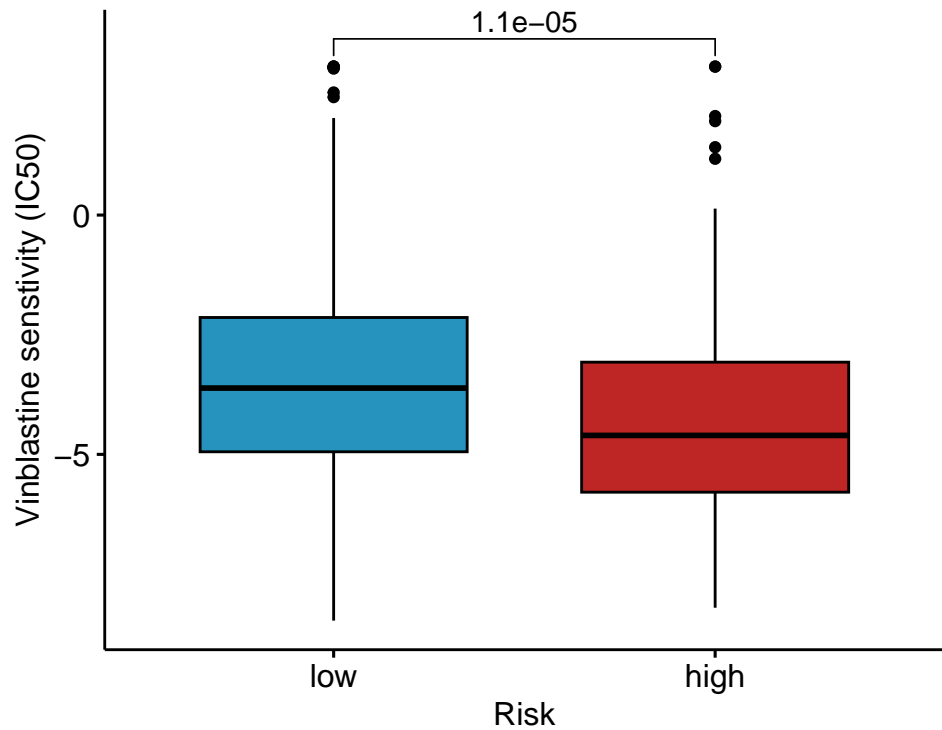

Supplement: Supplementary file 2 — Data S1. [file JCMM-28-e70059-s002.zip › Supplemental Material II/durgSenstivity.Vinblastine.pdf]

Risk 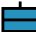 low 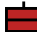 high

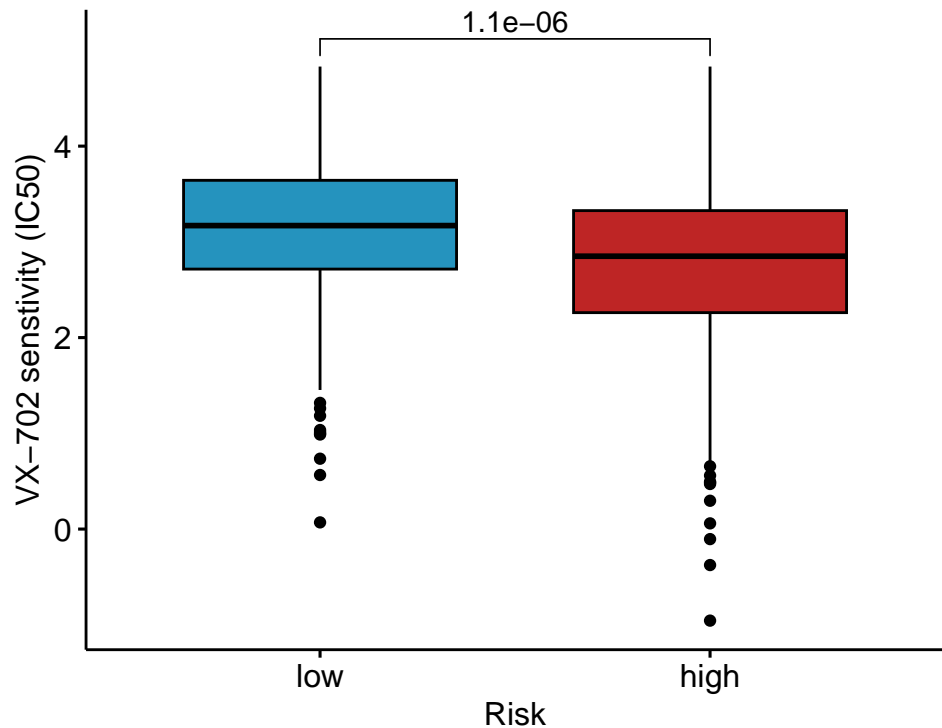

Supplement: Supplementary file 2 — Data S1. [file JCMM-28-e70059-s002.zip › Supplemental Material II/durgSenstivity.VX-702.pdf]

Risk 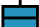 low 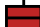 high

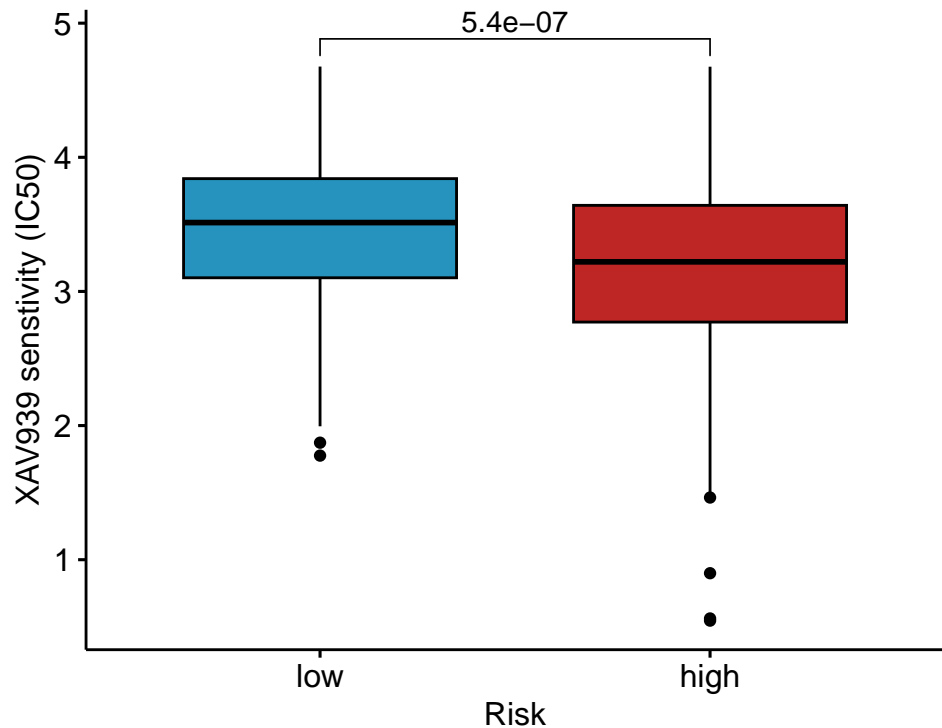

Supplement: Supplementary file 2 — Data S1. [file JCMM-28-e70059-s002.zip › Supplemental Material II/durgSenstivity.XAV939.pdf]

Risk 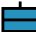 low 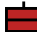 high

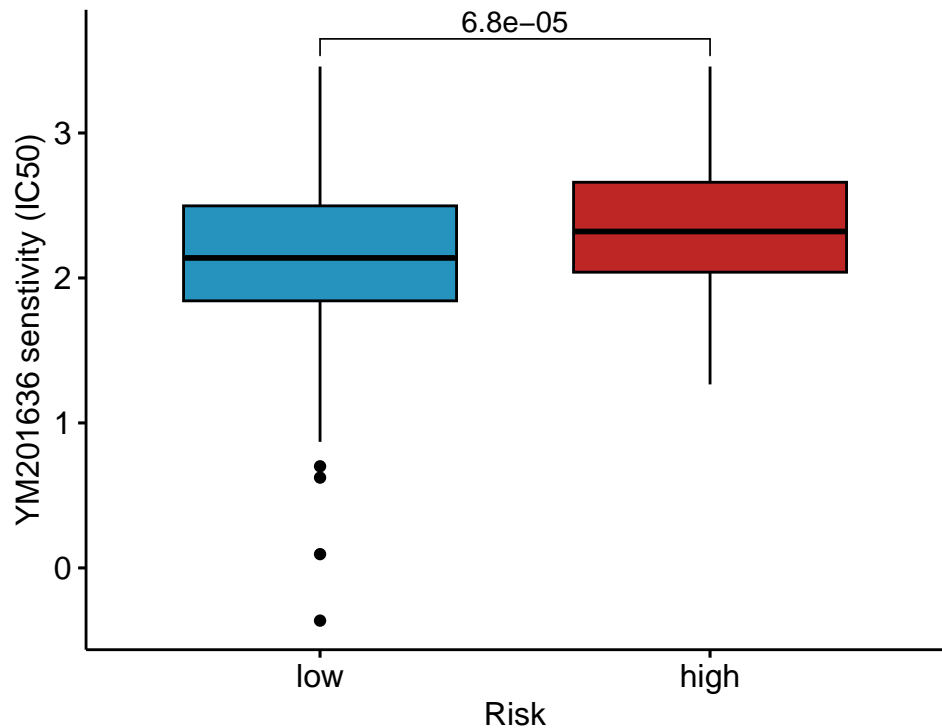

Supplement: Supplementary file 2 — Data S1. [file JCMM-28-e70059-s002.zip › Supplemental Material II/durgSenstivity.YM201636.pdf]
